# Osteoblasts are “educated” by crosstalk with metastatic breast cancer cells in the bone tumor microenvironment

**DOI:** 10.1186/s13058-019-1117-0

**Published:** 2019-02-27

**Authors:** Alexus D. Kolb, Alison B. Shupp, Dimpi Mukhopadhyay, Frank C. Marini, Karen M. Bussard

**Affiliations:** 1 Department of Cancer Biology, Sidney Kimmel Cancer Center, Thomas Jefferson University, Philadelphia, PA USA; 2 Comprehensive Cancer Center Wake Forest University and Wake Forest Institute of Regenerative Medicine, Winston-Salem, NC USA

**Keywords:** Osteoblast, Breast cancer cell, Bone, Metastasis, Proliferation, Tumor microenvironment

## Abstract

**Introduction:**

In a cancer-free environment in the adult, the skeleton continuously undergoes remodeling. Bone-resorbing osteoclasts excavate erosion cavities, and bone-depositing osteoblasts synthesize osteoid matrix that forms new bone, with no net bone gain or loss. When metastatic breast cancer cells invade the bone, this balance is disrupted. Patients with bone metastatic breast cancer frequently suffer from osteolytic bone lesions that elicit severe bone pain and fractures. Bisphosphonate treatments are not curative. Under ideal circumstances, osteoblasts would synthesize new matrix to fill in erosion cavities caused by osteoclasts, but this is not what occurs. Our prior evidence demonstrated that osteoblasts are diverted from laying down bone matrix to producing cytokines that facilitate breast cancer cell maintenance in late-stage disease. Here, we have new evidence to suggest that there are subpopulations of osteoblasts in the tumor niche as evidenced by their protein marker expression that have distinct roles in tumor progression in the bone.

**Methods:**

Tumor-bearing tibia of mice was interrogated by immunofluorescent staining for the presence of osteoblasts and alterations in niche protein expression. De-identified tissue from patients with bone metastatic breast cancer was analyzed for osteoblast subpopulations via multi-plex immunofluorescent staining. Effects of breast cancer cells on osteoblasts were recapitulated in vitro by osteoblast exposure to breast cancer-conditioned medium. Triple-negative and estrogen receptor-positive breast cancer proliferation, cell cycle, and p21 expression were assessed upon contact with “educated” osteoblasts.

**Results:**

A subpopulation of osteoblasts was identified in the bone tumor microenvironment in vivo of both humans and mice with bone metastatic breast cancer that express RUNX2/OCN/OPN but is negative for IL-6 and alpha-smooth muscle actin. These tumor “educated” osteoblasts (EOs) have altered properties compared to “uneducated” osteoblasts and suppress both triple-negative and estrogen receptor-positive breast cancer cell proliferation and increase cancer cell p21 expression. EO effects on breast cancer proliferation were mediated by NOV and decorin. Importantly, the presence of EO cells in the tibia of mice bearing tumors led to increased amounts of alkaline phosphatase and suppressed the expression of inflammatory cytokines in vivo.

**Conclusions:**

Our work reveals that there is a subpopulation of osteoblasts in the bone tumor microenvironment that demonstrate a functional role in retarding breast cancer cell growth.

**Electronic supplementary material:**

The online version of this article (10.1186/s13058-019-1117-0) contains supplementary material, which is available to authorized users.

## Introduction

Breast cancer is the second leading cause of cancer deaths and approximately one in eight women in the USA will develop breast cancer during their lifetime [1]. Breast cancer cells frequently metastasize to the bone, where the 5-year relative survival rate is < 10% [1].

In the adult skeleton, the bone is continually being remodeled. Under ideal circumstances, bone-resorbing osteoclasts excavate erosion cavities and bone-depositing osteoblasts synthesize matrix to form new bone, with no net bone loss or gain. Exceptions to these circumstances include (1) bone loss as a result of aging and osteoporosis [2, 3], (2) bone loss as a result of lack of physical activity or exercise [4–6], and (3) perturbation of normal bone remodeling by cancer bone metastases [7–10].

Patients that develop bone metastatic breast cancer have lesions that are either osteolytic [11, 12], osteoblastic, or a mix of osteoblastic and osteolytic lesions [13]. The vast majority of bone metastatic breast cancer lesions are osteolytic, where bone resorption occurs at a rate faster than bone deposition [14, 15]. Osteolytic bone lesions are frequently associated with severe bone pain, hypercalcemia, and skeletal-related events such as fractures and spinal cord compression [1]. On the other hand, metastases leading to an increase in bone deposition are considered osteoblastic [16]. Interestingly, exact mechanisms that elicit the formation of osteoblastic lesions in bone metastatic breast cancer are not fully known [9, 14]. However, one study identified the bone-specific transcriptional regulator RUNX2 as a key factor in events associated with osteoblastic lesions [16].

In the late stages of bone metastatic breast cancer, osteoclasts are constitutively activated, yet osteoblasts do not deposit new bone leading to overall net bone loss [17]. These events are characteristic of osteolytic disease [14, 18, 19]. In particular, patients with osteolytic bone metastatic breast cancer are often treated with bisphosphonate therapies, such as ibandronate [20–22] and zoledronic acid [23], which are aimed at impairing the activity of bone-resorbing osteoclasts. While this strategy may be initially effective, recent studies suggest that the addition of bisphosphonates to standard adjuvant therapies does not extend disease-free survival for women with osteolytic bone metastatic breast cancer, who will ultimately succumb to skeletal metastases [24]. This is, in part, due to the sustained bone resorption and an inability of osteoblasts to lay down new bone matrix [9]. These results suggest that osteoblasts may be altered or experience a loss of function in the tumor microenvironment. Currently, no drugs are available that directly stimulate osteoblast activity or promote bone deposition.

Data have demonstrated that metastatic breast cancer cells alter osteoblast properties in the late stages of the disease, including decreased proliferation and altered adhesion [25, 26]. Our laboratory and colleagues have found that osteoblasts are profoundly altered by breast cancer metastases and no longer differentiate [25]. Instead, the osteoblasts are diverted from depositing new matrix to producing cytokines implicated in cancer cell maintenance [7, 27–31]. Our laboratory also found that osteoblasts are altered by breast cancer metastases in late-stage disease and undergo a stress response to produce a classic set of cytokines that are maintenance factors for metastatic breast cancer cells: IL-6, IL-8, MCP-1, GRO-alpha, and VEGF [32, 33]. These cytokines facilitate breast cancer cell colonization in late-stage disease [32, 33]. We further believe that osteoblast-derived factors contribute to osteoblast autocrine and osteoblast-breast cancer cell paracrine mechanisms resulting in significant crosstalk between the two cell types during disease progression.

It is becoming increasingly evident that osteoblasts in the bone microenvironment play vital roles in cancer cell attraction [34, 35], maintenance [33], and survival [19, 32, 36] during cancer progression in the bone. It was demonstrated that osteoblast-derived TGF-beta increased PC-3U prostate cancer cell migration [35]. Furthermore, osteoblast-derived CXCL12 mediated bone metastatic prostate cancer progression via binding of its receptor CXCR4 on the cancer cells [11, 34, 37, 38]. Additionally, osteoblast expression of c-met and VEGFR2 promoted PC-3 and C4-2B prostate cancer growth in the bone [36]. We and our colleagues previously demonstrated that osteoblasts are profoundly altered by late-stage breast cancer metastases and experience altered adhesion and loss of differentiation capabilities [25, 26]. Furthermore, we found that osteoblasts are diverted from depositing new osteoid matrix and instead are directed by metastatic breast cancer cells to increase osteoblast production of cytokines that facilitate breast cancer cell colonization in the bone niche in late-stage disease [32, 33]. Collectively, these data suggest that osteoblasts have multiple roles in cancer progression and may interact differently with cancer cells depending on the stage of the disease. In the study described here, we sought to dissect specific interactions between osteoblasts and breast cancer cells and determine how these interactions affect breast cancer progression in the bone. We found that breast cancer cells act on osteoblasts in the tumor niche in vivo earlier in the metastatic process and alter the protein expression of a population of osteoblasts. We identified two different subpopulations of osteoblasts in the tumor niche in vivo based on their protein marker expression. We provide evidence that the two populations consist of one group that is “educated” by breast cancer cells (“educated” osteoblasts) and a second group that is not (“uneducated” osteoblasts). We define “educated” osteoblasts (EOs) as osteoblasts that have engaged in crosstalk with metastatic breast cancer cells by direct or indirect means. We define “uneducated” osteoblasts as osteoblasts that have not communicated with metastatic breast cancer cells. We also hypothesized that the presence of EOs in the bone tumor microenvironment would lead to unique protein expression of factors involved in inflammation, bone turnover, and extracellular matrix remodeling. Using an intratibial model of bone metastasis, we showed that the inflammatory cytokine IL-6 as well as matrix remodeling factors MMP3 and type I collagen were reduced in the endosteal and hematopoietic niches of the tibia with tumors composed of an admix of EOs plus triple-negative breast cancer cells, as compared to admixes of “uneducated” osteoblasts plus triple-negative breast cancer cells, or triple-negative breast cancer cells injected alone. Furthermore, alkaline phosphatase, a marker of osteoblast differentiation [39], was increased in the endosteal niche of the tibia with tumors composed of EOs plus triple-negative breast cancer cells, as compared to admixes of “uneducated” osteoblasts plus triple-negative breast cancer cells, or triple-negative breast cancer cells injected alone. In addition, we demonstrated that exposure to EO-conditioned medium reduces breast cancer cell proliferation and leads to a reduction in the number of cells in the S phase of the cell cycle of both triple-negative and estrogen receptor-positive (ER+) breast cancer cells in vitro. We found that this effect was mediated in part by an antibody to NOV (CCN3) or recombinant decorin protein. Furthermore, direct co-culture with EOs increased triple-negative and ER+ breast cancer expression of p21 compared to cultures with “uneducated” osteoblasts in vitro. Thus, our data suggest that osteoblasts may be “educated” by breast cancer cells in vivo to alter osteoblast protein expression. Our data further suggest that there is a subpopulation of osteoblasts that demonstrate a functional role in retarding breast cancer growth.

## Materials and methods

### Cells

All cells tested negative for *Mycoplasma* spp. infection using a MycoSensor PCR Assay kit (Agilent Technologies, Santa Clara, CA).

#### Osteoblasts

MC3T3-E1 cells, a murine pre-osteoblast line capable of differentiation and mineralization in culture [40] (Dr. Norman Karin, Roswell Park Cancer Institute), were maintained in alpha Minimum Essential Medium (αMEM) (Gibco, Grand Island, NY), 10% neonatal FBS (HyClone, Logan, UT), and penicillin 100 U/ml/streptomycin 100 μg/ml (Gibco). Twenty-four hours later, the medium was replaced with 1× differentiation medium (αMEM, 10% neonatal FBS, penicillin 100 U/ml/streptomycin 100 μg/ml, 50 μg/ml ascorbic acid (Sigma, St. Louis, MO), and 10 mM β-glycerophosphate (Sigma, St. Louis, MO)). MC3T3-E1 cells were grown to two stages of differentiation: early differentiation (10 days) or late differentiation (20 days) and were used at passage ≤ 20 [41]. Differentiation medium was exchanged every third day. Cells were cultured in a humidified chamber of 5% CO_2_ and 95% air at 37 °C.

NHOst human primary osteoblasts derived from a single donor with no evidence of disease were purchased directly from Lonza (Walkersville, MD). NHOst cells were maintained in a growth media of osteoblast basal medium plus FBS, ascorbic acid, and gentamicin/amphotericin-B (Lonza). Media were exchanged every other day. Cells were cultured in a humidified chamber of 5% CO_2_ and 95% air at 37 °C.

#### Mouse fibroblasts

NIH-3T3 murine fibroblast cells are a mesenchymal cell line established from NIH Swiss mouse primary embryo cultures [42]. These cells were a gift from Dr. Andrea Mastro, The Pennsylvania State University. Media were exchanged every other day. NIH-3T3 cells were maintained in a growth media of alpha-MEM (Gibco), 10% FBS (Hyclone), and penicillin 100 U/ml/streptomycin 100 μg/ml (Gibco). Cells were cultured in a humidified chamber of 5% CO_2_ and 95% air at 37 °C.

#### Human mammary epithelial cells

hTERT-HME1 human mammary epithelial cells were derived from a patient undergoing reduction mammoplasty with no history of breast cancer. The human mammary epithelial cells were immortalized by infection with pBabepuro-hTERT vector retrovirus [43]. hTERT-HME1 cells were maintained in mammary epithelial cell growth medium (MEBM) supplemented with bovine pituitary extract, hydrocortisone, human epidermal growth factor (10 μg/ml), 0.5% recombinant human insulin, and gentamicin/amphotericin-B (Lonza). hTERT-HME1 cells were purchased from the ATCC (Manassas, VA). Cells were cultured in a humidified chamber of 5% CO_2_ and 95% air at 37 °C.

#### Cancer cells

MDA-MB-231 human metastatic breast cancer cells were derived from a pleural effusion of an adenocarcinoma [44]. MDA-MB-231BRMS human metastasis-suppressed cells are the isologous line in which metastasis is suppressed to the bone as well as to the other organs by transfection of the BRMS1 gene [45, 46] and were a gift from Dr. Danny Welch, Kansas University Medical Center. MDA-MB-231 and MDA-MB-231BRMS cells were maintained in a breast cancer growth medium of DMEM (Gibco), 5% neonatal FBS, and 1% penicillin 100 U/ml/streptomycin 100 μg/ml. Cells were cultured in a humidified chamber of 5% CO_2_ and 95% air at 37 °C. MCF-7 human ER+ breast cancer cells were derived from a pleural effusion [47] and were purchased directly from the ATCC (Manassas, VA). MCF-7 cells were maintained in EMEM (Gibco) supplemented with 10% FBS (Hyclone), 100 U/ml penicillin 100 mg/ml streptomycin (Gibco), and 0.01 μg/ml of recombinant human insulin (MP Biomedicals, Solon, OH).

For in vivo experiments, cell lines expressing the green fluorescent protein (GFP) and luciferase (pLeGo-IG2-Luc2 vector) were utilized and were a gift from Dr. Alessandro Fatatis, Drexel University. MDA-MB-231GFP/luciferase cells are analogous to MDA-MB-231 cells but have been engineered to express GFP and the Luc2 vector [48].

### Conditioned media

MC3T3-E1 cells, grown for 10 or 20 days, were rinsed with phosphate-buffered saline (PBS) and serum-free αMEM added (20 ml per T-150 flask, ~ 9.1 × 10^4^ cells/cm^2^) for 24 h. Osteoblast-conditioned medium (OBCM) was collected, centrifuged to remove cellular debris, and stored at − 80 °C.

MDA-MB-231 triple-negative metastatic breast cancer, MDA-MB-231BRMS metastasis-suppressed breast cancer cells, MCF-7 ER+ breast cancer cells, or hTERT-HME1 human mammary epithelial cells were rinsed with PBS and serum-free αMEM added (~ 1.3 × 10^5^ cells/cm^2^). Twenty-four hours later, breast cancer cell-conditioned medium (BCCM) or hTERT-HME1-conditioned medium was collected, centrifuged to remove cellular debris, and stored at − 80 °C.

### Generation of EOs in vitro

Differentiated MC3T3-E1 cells were rinsed and treated with either BCCM or hTERT-HME1-conditioned media treatment formulation: 3 parts 1.5× differentiation medium (αMEM, 15% neonatal FBS, 75 μg/ml ascorbic acid (Sigma), 15 mM β-glycerophosphate (Sigma), and penicillin 100 U/ml/streptomycin 100 μg/ml) plus 1 part either MDA-MB-231, MDA-MB-231BRMS, or MCF-7 breast cancer-conditioned medium; or hTERT-HME1 mammary epithelial cell-conditioned medium for an additional 21 days [49] (days 31 or 41, respectively). Media were changed every second day. Vehicle medium (VM) consisting of MC3T3-E1 differentiation medium was used for comparison.

### EO-conditioned media

EO cells were rinsed with PBS and serum-free αMEM added. Twenty-four hours later, EO cell-conditioned media were collected, centrifuged to remove cellular debris, and stored at − 80 °C.

### Alkaline phosphatase staining

Bone alkaline phosphatase is a biochemical marker of osteoblast differentiation in vitro and bone turnover in vivo [50]. Twenty-day differentiated EO cells were plated at 1 × 10^5^ cells/cm^2^ in EO cell growth medium and grown to confluence. The medium was exchanged every third day. For MC3T3-E1 cells (control), the cells were plated at 1 × 10^5^ cells/cm^2^ in a MC3T3-E1 growth medium. Twenty-four hours later, the medium was exchanged for MC3T3-E1 differentiation medium. Cells were grown for 20 days (late differentiation). The medium was exchanged every third day. To stain for alkaline phosphatase, growth media were removed, cells washed with PBS, and fixed for 10 min with 4% paraformaldehyde (PFA; Electron Microscopy Sciences, Hatfield, PA). The cells were rinsed with PBS in three sequential washes, and cells covered with alkaline phosphatase stain (1.3 mg Napthol AS-BI Phosphate (Sigma), 0.2 M Tris, pH 8.5 (Sigma), and 7.5 mg Fast Blue RR Salt (Sigma) in a total volume of 13 ml). The stain was filtered and cells incubated for 30 min at 37 °C. Cells were rinsed and photographed using a light microscope.

### Mineralization

To assay for the state of osteoblast mineralization, EO cells were stained for Von Kossa, a biochemical marker of bone mineralization [51, 52]. Twenty-day differentiated EO cells were plated at 1 × 10^5^ cells/cm^2^ in EO cell growth medium and grown to confluence. The medium was exchanged every third day. For MC3T3-E1 cells (control), the cells were plated at 1 × 10^5^ cells/cm^2^ in a MC3T3-E1 growth medium. Twenty-four hours later, the medium was exchanged for MC3T3-E1 differentiation medium. Cells were grown for 20 days (late differentiation). The medium was exchanged every third day. To stain for Von Kossa, growth media were removed, cells washed with PBS, and fixed for 10 min with 10% formalin (VWR). Formalin was removed, cells were rinsed with PBS in three sequential washes, then cells were incubated with 5% silver nitrate (Sigma) for 30 min at room temperature in the dark. Cells were then rinsed with dH_2_O and photographed using a light microscope.

### Western blotting

Cells were lysed in an ice-cold RIPA lysis buffer containing 50 mM Tris-HCl (pH 7.4, Sigma), 1% NP-40 (*v*/*v*, Thermo Scientific, Waltham, MA), 0.25% Na-deoxycholate (*v*/*v*, Sigma), 150 mM NaCl (Sigma), 1 mM EDTA (Sigma), 1 mM PMSF (Sigma), 1 mM Na_3_VO_4_ (Sigma), and 1 mM NaF (Sigma) plus Halt™ Protease and Phosphatase Inhibitor Cocktail (Thermo Scientific), then gently agitated for 30 min at 4 °C. Next, lysates were centrifuged for 20 min at 14,000 rpm at 4 °C, quantified using a *DC™* Protein Assay (Bio-Rad, Hercules, CA), boiled with loading buffer, then loaded onto a 12% SDS-PAGE gel (Bio-Rad). Separated proteins were transblotted onto Immobilon-P polyvinylidene difluoride membranes (EMD Millipore, Billerica, MA). The membranes were blocked using either Tris-buffered saline (TBS)-Tween (TBS-T, 1× TBS plus 5% Tween-20 (*v*/*v*, Sigma)) containing 5% non-fat dry milk powder (Biotium, Hayward, CA) (osteopontin, alpha-smooth muscle actin, type I collagen, MCP-1, alpha-tubulin, and beta-actin), or SuperBlock Blocking buffer (Thermo Scientific) (alkaline phosphatase, p21, MMP3, VEGF, FSP, FOXN1, and IL-6) and incubated with primary antibodies overnight at 4 °C. Primary antibodies included goat anti-mouse IL-6 (0.5 μg/ml; R&D Systems, Minneapolis, MN), rabbit anti-mouse MCP-1 (1:10,000; Abcam, Cambridge, MA), rabbit anti-mouse/human osteopontin (1:500; Abcam), rabbit anti-mouse/human alkaline phosphatase (1:10,000; Abcam), goat anti-mouse fibroblast-specific protein (FSP) (0.5 μg/ml; R&D Systems), goat anti-mouse VEGF (0.1 μg/ml; R&D Systems), rabbit anti-mouse MMP3 (1:2000; Lifespan Biosciences, Seattle, WA), rabbit anti-collagen type I (1:1000; Bio-Rad), mouse anti-mouse/human alpha-SMA (1:50; Abcam), rabbit anti-human p21 (1:100; Cell Signaling, Danvers, MA), rabbit anti-mouse FOXN1 (1:300; Bioss, Woburn, MA), mouse anti-α-tubulin (1:3000; Sigma), and mouse anti-beta actin (1:5000; Sigma). Secondary antibodies included horse anti-mouse HRP (1:1000; Cell Signaling), goat anti-rabbit HRP (1:1000; Cell Signaling), and donkey anti-goat HRP (1:5000; Abcam). Signals were detected using SuperSignal™ West Femto Chemiluminescent Substrate detection kit (Thermo Scientific)**.**

### EO proliferation assay

Cells were plated in at 1 × 10^5^ cells/cm^2^ in 35 × 10 mm dishes. Beginning on day 2 after plating, and continuing every other day for 10 days, cells were detached and counted using a hemocytometer. Three individual replicates per time point were counted per condition.

### Breast cancer proliferation assay

Breast cancer cells were plated in breast cancer growth media at 1 × 10^5^ cells/cm^2^ in 35 × 10 mm dishes. Twenty-four hours later (day 0), growth media were removed and cancer cells treated with 1 ml breast cancer growth media plus either (a) MC3T3-E1 osteoblast conditioned media (OBCM), (b) EO-conditioned media, or (c) the respective breast cancer-conditioned media (BCCM). Breast cancer cells were then counted beginning on day 1 after plating and continuing every day for 5 days, cells were detached and counted using a hemocytometer. Three individual replicates per time point were counted per condition.

To examine the effect of decorin and NOV on breast cancer proliferation, breast cancer cells were plated in breast cancer growth media at 1 × 10^5^ cells/cm^2^ in 35 × 10 mm dishes. Twenty-four hours later (day 0), growth media were removed and cancer cells treated with 1 ml breast cancer growth media plus either 1 ml (a) EO-conditioned media *plus* either 5 μg/ml anti-NOV (R&D Systems) or 5 ng/ml recombinant decorin protein (R&D Systems), (b) EO-conditioned media *plus* 5 μg/ml anti-NOV (R&D Systems) *plus* 15 ng/ml recombinant NOV protein (R&D Systems), (c) EO-conditioned media *plus* 5 ng/ml recombinant decorin protein (R&D Systems) *plus* 5 μg/ml anti-decorin (R&D Systems), (d) EO-conditioned media, or (e) MC3T3-E1-conditioned media (OBCM). Breast cancer cells were then counted beginning on day 1 after plating and continuing every day for 5 days, cells were detached and counted using a hemocytometer. Three individual replicates per time point were counted per condition.

### Propidium iodide staining

Breast cancer cells were plated in breast cancer growth media at 1 × 10^5^ cells/cm^2^ in 35 × 10 mm dishes. Twenty-four hours later, growth media were removed and cancer cells treated with 1 ml breast cancer growth media plus either (a) vehicle media (DMEM for MDA-MB-231; EMEM for MCF-7) (control) or (b) EO-conditioned media. Media were exchanged every day. Breast cancer cells were then detached and fixed for at least 2 h with 95% cold ethanol beginning on day 1 after plating and continuing every day for 5 days. Fixed cells were stored at − 20 °C until use. For propidium iodide staining, ethanol was decanted and fixed cells were washed once with PBS. Cells were resuspended in a solution of 50 ng/ml propidium iodide (Thermo Fisher), 100 ng/ml RNAse A (ThermoFisher), and PBS per 1 × 10^6^ cells and incubated for 30 min in the dark at room temperature. Stained cells were then analyzed for propidium iodide staining using a Becton Dickinson LSR II flow cytometer at excitation 535 nm and emission at 617 nm. A minimum of 10,000 events were counted per sample. Dead cells and debris were eliminated by forward and side scatter gating. Cell cycle phase was analyzed using BDFACS Diva software and FlowJo software. Three individual replicates per time point were counted per condition.

### EdU staining

Breast cancer cells were plated at 5 × 10^3^ cells/cm^2^ in 4-well chamber slides in culture media plus 0.5 μM 5-ethynyl-2-doxyuridine (EdU) for imaging using the Click-iT EdU Imaging Kit (Invitrogen; Carlsbad, CA) for 1, 2, 3, 4, and 5 days. Cells were maintained in media plus EdU for the entire length of the time course. At the end of the time course, the media containing EdU were removed and cells were washed in PBS, fixed with 4% paraformaldehyde for 10 min at room temperature, and then washed three times with PBS. For EdU imaging, cells were permeabilized with 0.2% Triton X-100 for 10 min at room temperature and subsequently washed with PBS. Cells were then incubated with the Click-iT reaction cocktail (prepared as described by the manufacturer) for 30 min at room temperature in the dark. The reaction cocktail was removed, the cells were washed three times with PBS, and the nuclei were stained for 4 min with 4',6-diamidino-2-phenylindole (DAPI; 0.2 ng/µl). After DAPI was removed, cells were washed again with PBS and visualized using fluorescence microscopy.

### F-actin staining

For phalloidin staining to elicit F-actin organization, cells were fixed for 10 min using 4% paraformaldehyde followed by washing with PBS. Cells were then stained with Alexa Fluor® 594 phalloidin (300 units; Life Technologies, Carlsbad, CA) in PBS for 20 min at room temperature. Following staining, cells were washed with PBS and stained with DAPI for 4 min. Cells were then washed and mounted with Fluoromount G (Southern Biotech, Birmingham, AL). Fluorescence images were obtained using an Olympus FV3000 microscope (Olympus) equipped with a Hamamatsu color camera (Hamamatsu Photonics, Iwata City, Japan), using a 100x silicone oil immersion objective. Image analysis was performed with Olympus cellSens. Quantification of stress fiber anisotropy was performed using an Olympus cellSens Count and Measure Tool.

### LC3 staining

MC3T3-E1, EO-231, and EO-BRMS cells were grown to ~ 70% confluence, then growth media removed, cells washed with PBS, then fixed in 100% ice-cold methanol for 15 min at − 20 °C. For a positive control, MC3T3-E1 osteoblasts were treated with serum-free media for 48 h [53], then the media were removed and cells washed with PBS then fixed in 100% ice-cold methanol for 15 min at − 20 °C. For all cells, methanol was removed and cells washed in PBS then blocked using Dako Universal Blocking buffer (Dako Products, Santa Clara, CA) for 1 h at room temperature followed by incubation overnight at 4 °C with rabbit anti-mouse LC3A/B antibody (1:800; Cell Signaling Technologies). Cells were then incubated with goat anti-rabbit 488 (1:3000; Biotium, Fremont, CA) for 1 h at room temperature, followed by staining with DAPI. Cells were then mounted with Fluoromount G (Southern Biotech). The sections without the primary antibody served as negative controls. Images were viewed using an Olympus FV 3000 fluorescent microscope using a 40x objective (Olympus) equipped with Hamamatsu color camera (Hamamatsu Photonics).

### Intratibial inoculations

MDA-MB-231GFP/Luc2 cells, MC3T3-E1 cells, and EO-231 cells, 90% confluent, were detached, washed, and resuspended in PBS. MDA-MB-231GFP/Luc2 cells were admixed with MC3T3-E1 or EO-231 cells at a 2:1 ratio, whereby a total of 5 × 10^5^ cells total in 10 μl PBS were injected into the tibias of female athymic mice aged 5–6 weeks (Harlan Sprague-Dawley, Indianapolis, IN). MDA-MB-231GFP/Luc2 cells inoculated alone served as controls (5 × 10^5^ cells in 10 μl PBS). Briefly, mice were anesthetized via an intraperitoneal injection of a mixture of ketamine (129 mg/kg) and xylazine (4 mg/kg). Once the mice were fully anesthetized as evidenced by a toe pinch and lack of movement, the hind leg was bent to a 90° position and 27 gauge needle with cells inserted through the patellar tendon and into the proximal tibia using gentle pressure and twisting motion [54]. The contralateral tibia was injected with PBS as a control. Six mice were utilized per experimental group. IVIS Imaging (Perkin Elmer, Waltham, MA) was used to monitor tumor formation for luciferase expression. Mice were euthanized via CO_2_ inhalation followed by cervical dislocation once tumors reached an average radiance (p/s/cm^2^/sr) of 1 × 10^8^. Mice were maintained under the guidelines of the NIH and Thomas Jefferson University. All protocols were approved and monitored by the Institutional Animal Care and Use Committee.

### In vivo imaging

For in vivo imaging, animals were injected with 100 μl of 30 mg/ml d-luciferin (Perkin Elmer) via intraperitoneal injection and anesthetized using 2.5% isoflurane. Animals were then transferred to the chamber of an IVIS Lumina XR (Perkin Elmer) where they received 2% isoflurane throughout the image acquisition. Ten to 15 min after injection of the substrate, exposures of both dorsal and ventral views were obtained along with X-ray, and quantification and analysis of bioluminescence was performed using Living Image software.

### Bone preparation for immunochemistry

Tibia were dissected from mice, then fixed for 24–48 h at 4 °C in 4% paraformaldehyde (Electron Microscopy Sciences), and decalcified for an additional 24–48 h at 4 °C with 0.5 mol/l EDTA in dH_2_O (Sigma) [5, 24]. For embedding, the bones were soaked in 30% sucrose in PBS for 24 h, placed in Shandon CryomatrixTM embedding medium (Thermo Shandon, Waltham, MA), and snap frozen in liquid nitrogen using the Gentle-Jane SnapFreezing technique (Instrumedics Inc., Hackensack, NJ). Frozen samples were wrapped in aluminum foil and stored at − 20 °C. CryoJane frozen section preparation cryosectioning was performed on a Leica CM3050 Cryostat (Leica, Inc., Nussloch, Germany). For sectioning, tibia were oriented with the end proximal to the knee pointed toward the blade. Ten-micron-thick longitudinal, serial sections were cut using a Diamond High Profile Knife (C.L. Sturkey, Lebanon, PA). Pre-chilled adhesive transfer tape windows (Leica Inc.) were used to transfer cut serial sections onto pre-chilled adhesive-coated slides (CJ4X adhesive-coated slides; Leica Inc.). Two bone serial sections were placed onto each slide. Transfer tape windows were removed from the slides at − 20 °C. The bone sections were permanently bonded to slides after 30 min of exposure to ultraviolet light. The slides were stored in slide boxes at − 20 °C until use.

### Immunochemistry of the murine tibia

The serial bone sections of tibia from mice were allowed to equilibrate to room temperature for at least 30 min prior to use. The sections were circled with an ImmEdge Hydrophobic Barrier Pen (Vector Laboratories, Burlingame, CA) and permeabilized for 10 min using 0.2% Triton-X (Sigma) in PBS. The sections were boiled for 30–45 s in 0.01 M sodium citrate buffer pH 6.0 for antigen retrieval. Non-specific binding was blocked with Dako Universal Blocking Buffer (Dako Products) for 1 h. The slides were incubated overnight at 4 °C with either mouse anti-alpha-smooth muscle actin (1:100, Abcam), rabbit-anti GFP (1:25, Invitrogen, Carlsbad, CA), rabbit anti-alkaline phosphatase (1:100, Abcam), rabbit anti-osteopontin (1:500, Abcam), rabbit anti-collagen type I (1:75, Bio-Rad), rabbit anti-MMP3 (1:75, Lifespan Biosciences, Seattle, WA), rabbit anti-MCP-1 (10 μg/ml, Abcam), goat anti-VEGF (25 μg/ml, R&D Systems), goat anti-IL-6 (40 μg/ml, R&D Systems), rabbit anti-mouse FOXN1 (1:25, Bioss), rabbit anti-mouse RUNX2 (1:500, Abcam), goat anti-mouse decorin (40 μg/ml, R&D Systems), or rat anti-mouse NOV (40 μg/ml, R&D Systems). Next, the slides were incubated for 1 h at room temperature with either donkey anti-mouse 594, chicken anti-rabbit 594, chicken anti-goat 594, donkey anti-rat 594, or goat anti-rabbit 488 (1:1000, Biotium). Sections were stained with DAPI (0.2 ng/μl) then mounted with Fluoromount G (Southern Biotech). The sections without the primary antibody and non-cancer-bearing murine bones served as negative controls. Images were viewed using an Olympus FV 3000 fluorescent microscope (Olympus) equipped with Hamamatsu color camera (Hamamatsu Photonics). Fluorescent images were analyzed using the Adaptive Threshold and Count and Measure function on the Olympus cellSens software. At least three independent, serial sections were stained per bone, and three bones examined per condition.

### Immunochemistry of human osteoblasts

Human NHOst cells were grown to confluence in 4-well chamber slides (Sarstedt, Numbrecht, Germany), fixed for 10 min at room temperature with 4% paraformaldehyde (EMD Biosciences), then circled with an ImmEdge Hydrophobic Barrier Pen (Vector Laboratories). Next, cells were permeabilized for 10 min using 0.2% Triton-X (Sigma) in PBS. Non-specific binding was blocked with Dako Universal Blocking Buffer (Dako Products) for 1 h at room temperature. The slides were incubated overnight at 4 °C with either mouse anti-human osteocalcin (R&D Systems, 10 μg/ml), rabbit anti-human RUNX2 (Abcam, 1:500), mouse anti-human alpha-smooth muscle actin (1:100, Abcam), rabbit anti-human alkaline phosphatase (1:100, Abcam), rabbit anti-human osteopontin (1:500, Abcam), or goat anti-human IL-6 (40 μg/mL, R&D Systems), Next, slides were incubated for 1 h at room temperature with either donkey anti-mouse 594, chicken anti-rabbit 594, donkey anti-goat 488, or goat anti-rabbit 488 (1:1000, Biotium). Cells were stained with DAPI (0.2 ng/μl) then mounted with Fluoromount G (Southern Biotech). The cells without the primary antibody served as negative controls. Images were viewed using an Olympus FV 3000 fluorescent microscope (Olympus) equipped with Hamamatsu color camera (Hamamatsu Photonics).

### Multi-plex immunochemistry of human bone samples

De-identified human tissue specimens from patients with bone metastatic breast cancer undergoing total hip replacement at Thomas Jefferson University were collected from consented patients, immediately put on ice, and processed within 20 min of extraction. Patients were between the ages of 70 and 77 years old and were diagnosed with ER+ bone metastases. All patients received Herceptin as a primary line of treatment. Samples were embedded in paraffin and sent to the Thomas Jefferson University Histology Core for sectioning. Samples were serially cut at 6 μm and bound to Colorfrost Plus slides (Thermo Scientific). To deparaffinize and rehydrate, the sections were taken through a series of xylenes, decreasing concentrations of alcohols, followed by water and TBS. Sections were circled with an ImmEdge Hydrophobic Barrier Pen (Vector Laboratories) and permeabilized for 10 min using 0.2% Triton-X (Sigma) in PBS. The sections were boiled for 30–45 s in 0.01 M sodium citrate buffer pH 6.0 for antigen retrieval. Non-specific binding was blocked with Dako Universal Blocking Buffer (Dako Products) for 1 h. The slides were first incubated overnight at 4 °C with either rabbit anti-human RUNX2 (Abcam, 1:500), rabbit anti-human osteopontin (1:75, Abcam), or goat anti-human decorin (40 μg/ml, R&D Systems), followed by incubation for 1 h with either goat anti-rabbit 488, chicken anti-rabbit 594, or donkey anti-goat 488 (1:1000, Biotium), respectively. Next, the slides were again blocked with Dako Universal Blocking Buffer (Dako Products) for 1 h at room temperature followed by overnight incubation at 4 °C with either mouse anti-human osteocalcin (R&D Systems, 10 μg/ml), rabbit anti-human RUNX2 (1:500, Abcam), or goat anti-human NOV (40 μg/ml, R&D Systems). Next, the slides were incubated for 1 h at room temperature with either chicken anti-rabbit 594, chicken anti-rabbit 488, or donkey anti-goat 594 (1:1000, Biotium). Sections were stained with DAPI (0.2 ng/μl) then mounted with Fluoromount G (Southern Biotech). The sections without the primary antibody served as negative controls. Images were viewed using an Olympus FV 3000 fluorescent microscope (Olympus) equipped with Hamamatsu color camera (Hamamatsu Photonics).

To carry out the multi-plex immunofluorescence, coverslips were first removed from mounted slides by soaking in PBS for 30 min at room temperature. Next, the slides were boiled for 3 min in 0.01 M sodium citrate buffer pH 6.0 to quench the first set of fluorophores. The slides were allowed to cool in water followed by TBS, then blocked using Dako Universal Blocking Buffer for 1 h. Slides were first incubated overnight at 4 °C with goat anti-human IL-6 (40 μg/ml, R&D Systems), followed by incubation for 1 h with donkey anti-goat 488 (1:1000, Biotium). Next, the slides were again blocked with Dako Universal Blocking Buffer (Dako Products) for 1 h at room temperature followed by overnight incubation at 4 °C with mouse anti-human alpha-smooth muscle actin (1:100, Abcam). Next, the slides were incubated for 1 h at room temperature with donkey anti-mouse 594 (1:1000, Biotium). Slides were then washed with TBS, and sections stained with DAPI (0.2 ng/μl). Slides were then mounted with Fluoromount G (Southern Biotech). Sections without the primary antibody served as negative controls. Images were viewed using an Olympus FV 3000 fluorescent microscope (Olympus) equipped with Hamamatsu color camera (Hamamatsu Photonics). IL-6 and alpha-SMA were false colored using cellSens (IL-6, 488 [green] false colored to purple; alpha-SMA, 594 [red] false colored to yellow). Fluorescent images were analyzed using the Adaptive Threshold and Count and Measure function on the Olympus cellSens software.

### Statistical analysis

Statistical analyses were carried out using GraphPad Prism 7 (GraphPad, La Jolla, CA). For all proliferation and cytokine analyses, unpaired *t* test with Welch’s correction was used to assess for multiple comparisons. For quantification of F-actin deposits, one-way ANOVA with Tukey’s multiple comparisons test was used. Significance was defined at a two-sided alpha level of 0.05.

## Results

### Crosstalk occurs between osteoblasts and breast cancer cells in the tumor microenvironment in vivo

**Fig. 1:**
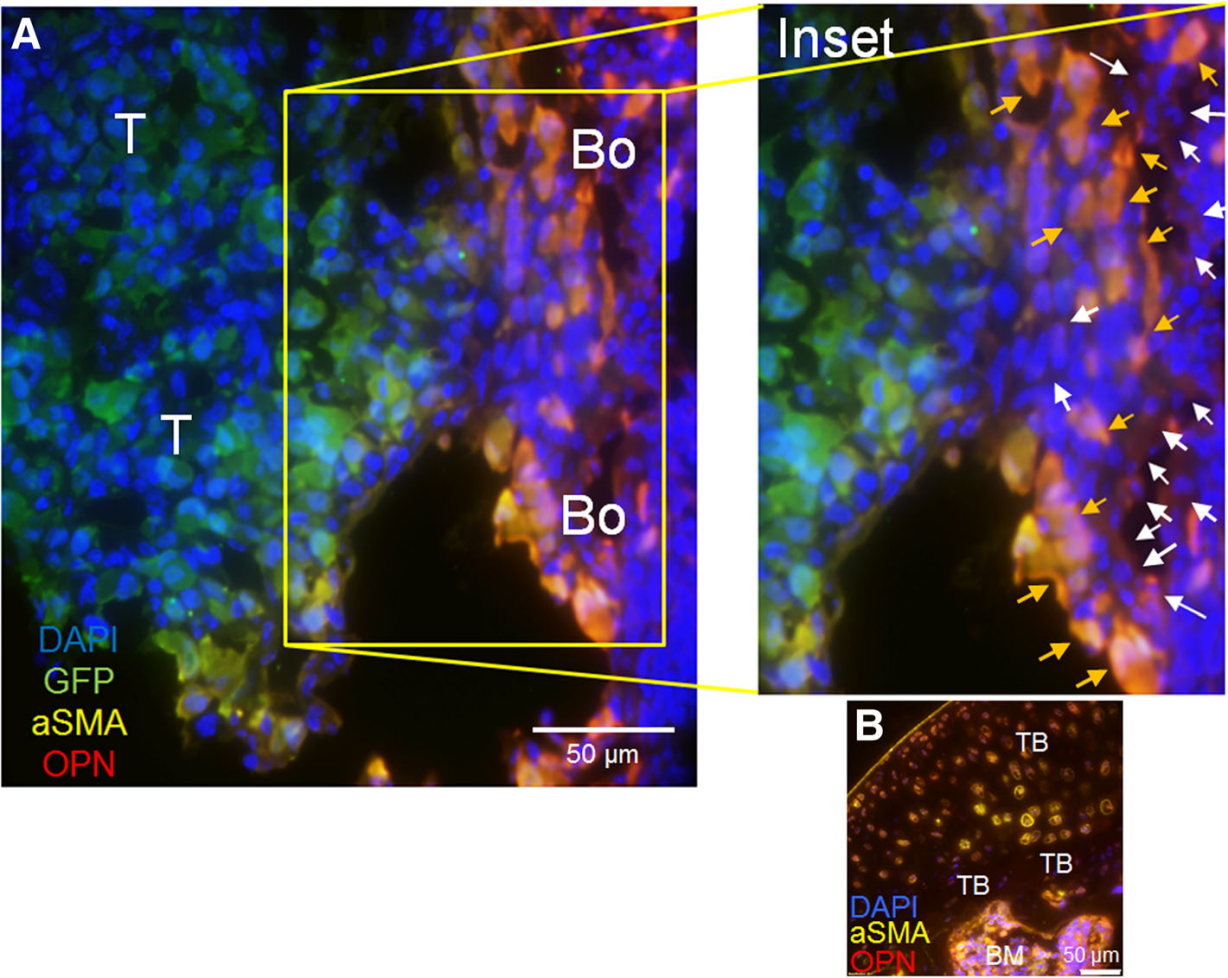
Osteoblast subpopulations in the tumor microenvironment in vivo. **a** Athymic nude mice were injected via intratibial injection with an admix of MDA-MB-231GFP/Luc2 human breast cancer cells plus osteoblasts or **b** PBS. Eight weeks later, mice were euthanized and their tibia harvested. Sections were stained for alpha-smooth muscle actin, osteopontin, green fluorescent protein, and DAPI via immunofluorescence. Inset: Orange arrows show alpha-smooth muscle actin-positive, osteopontin-positive osteoblasts. White arrows show alpha-smooth muscle actin low, osteopontin positive "educated" osteoblasts. **a** T, tumor; Bo, bone. **b** Non-cancer bearing bone (control). TB, trabecular bone; BM, bone marrow

Recruitment of stromal cells from normal tissue has been established as a prerequisite for tumor invasion and metastasis [55, 56]. There is mounting evidence to suggest that bone metastatic cancer cells act on osteoblasts in the tumor microenvironment to alter osteoblast production of proteins [32, 33, 57, 58]. Our previous results suggested that osteoblasts are significantly altered in the presence of breast cancer cells to increase osteoblast production of IL-6, IL-8, VEGF, MCP-1, and GRO-alpha [32, 33] in late-stage disease. These cytokines facilitate breast cancer cell colonization in the bone microenvironment [32, 33]. To assay for additional ways bone metastatic breast cancer cells may alter osteoblast properties in bone, we injected athymic nude mice via intratibial injection with an admix of MDA-MB-231GFP/Luc2 human breast cancer cells plus MC3T3-E1 murine osteoblasts. PBS was injected into the contralateral tibia as a control. Eight weeks later, the mice were euthanized and their tibia harvested and sectioned. To identify osteoblasts in the tumor microenvironment, we stained the sections using immunofluorescence for osteopontin (OPN), a bone turnover marker [59], and alpha-smooth muscle actin (aSMA), a marker for cells of the osteogenic lineage [60]. We also stained the sections for DAPI (nuclear stain) and GFP to identify GFP-expressing human breast cancer cells (Fig. 1). Unexpectedly, we saw two distinct populations of osteoblasts based on protein marker expression in cancer-bearing bones: (1) orange arrows point to osteoblasts both OPN-positive (red) and aSMA-positive (yellow)—combined colors result in osteoblasts orange in color; (2) white arrows point to osteoblasts OPN-positive (red), but aSMA low (yellow)—combined colors result in osteoblasts red in color (Fig. 1a, inset). The two populations of osteoblasts are adjacent to GFP-expressing breast cancer cells (green) (Fig. 1). This is in contrast to non-cancer-bearing bones, which exhibited only OPN-positive and aSMA-positive osteogenic populations (Fig. 1b). Although our evidence (Fig. 1a) suggests a larger population of OPN-positive and aSMA-positive osteoblasts adjacent to tumor cells, we expect this does not fully represent the three-dimensional spatial distributions of the two different osteoblast populations in the tumor niche in vivo. These results suggest that there are two distinct populations of osteoblasts in the tumor niche in vivo as defined by protein markers compared to non-cancer-bearing bone control.

We considered the possibility that the osteoblast subpopulations that we found were a result of injecting exogenous MC3T3-E1 cells into mouse bone where endogenous mouse osteoblasts would be present. In order to distinguish our injected mouse MC3T3-E1 cells from the endogenous mouse osteoblasts, we exploited the knowledge that homozygous Nu/Nu mice have a spontaneous mutation in the Forkhead Box N1 (FOXN1) gene (resulting in hairlessness and athymia), and thus are deficient for FOXN1 [61–63]. First, we tested if MC3T3-E1 cells express the FOXN1 protein by western blot and immunocytochemistry (Additional file 1: Figure S1). FOXN1 protein was expressed in MC3T3-E1 cells as observed by both western blot (Additional file 1: Figure S1A) and immunofluorescence (Additional file 1: Figure S1B). Next, we stained the tibia of mice injected with an admix of MDA-MB-231GFP/Luc2 human breast cancer cells plus MC3T3-E1 murine osteoblasts using antibodies for FOXN1, to show the population of injected MC3T3-E1 cells, and RUNX2 a unique marker of osteoblasts [64]. RUNX2 is a protein essential for the development of the osteoblast phenotype; thus, both endogenous mouse osteoblast cells as well as injected MC3T3-E1 cells will express RUNX2 [65–67]. In the examples shown, FOXN1 (yellow, arrows), representing the injected MC3T3-E1 cells, is observed interspersed in a random fashion throughout the total osteoblast population in the trabecular bone which stained positive for RUNX2 (red) (Additional file 1: Figure S1C). The integration of injected MC3T3-E1 cells into the trabecular bone, especially as evident in the example shown from tibia 1, suggests that the injected MC3T3-E1 cells and native endogenous mouse osteoblasts are functioning as one unified population in vivo. Therefore, these results suggest that the osteoblast subpopulations that we observed in vivo were not a result of injecting exogenous MC3T3-E1 cells into the mouse bone.

### Osteoblasts are “educated” by metastatic breast cancer cells

**Fig. 2:**
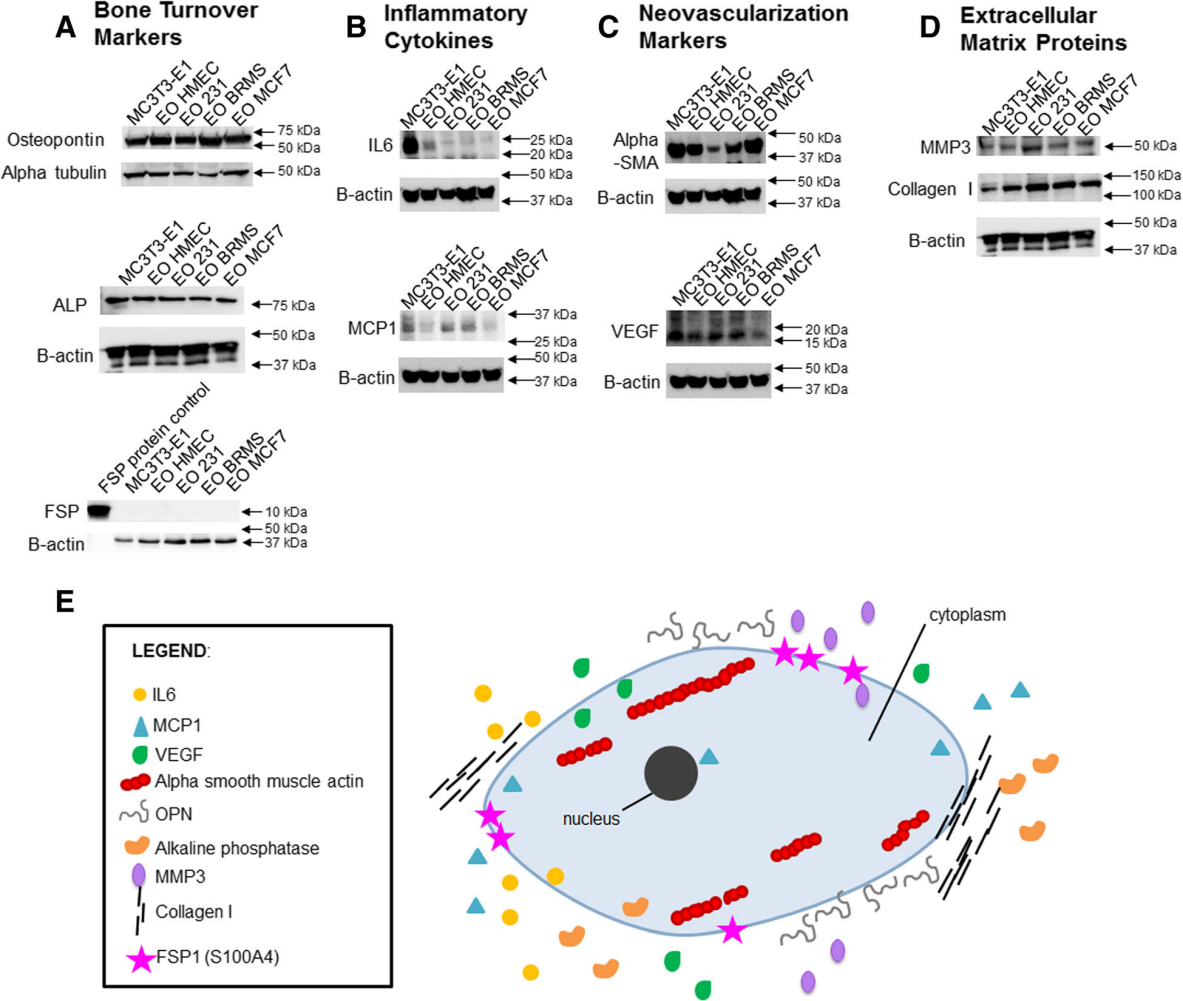
EO cells express different markers than normal osteoblasts. MC3T3-E1 cells were plated at 1 × 10^5^ cells/cm^2^ in 35 × 10 mm dishes. Twenty-four hours later, the media were replaced with differentiation medium, and cells grown to late differentiation (20 days). Differentiation medium was exchanged every third day. EO cells were plated at 1 × 10^5^ cells/cm^2^ and grown in three parts 1.5× differentiation medium plus one part either MDA-MB-231, MDA-MB-231BRMS, or MCF-7 breast cancer-conditioned medium or hTERT-HME1 mammary epithelial cell-conditioned medium. Media were changed every second day. To collect cell lysates, growth or differentiation media were removed, and cells washed with cold PBS, then lysates removed using ice-cold RIPA buffer. EO and MC3T3-E1 lysates were quantitated, then subjected to western blotting for proteins associated with **a** bone turnover (osteopontin (OPN), alkaline phosphatase (ALP), and fibroblast-specific protein (FSP)), **b** inflammatory cytokines (IL-6 and MCP-1), **c** neovascularization (alpha-smooth muscle actin (alpha-SMA) and VEGF) and **d** extracellular matrix (MMP3 and type I collagen). EO variants examined include EO cells made with hTERT-HME human mammary epithelial (EO HMEC), MDA-MB-231 human metastatic breast cancer (EO 231), MDA-MB-231BRMS human breast cancer metastasis-suppressed (EO BRMS), or MCF-7 human estrogen receptor-positive breast cancer (EO MCF7)-conditioned medium. Three biological replicates were carried out per condition, per time, and the experiment is repeated twice. Shown are representative results. **e** A “EO” can be defined as an osteoblast-like cell with altered expression of four defining characteristics which distinguish it from a normal osteoblast: bone turnover markers (OPN, ALP, and FSP), inflammatory cytokines (IL-6 and MCP-1), neovascularization (alpha-SMA and VEGF), and extracellular matrix markers (MMP3 and collagen I)

In order to replicate our in vivo results in vitro, we used MC3T3-E1 cells, which are pre-osteoblasts capable of differentiation to states of matrix mineralization in vitro [40]. We conditioned early (10 days) or late (20 days) differentiated osteoblasts with either (a) hTERT-HME human mammary epithelial CM (negative control), (b) MDA-MB-231 triple-negative breast cancer CM, (c) MDA-MB-231BRMS metastasis-suppressed breast cancer CM, or (d) MCF-7 ER+ luminal breast cancer CM over a period of 21 days [68]. Differentiated MC3T3-E1 osteoblasts treated with vehicle media were used as additional controls. We examined for alterations in the expression of proteins associated with (1) bone turnover (osteopontin, alkaline phosphatase, fibroblast-specific protein), (2) inflammatory cytokines (IL-6, MCP-1), (3) neovascularization (alpha-smooth muscle actin, VEGF), and (4) extracellular matrix remodeling (matrix metalloproteinase 3, collagen type I) (Fig. 2). These proteins were chosen due to their association with osteoblasts and bone matrix remodeling [39, 69–72], or osteoblasts and bone metastatic cancer [32, 33, 73]. We observed the largest differences between conditioned and vehicle-treated osteoblast protein expression in late (20 days) differentiated osteoblasts, which are shown in Fig. 2. Compared to vehicle-treated osteoblasts, osteoblasts “educated” with the conditioned medium of hTERT-HME1 cells (EO-HMEC) or MDA-MB-231, MDA-MB-231BRMS, or MCF-7 breast cancer cells (EO-231, EO-BRMS, and EO-MCF7, respectively) exhibited minimal to no change in the expression of osteopontin (Fig. 2a). Minimal to no change was observed in the EO cell expression of alkaline phosphatase when compared to MC3T3-E1 cells, and no fibroblast-specific protein expression was detected in MC3T3-E1 or EO cells (Fig. 2a). However, compared to vehicle-treated osteoblasts, EO-231, EO-BRMS, and EO-MCF7 cells exhibited a reduction in IL-6 protein expression, where very little IL-6 expression was found in these cells via western blot (Fig. 2b). Osteoblasts “educated” with the conditioned medium of hTERT-HME1 cells (EO-HMEC) also exhibited a reduction in IL-6 expression compared to vehicle-treated MC3T3-E1 cells (Fig. 2b). However, at least three times the amount of IL-6 protein was expressed in EO-HMEC cells than EO-231, EO-BRMS, and EO-MCF7 cells (Fig. 2b).

A reduction in protein expression was also observed in EO-231 and EO-BRMS expression of alpha-SMA when compared to vehicle-treated osteoblasts. Minimal to no change in alpha-SMA expression was observed in EO-HMEC or EO-MCF7 cells (Fig. 2c). There is a ~ 50% reduction in the inflammatory cytokine MCP-1 protein expression in EO-MCF7 cells when compared to vehicle-treated osteoblasts. Little to no change was seen in MCP-1 expression of EO-HMEC, EO-231, or EO-BRMS cells (Fig. 2b). Furthermore, a reduction in VEGF protein expression was observed in EO-MCF7 cells when compared to vehicle-treated osteoblasts. Little to no change was seen in VEGF expression of EO-HMEC, EO-231, or EO-BRMS cells (Fig. 2c).

By contrast, collagen I expression was upregulated in EO-231, EO-BRMS, and EO-MCF7 cells. A small increase in collagen expression was observed in EO-HMEC cells compared with vehicle-treated osteoblasts (Fig. 2d). Furthermore, MMP-3 expression was upregulated in EO-231 cells when compared to vehicle-treated osteoblasts. Small increases in MMP3 expression were observed in EO-HMEC, EO-BRMS, and EO-MCF7 cells when compared to vehicle-treated osteoblasts (Fig. 2d). These results suggest that when differentiated osteoblasts are in the presence of BCCM for a prolonged period of time (chronic exposure as opposed to acute), the osteoblasts are “educated” to produce proteins in different concentrations than vehicle-treated osteoblasts. These results additionally suggest that alterations in osteoblast protein expression are correlated with the type of breast cancer cell treatment, where osteoblasts treated with triple-negative BCCM exhibited a different protein expression profile than osteoblasts treated with ER+ luminal BCCM. Combined, reductions in cancer cell CM-educated osteoblasts were seen in the expression of IL-6 and alpha-SMA, whereas increases in educated osteoblast protein expression were observed with collagen type I. While differentiated osteoblast treatment with human mammary epithelial cell CM did elicit some alterations in osteoblast protein expression, these changes were minimal in comparison with alterations observed with treatment of breast cancer cell variant CM.

We define an “educated osteoblast” (EO) by characteristics outlined in Fig. 2e, which distinguish it from an “uneducated” osteoblast. These features include expression of bone turnover markers osteopontin and alkaline phosphatase, reduced expression of inflammatory cytokines (IL-6 and MCP-1), alterations in neovascularization markers alpha-SMA and VEGF, and increased expression of markers associated with extracellular matrix remodeling (collagen type I and MMP3), which are correlated with estrogen receptor status of the cancer cell variant CM treatment (Fig. 2a–d). In the following sections, we provide additional in vivo evidence that expression of these factors are uniquely altered only in tumors formed in the bone from admixes including EO cells when compared to “uneducated” osteoblasts or cancer cells injected alone, thereby illustrating the involvement of EOs as important modulators of metastatic progression in the bone.

### Marker expression in intratibial tumors harboring EO cells

To determine the relationship between EO cells, bone metastatic breast cancer cells, and the bone metastatic tumor microenvironment, we utilized a mouse model of intratibial injection which recapitulates the bone microenvironment in vivo during established metastatic disease [74]. In addition, since we observed the largest changes in protein expression when late-differentiated (20 days) osteoblasts were treated with human MDA-MB-231 metastatic breast cancer-conditioned medium (Fig. 2), we proceeded with the use of these cells for in vivo studies. MDA-MB-231 cells are a human triple-negative breast cancer cell line that frequently metastasize to and colonize the bone [44, 74–76]. MDA-MB-231GFP/Luc2 breast cancer cells were admixed with either EO-231 cells or vehicle-treated osteoblasts prior to intratibial injection. MDA-MB-231GFP/Luc2 cells inoculated alone were used as a control. Tumor growth was monitored using bioluminescence imaging.

#### Cortical and trabecular bone (endosteal niche)

**Fig. 3:**
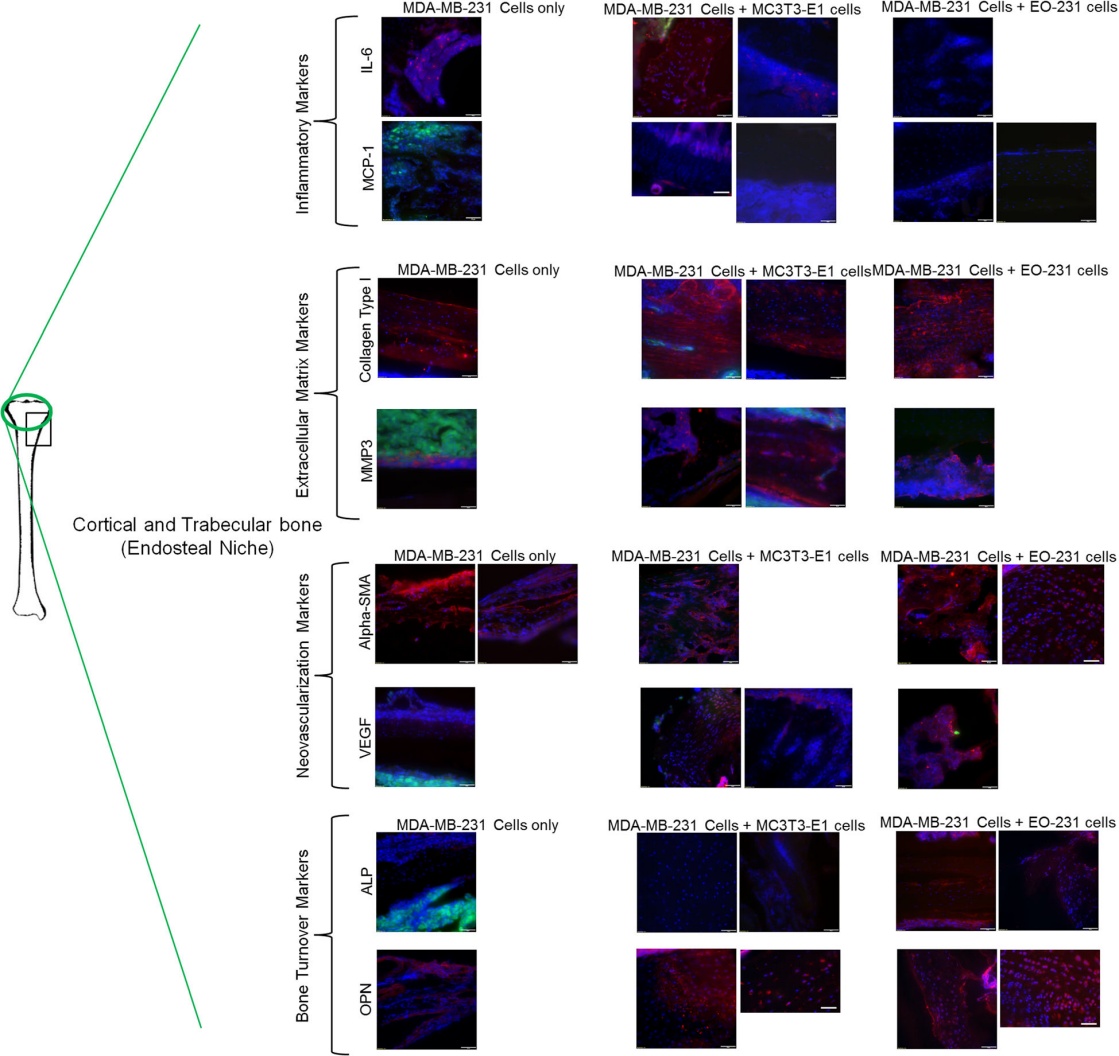
Unique protein expression occurs with EO cell presence in the endosteal niche of tumor-bearing bones. Athymic nude mice were injected via intratibial injection with an admix of MDA-MB-231GFP/Luc2 human breast cancer cells plus either EO-231 cells or MC3T3-E1 osteoblasts, or MDA-MB-231GFP/Luc2 cells alone. Eight weeks later, mice were euthanized and their tibia harvested. Tibia sections from athymic mice were prepared as described in the “Materials and methods” section. Sections were stained for osteopontin, alkaline phosphatase, VEGF, alpha-smooth muscle actin, MMP3, collagen type I, MCP-1, IL-6, and green fluorescent protein via immunofluorescence. The cortical and trabecular bone microenvironment was examined via fluorescent microscopy. As shown on the tibia at the left, the black box represents the positioning of the tumor in the examples shown, whereas the green circle represents the locations in the bone where the images were taken. At least three independent, serial sections were stained per bone and three bones examined per condition. Shown are representative images. Scale bar = 50 μm

To determine how the presence of EO cells in the bone alters the tumor microenvironment, we stained the tibia sections for markers corresponding to the four groups tested in Fig. 2: osteopontin and alkaline phosphatase as bone turnover markers, IL-6 and MCP-1 as inflammatory cytokines, alpha-SMA and VEGF as neovascularization markers, and type I collagen and MMP3 as extracellular matrix proteins. FSP was not tested due to the lack of expression in both vehicle-treated osteoblasts and EO cells (Fig. 2a). We utilized the adaptive threshold and count and measure the function in Olympus cellSens software to determine the percent tissue stained for a given protein, as distinguished per fluorophore, in each section. We compared three different sites for protein expression within tumor-bearing bone: (a) the endosteal niche including the cortical and trabecular bone (where osteoblasts are predominantly located), (b) the hematopoietic niche including the bone marrow, and (c) the tumor itself. Within the cortical and trabecular bone, IL-6 was expressed in ~ 37% of cells in the trabecular bone of tumor-bearing mice injected with MDA-MB-231GFP/Luc2 cells alone (Fig. 3). IL-6 was only expressed in ~ 2% of cells in the trabecular bone of tumor-bearing mice injected with MDA-MB-231GFP/Luc2 cells plus EO-231 cells (Fig. 3), corresponding to in vitro results (Fig. 2). On the other hand, MCP-1 expression was completely absent in the trabecular bone near the tumor in mice inoculated with MDA-MB-231GFP/Luc2 cells alone (0% of cells), while some MCP-1 expression was observed in the trabecular and cortical bones of tumor-bearing mice injected MDA-MB-231GFP/Luc2 cells plus either MC3T3-E1 cells (~ 16% of cells) or EO-231 cells (~ 1% of cells) (Fig. 3).

We next examined the bones of tumor-bearing mice for markers associated with the extracellular matrix. Type I collagen expression was expressed in ~ 55% of cells in the trabecular and cortical bone of tumor-bearing mice injected with MDA-MB-231GFP/Luc2 cells plus EO-231 cells, ~ 64% of cells in tumor-bearing mice injected with MDA-MB-231GFP/Luc2 cells plus MC3T3-E1 cells, or ~ 55% of cells in tumor-bearing mice injected with MDA-MB-231GFP/Luc2 cells injected alone (Fig. 3). Interestingly, in the trabecular or cortical bone of tumor-bearing mice injected with MDA-MB-231GFP/Luc2 cells injected alone, MMP3 expression was observed near or adjacent to GFP breast cancer cells (~ 17% of cells) (Fig. 3). By contrast, MMP expression in the trabecular or cortical bone of tumor-bearing mice injected with MDA-MB-231GFP/Luc2 cells plus either EO-231 cells (~ 44% of cells) or MC3T3-E1 cells (~ 41% of cells) was observed in the trabecular bone away from breast cancer cells (Fig. 3).

We next examined the trabecular and cortical bone for the neo-vascularization markers alpha-SMA and VEGF. Alpha-SMA was expressed in both the cortical and trabecular bones of tumor-bearing mice in all conditions tested: MDA-MB-231GFP/Luc2 cells plus MC3T3-E1 cells (~ 63% of cells), MDA-MB-231GFP/Luc2 cells plus EO-231 cells (~ 71% of cells), or MDA-MB-231GFP/Luc2 cells injected alone (~ 50% of cells) (Fig. 3). We observed the expression of moderate amounts of VEGF in the trabecular bone of tumor-bearing mice injected with either MDA-MB-231GFP/Luc2cells plus MC3T3-E1 cells (~ 25% of cells) or MDA-MB-231GFP/Luc2 cells plus EO-231 cells (~ 33% of cells) (Fig. 3). VEGF expression in the trabecular bone was absent in tumor-bearing mice injected with MDA-MB-231GFP/Luc2 cells alone (0% of cells) (Fig. 3).

Finally, we examined the trabecular and cortical bone for bone turnover markers including osteopontin and alkaline phosphatase. Osteopontin was expressed in ~ 47% of cells in the bones of tumor-bearing mice injected with MDA-MB-231GFP/Luc2 cells plus EO-231 cells and expressed in ~ 80% of cells in the bones of tumor-bearing mice injected with MDA-MB-231GFP/Luc2 cells plus MC3T3-E1 cells (Fig. 3). By contrast, osteopontin was expressed in ~ 39% of cells in the bones of tumor-bearing mice injected with MDA-MB-231GFP/Luc2 cells alone, when compared to the bones of tumor-bearing mice injected with either MDA-MB-231GFP/Luc2 cells plus EO-231 cells or MDA-MB-231GFP/Luc2 cells plus MC3T3-E1 cells (Fig. 3). We observed the expression of alkaline phosphatase in the trabecular and cortical bone of tumor-bearing mice injected with MDA-MB-231GFP/Luc2 cells plus EO-231 cells (~ 29% of cells) (Fig. 3). By contrast, the expression of alkaline phosphatase was absent in the trabecular and cortical bone of tumor-bearing mice injected with either MDA-MB-231GFP/Luc2 cells plus MC3T3-E1 cells or MDA-MB-231GFP/Luc2 cells injected alone (0% of cells) (Fig. 3).

Combined, similar expression of proteins examined, except for alkaline phosphatase, were observed in the cortical and trabecular bone of tumor-bearing mice injected with MDA-MB-231GFP/Luc2 cells plus MC3T3-E1 cells (Fig. 3). Interestingly, we observed the most change (reduction) in the proteins expressed (ALP, VEGF, MCP-1; all absent) in the cortical and trabecular bone of tumor-bearing mice injected with MDA-MB-231GFP/Luc2 cells alone (Fig. 3). IL-6 expression, on the other hand, was increased approximately 20-fold in the cortical and trabecular bone of tumor-bearing mice injected with MDA-MB-231GFP/Luc2 cells alone when compared to mice injected with MDA-MB-231GFP/Luc2 cells plus either EO-231 or MC3T3-E1 cells (Fig. 3).

When assayed alone in vitro, MC3T3-E1 osteoblasts expressed alpha-SMA, MCP-1, MMP-3, collagen type I, IL-6, VEGF, alkaline phosphatase, and osteopontin in moderate to high amounts (Additional file 2: Figure S2). MDA-MB-231 cells expressed no to negligible amounts of VEGF, MCP-1, MMP-3, collagen type I, and IL-6, indicating that mouse-specific antibodies were not cross-reactive with human-specific epithelium (Additional file 3: Figure S3). MDA-MB-231 cells did, however, express alpha-SMA, alkaline phosphatase, and osteopontin (Additional file 3: Figure S3), which corroborated with the manufacturer’s description. The secondary antibodies donkey anti-goat 488, goat anti-rabbit 488, and donkey anti-mouse 594 were neither reactive against mouse nor human cells (Additional file 4: Figure S4).

#### Bone marrow (hematopoietic niche)

**Fig. 4:**
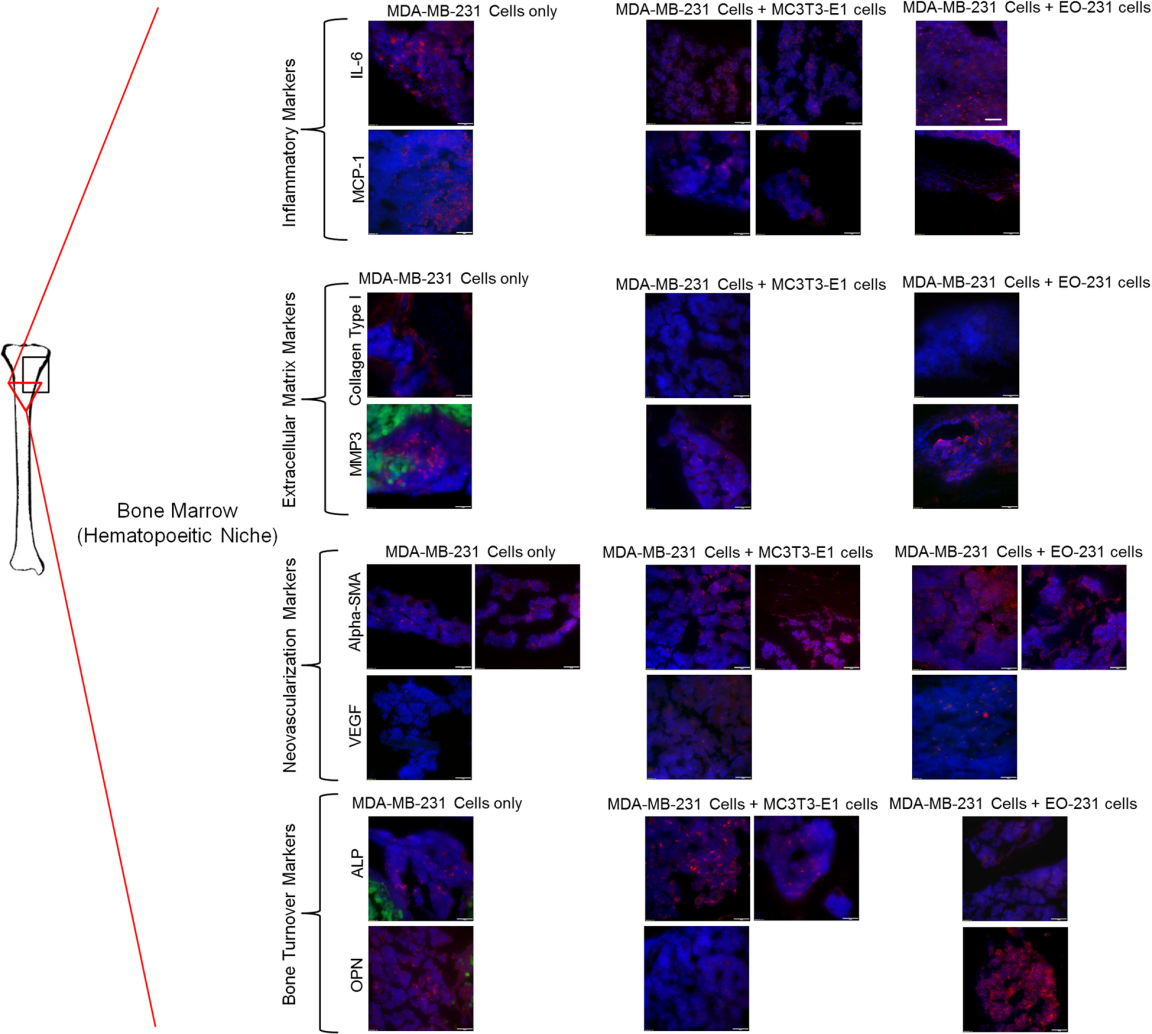
Unique protein expression occurs with EO cell presence in the hematopoietic niche of tumor-bearing bones. Athymic nude mice were injected via intratibial injection with an admix of MDA-MB-231GFP/Luc2 human breast cancer cells plus either EO-231 cells or MC3T3-E1 osteoblasts, or MDA-MB-231GFP/Luc2 cells alone. Eight weeks later, mice were euthanized and their tibia harvested. The tibia sections from athymic mice were prepared as described in the “Materials and methods” section. The sections were stained for osteopontin, alkaline phosphatase, VEGF, alpha-smooth muscle actin, MMP3, collagen type I, MCP-1, IL-6, and green fluorescent protein via immunofluorescence. The bone marrow microenvironment was examined via fluorescent microscopy. As shown on the tibia at the left, the black box represents the positioning of the tumor in the examples shown, whereas the red triangle represents the locations in the bone where the images were taken. At least three independent, serial sections were stained per bone and three bones examined per condition. Shown are representative images. Scale bar = 50 μm

We next examined for the changes in the hematopoietic niche including the bone marrow. IL-6 was expressed in similar amounts in the bone marrow of all conditions examined: tumor-bearing mice injected with either MDA-MB-231GFP/Luc2 cells plus EO-231 cells (~ 27% of cells), MDA-MB-231GFP/Luc2 cells plus MC3T3-E1 cells (~ 40% of cells), or MDA-MB-231GFP/Luc2 cells alone (~ 27% of cells) (Fig. 4). MCP-1 was expressed in similar amounts in the bone marrow of all conditions examined: tumor-bearing mice injected with either MDA-MB-231GFP/Luc2 cells plus EO-231 cells (~ 35% of cells), MDA-MB-231GFP/Luc2 cells plus MC3T3-E1 cells (~ 39% of cells), or MDA-MB-231GFP/Luc2 cells alone (~ 32% of cells) (Fig. 4).

We next examined the bones of tumor-bearing mice for markers associated with the extracellular matrix. Type I collagen was present in the bone marrow of tumor-bearing mice injected with MDA-MB-231GFP/Luc2 cells alone (~ 37% of cells) (Fig. 4). By contrast, no type I collagen (0% of cells) was detected in the bone marrow of tumor-bearing mice injected with MDA-MB-231GFP/Luc2 cells plus either EO-231 or MC3T3-E1 cells (Fig. 4). Interestingly, MMP3 was expressed in ~ 33% of cells in the bone marrow of tumor-bearing mice injected with MDA-MB-231GFP/Luc2 cells alone (Fig. 4). By contrast, MMP3 was expressed in ~ 25% of cells in the bone marrow of tumor-bearing mice injected with MDA-MB-231GFP/Luc2 cells plus MC3T3-E1 cells and in ~ 12% of cells in the bone marrow of tumor-bearing mice injected with MDA-MB-231GFP/Luc2 cells plus EO-231 cells (Fig. 4).

We next examined the bone marrow for the neo-vascularization markers alpha-SMA and VEGF. Alpha-SMA was expressed in the bone marrow of tumor-bearing mice in all conditions tested: MDA-MB-231GFP/Luc2 cells plus MC3T3-E1 cells (~ 57% of cells), MDA-MB-231GFP/Luc2 cells plus EO-231 cells (~ 54% of cells), or MDA-MB-231GFP/Luc2 cells injected alone (~ 42% of cells) (Fig. 4). Similar to the trabecular and cortical bone, we observed the expression of VEGF in the bone marrow of tumor-bearing mice injected with either MDA-MB-231GFP/Luc2 cells plus MC3T3-E1 cells (~ 41% of cells) or MDA-MB-231GFP/Luc2 cells plus EO-231 cells (~ 23% of cells) (Fig. 4). VEGF expression in the bone marrow was absent in tumor-bearing mice injected with MDA-MB-231GFP/Luc2 cells alone (0% of cells) (Fig. 4).

Finally, osteopontin was expressed in ~ 45% of cells in the bone marrow of bones of tumor-bearing mice injected with MDA-MB-231GFP/Luc2 cells plus EO-231 cells, but was expressed in only ~ 17% of cells in the bone marrow of tumor-bearing mice injected with MDA-MB-231GFP/Luc2 cells alone (Fig. 4). By contrast, osteopontin expression was completely absent in the bone marrow of bones of tumor-bearing mice injected with MDA-MB-231GFP/Luc2 cells plus MC3T3-E1 cells (0% of cells) (Fig. 4). We observed the expression of alkaline phosphatase in ~ 15% of cells in the bone marrow of mice injected with MDA-MB-231GFP/Luc2 cells plus EO-231 cells, ~ 27% of cells in the bone marrow of mice injected with MDA-MB-231GFP/Luc2 cells plus MC3T3-E1 cells, and ~ 13.5% of cells in the bone marrow of mice injected with MDA-MB-231GFP/Luc2 cells alone (Fig. 4).

When compared to protein expression in the endosteal niche, the hematopoietic niche exhibited reduced amounts of ECM remodeling proteins in the tumor-bearing bones of mice injected with MDA-MB-231GFP/Luc2 cells plus either MC3T3-E1 cells or EO-231 cells (Fig. 4). Mice injected with MDA-MB-231GFP/Luc2 cells alone, however, expressed more ECM remodeling proteins (especially type I collagen) in the bone marrow (Fig. 4). Minimal to no change was seen in the expression of inflammatory cytokines present in the bone marrow of tumor-bearing mice of all conditions tested: MDA-MB-231GFP/Luc2 cells plus either MC3T3-E1 cells or EO-231 cells or MDA-MB-231GFP/Luc2 cells alone (Fig. 4). Interestingly, while alpha-SMA was present in all conditions tested and VEGF expression was high in the bone marrow of mice injected with MDA-MB-231GFP/Luc2 cells plus either MC3T3-E1 cells or EO-231 cells, VEGF was completely absent in the bone marrow of mice injected with MDA-MB-231GFP/Luc2 cells alone (Fig. 4). In addition, osteopontin expression was completely absent in the bone marrow of tumor-bearing mice injected with MDA-MB-231GFP/Luc2 cells plus MC3T3-E1 cells when compared to mice injected with either MDA-MB-231GFP/Luc2 cells plus EO-231 cells or MDA-MB-231GFP/Luc2 cells alone (Fig. 4). We observed an increase (~ 50%) in the expression of alkaline phosphatase in the bone marrow of tumor-bearing mice injected with MDA-MB-231GFP/Luc2 cells plus MC3T3-E1 cells when compared to mice injected with either MDA-MB-231GFP/Luc2 cells plus EO-231 cells or MDA-MB-231GFP/Luc2 cells alone (Fig. 4).

#### Tumor

Lastly, we examined the tumor itself for the changes in protein expression in vivo as a result of the presence of EO cells. Tumors were present via the expression of GFP in all the tibia examined (Additional file 5: Figure S5). We observed the expression of IL-6 in the tumors of mice injected with MDA-MB-231GFP/Luc2 cells plus EO-231 cells (~ 17%) (Additional file 6: Figure S6). IL-6 expression was absent (0% of cells) in the tumors of both mice injected with MDA-MB-231GFP/Luc2 cells plus MC3T3-E1 cells or MDA-MB-231GFP/Luc2 cells alone (Additional file 6: Figure S6). On the other hand, a small amount of MCP-1 was expressed in the tumors of mice injected with MDA-MB-231GFP/Luc2 cells plus MC3T3-E1 cells (~ 5% of cells) (Additional file 6: Figure S6). By contrast, no MCP-1 was expressed in the tumors of mice injected with either MDA-MB-231GFP/Luc2 cells plus EO-231 or MDA-MB-231GFP/Luc2 cells alone (Additional file 6: Figure S6).

Next, we examined the bones of tumor-bearing mice for markers associated with the extracellular matrix. We observed ~ 24% of cells to be positive for type I collagen expression in the tumors of mice injected with MDA-MB-231GFP/Luc2 cells plus MC3T3-E1 cells (Additional file 6: Figure S6). Similarly, ~ 24% of cells expressed type I collagen in the tumors of mice injected with MDA-MB-231GFP/Luc2 cells plus EO-231 cells or MDA-MB-231GFP/Luc2 cells alone (Additional file 6: Figure S6). Little to no MMP3 was observed in the tumor of mice injected with MDA-MB-231GFP/Luc2 cells plus either EO-231 (~ 17% of cells) or MC3T3-E1 cells (~ 5% of cells). Small to moderate amounts of MMP3 were observed in the tumors of mice injected with MDA-MB-231GFP/Luc2 cells alone (~ 33% of cells) (Additional file 6: Figure S6).

We examined the tumors of mice for the neo-vascularization markers alpha-SMA and VEGF. Alpha-SMA was expressed in all conditions tested: MDA-MB-231GFP/Luc2 cells plus MC3T3-E1 cells (~ 15% of cells), MDA-MB-231GFP/Luc2 cells plus EO-231 cells (~ 28% of cells), or MDA-MB-231GFP/Luc2 cells injected alone (~ 19% of cells) (Additional file 6: Figure S6). We observed the expression of VEGF in the tumors of mice injected with MDA-MB-231GFP/Luc2 cells alone (~ 17% of cells) (Additional file 6: Figure S6). VEGF expression was also detected in the tumors of mice injected with either MDA-MB-231GFP/Luc2 cells plus either MC3T3-E1 (~ 13% of cells) or EO-231 cells (~ 29% of cells) (Additional file 6: Figure S6).

Finally, we observed the expression of alkaline phosphatase within the stroma near tumor cells of tumor-bearing mice injected with MDA-MB-231GFP/Luc2 cells alone (~ 12%) (Additional file 6: Figure S6). No alkaline phosphatase expression was observed within the tumor of mice injected with MDA-MB-231GFP/Luc2 cells plus either MC3T3-E1 cells or EO-231 cells (0% of cells) (Additional file 6: Figure S6). Similarly, osteopontin was expressed within the stroma of the tumor in the tumor-bearing bones of mice injected with MDA-MB-231GFP/Luc2 cells alone (~ 15% of cells) (Additional file 6: Figure S6). Osteopontin was observed in the tumor-bearing bones of mice injected with MDA-MB-231GFP/Luc2 cells plus EO-231 cells (~ 35% of cells); however, no osteopontin expression was found in the tumor of mice injected with MDA-MB-231GFP/Luc2 cells plus MC3T3-E1 cells (0% of cells) (Additional file 6: Figure S6).

Overall, protein expression was reduced or completely absent in the tumors of mice injected with MDA-MB-231GFP/Luc2 cells plus EO-231 cells. The tumors of mice injected with MDA-MB-231GFP/Luc2 cells alone harbored the greatest amount of proteins tested when compared to mice injected with MDA-MB-231GFP/Luc2 cells plus either EO-231 or MC3T3-E1 cells (Additional file 6: Figure S6).

A summary of these results can be found in Table 1. Combined, these results suggest that there are differences in the proteins we examined that are expressed in tumor-bearing bone depending on the osteogenic composition of the bone-tumor microenvironment. In particular, the presence of EO cells in the tumor appears to reduce protein expression across all proteins examined (Additional file 6: Figure S6, Table 1). In addition, the presence of EO cells increased the expression of the bone turnover and osteoblast differentiation marker alkaline phosphatase in the cortical and trabecular bone of tumor-bearing mice (Fig. 3, Table 1). The presence of EO cells also suppressed ECM protein expression in the hematopoietic niche when compared to the presence of vehicle-treated MC3T3-E1 cells or MDA-MB-231GFP/Luc2 cells inoculated alone (Fig. 4, Table 1). These results imply that EO cells promote osteoblast differentiation while suppressing tumor progression and matrix remodeling.

**Table 1:** Expression of proteins in the cortical and trabecular bone, bone marrow, and tumor of mice inoculated via intratibial injection. Athymic nude mice were injected via intratibial injection with an admix of MDA-MB-231GFP/Luc2 human breast cancer cells plus either EO-231 cells or MC3T3-E1 osteoblasts, or MDA-MB-231GFP/Luc2 cells alone. Eight weeks later, mice were euthanized and their tibia harvested. Sections were stained for osteopontin, alkaline phosphatase, VEGF, alpha-smooth muscle actin, MMP3, collagen type I, MCP-1, IL-6, and green fluorescent protein via immunofluorescence. At least three independent sections were stained per bone, and three bones examined per condition. Protein expression is listed as a percentage of the total population of cells as quantified using Count and Measure per fluorophore in cellSens (Olympus)

Cell type injected	Bone turnover		Neovascularization		Inflammatory cytokines		Extracellular matrix remodeling	
	ALP (%)	OPN (%)	aSMA (%)	VEGF (%)	IL-6 (%)	MCP-1 (%)	Col-1 (%)	MMP3 (%)
Cortical and trabecular bone (endosteal niche)								
EO + 231	29	47	71	33	2	1	55	44 (away from BC)
OB + 231	0	80	63	25	55	16	64	41 (away from BC)
231 alone	0	39	50	0	37	0	55	17 (close to BC)
Bone marrow (hematopoietic niche)								
EO + 231	15	45	54	23	27	35	0	12
OB + 231	27	0	57	41	40	39	0	25
231 alone	13.5	17	42	0	27	32	37	33
Tumor itself								
EO + 231	0	35	28	29	17	0	24	17
OB + 231	0	0	15	13	0	5	24	5
231 alone	12	15	19	17	0	31	23.5	33

### EO cells in the bone of patients with bone metastatic breast cancer

In order to determine if EO cells were present in the bones of patients with metastatic breast cancer, we first sought to identify osteoblasts present in the tumor niche. Antibodies to human osteocalcin and RUNX2, both unique markers of osteoblasts [59, 64], were optimized using NHOst human osteoblasts (Additional file 7: Figure S7). RUNX2 is a protein essential for the development of the osteoblast phenotype [65–67]. Osteocalcin is an abundant bone matrix protein preferentially expressed by osteoblasts [77, 78]. Next, we obtained de-identified human bone samples from the femoral heads of patients undergoing total hip replacement or proximal femur replacement with breast cancer metastases to the bone. Human bone metastatic breast cancer patient samples exhibited isolated areas of cells that were positive for both RUNX2 and osteocalcin expression demonstrating the presence of osteoblasts among tumor cells (Additional file 8: Figure S8). The osteoblasts were located both adjacent to (Additional file 8: Figure S8A) and away from (Additional file 8: Figure S8B) tumor cells.

**Fig. 5:**
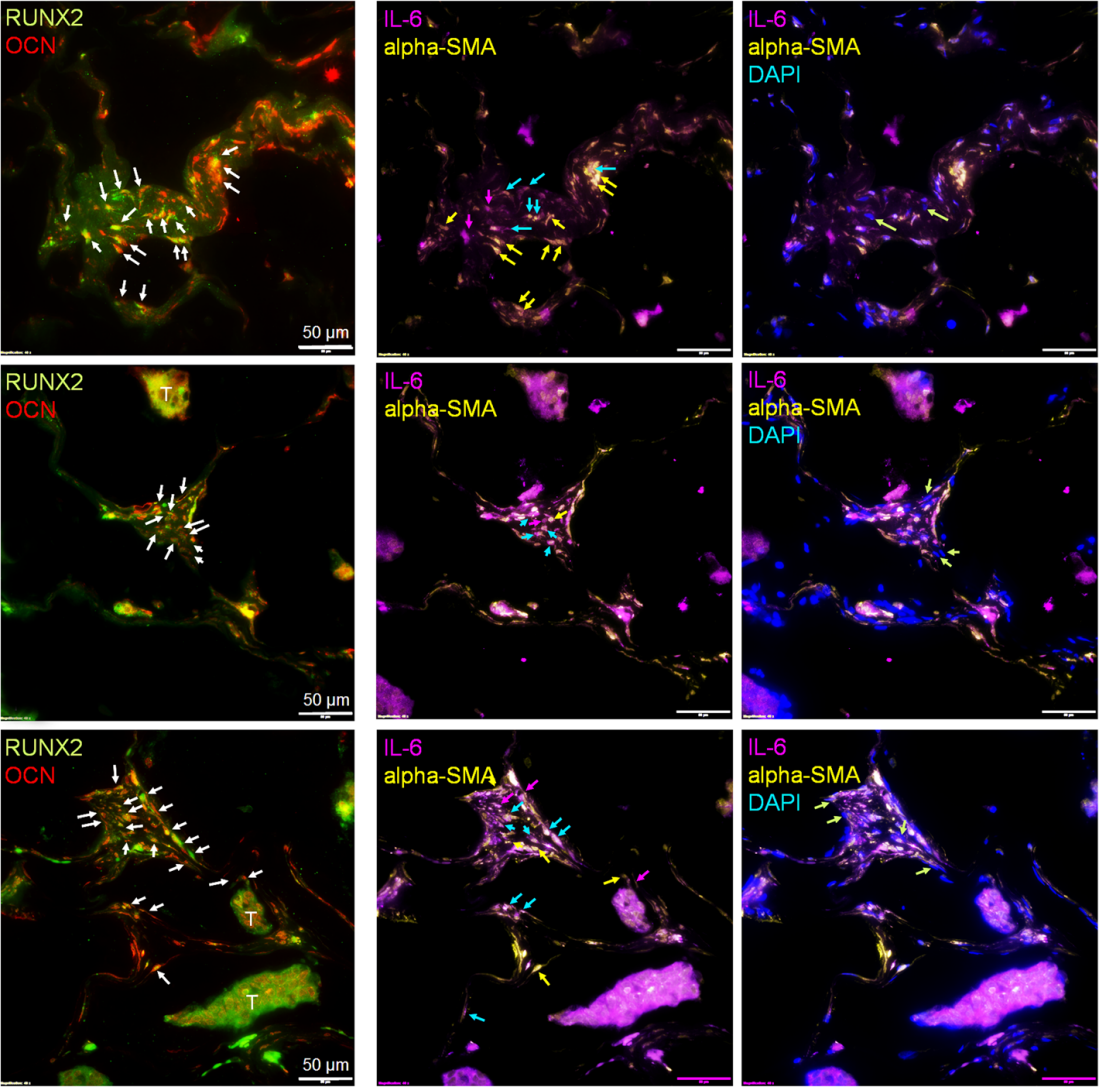
EOs are present in patient samples of bone metastatic breast cancer. Human patient samples of bone metastatic breast cancer were stained using multi-plex immunofluorescence for RUNX2 (green), osteocalcin (OCN, red), IL-6 (purple), and alpha-SMA (yellow). Left panel—osteoblast identification: white arrows show osteoblasts positive for both RUNX2 and OCN. Middle panel—“uneducated” and “educated” osteoblast identification: blue arrows show “uneducated” osteoblasts alpha-SMA and IL-6 positive; yellow arrows show “educated” osteoblasts alpha-SMA high, but IL-6 low; purple arrows show “educated” osteoblasts IL-6 high, but alpha-SMA low. Right panel—“educated” osteoblast identification: green arrows show “educated” osteoblasts both IL-6 and alpha-SMA low, DAPI positive. T, tumor; arrows, osteoblast. DAPI, nuclear stain. Scale bar = 50 μm

Next, to identify the presence of EO cells in human bone metastatic patient samples, we employed multi-plex immunofluorescent staining to examine the combined expression of RUNX2, osteocalcin, osteopontin, IL-6, and alpha-SMA cells. We define an osteoblast by its expression of RUNX2, osteocalcin, osteopontin, and IL-6 plus alpha-smooth muscle actin (Fig. 2, Additional file 2: Figure S2, Additional file 7: Figure S7, Additional file 8: Figure S8, and Additional file 9: Figure S9). We further define an EO cell by its expression of RUNX2, osteocalcin, and osteopontin but reduced expression of alpha-SMA and lack of expression of IL-6 (Figs. 1 and 2). Antibodies to human osteopontin, IL-6, and alpha-SMA were optimized using human NHOst osteoblasts (Additional file 9: Figure S9). Then, human bone samples from the femoral heads of patients undergoing total hip replacement or proximal femur replacement with breast cancer metastases to the bone were stained for RUNX2, OCN, IL-6, and alpha-SMA using multi-plex immunofluorescence (Fig. 5). Osteoblasts positive for both RUNX2 and OCN (Fig. 5, left panel; red plus green co-labeled cells, white arrows) were found throughout the bone samples from patients with bone metastases. Among those osteoblasts, approximately 40% were positive for both IL-6 and alpha-SMA (Fig. 5, middle panel; purple plus yellow = white cells, blue arrows). Osteoblasts expressing high levels of alpha-SMA but low levels of IL-6 were also present (~ 30%; Fig. 5, middle panel; yellow cells, yellow arrows), as well as a smaller number of osteoblasts that expressed high levels of IL-6 but low levels of alpha-SMA (~ 10%; Fig. 5, middle panel; purple cells, purple arrows). A small number of EO cells were identified among the RUNX2 and OCN osteoblast population as defined by their lack of expression of both IL-6 and alpha-SMA (~ 20%; Fig. 5, right panel; DAPI, green arrows). Thus, these results suggest that there are subpopulations of osteoblasts present in the bone of patients with bone metastases as defined by their expression of protein markers.

### EO cells differentiate and mineralize

Because we observed differences in the protein expression between osteoblast subpopulations both in vitro and in vivo, we next wanted to determine if there were other distinguishing characteristics between “uneducated” osteoblasts and EO cells. To determine if EO cells differentiate or mineralize, alkaline phosphatase and Von Kossa staining were used to define the bone turnover and mineralization, respectively [50–52]. MC3T3-E1 cells (control) were grown to late differentiation (20 days). EO-HMEC, EO-231, EO-BRMS, and EO-MCF7 cells were grown to confluence. Media were removed, then cells were fixed and stained for alkaline phosphatase expression or Von Kossa for deposition of calcium, indicating mineralization (Additional file 10: Figure S10). EO-HMEC, EO-231, and EO-MCF7 cells stained positive for alkaline phosphatase (Additional file 10: Figure S10A, arrows) at similar levels as 20-day differentiated MC3T3-E1 cells (control). Low to negligible levels of alkaline phosphatase expression were detected in EO-BRMS cells (Additional file 10: Figure S10A). Similar levels of Von Kossa staining were apparent in EO-HMEC, EO-231, EO-MCF7, and 20-day differentiated MC3T3-E1 cells (Additional file 10: Figure S10B, brown spots, arrows). Interestingly, low levels of Von Kossa staining were detected in EO-BRMS cells (Additional file 10: Figure S10B, brown spots, arrows). The amount of mineralization as detected by Von Kossa staining in EO-BRMS cells was considerably less than amounts observed in EO-HMEC, EO-231, EO-MCF7, and 20-day differentiated MC3T3-E1 cells. Therefore, EO-HMEC, EO-231, and EO-MCF7 cells differentiate and mineralize in a similar manner as 20-day differentiated MC3T3-E1 cells.

### EO cells have increased rates of proliferation compared to normal osteoblasts

We next compared EO proliferation to “uneducated” osteoblast proliferation in vitro. Proliferation was graphed in terms of the mean number of cells over the course of 10 days. We compared several EO “variants”: EO cells “educated” using MDA-MB-231 human triple-negative breast cancer conditioned media, EO cells “educated” using MDA-MB-231BRMS human breast cancer metastasis suppressor conditioned media, EO cells “educated” using MCF-7 ER+ human breast cancer conditioned media, and EO cells “educated” using hTERT-HME1 human epithelial cell conditioned media (control). Compared to vehicle-treated osteoblast proliferation, exposure to MDA-MB-231, MDA-MB-231BRMS, or MCF-7 conditioned medium elicited a statistically significant increase in osteoblast proliferation at all time points examined (Additional file 11: Figure S11). Interestingly, osteoblasts “educated” with MDA-MB-231BRMS conditioned media were the EO cells that proliferated the slowest, as opposed to EO-231 cells, which proliferated the fastest. We did also find an increase in osteoblast proliferation upon treatment with hTERT-HME1 conditioned medium (Additional file 11: Figure S11), suggesting that the increases observed in EO proliferation in vitro when compared to “uneducated” osteoblasts may not be cancer cell specific.

### F-actin organization is altered in EO cells

There has been evidence to suggest that co-culture with metastatic breast cancer cells or treatment with their conditioned media causes a reduction in osteoblast focal adhesion plaques, stress fiber formation, and F-actin organization [26]. Next, we sought to determine if we observed similar phenomena with EO cells as compared to “uneducated” osteoblasts. We utilized a phalloidin stain to examine F-actin organization in EO cells and “uneducated” osteoblasts. When compared to “uneducated” osteoblasts, we observed an increase in the number of F-actin clusters along the periphery of the cell in the EO cell variants produced using triple-negative breast cancer conditioned media: EO-231 and EO-BRMS (Additional file 12: Figure S12A, white arrows). There was a statistically significant increase in the number of the structures in EO-231 and EO-BRMS cells when compared to “uneducated” osteoblasts, which had negligible amounts of the structures (Additional file 12: Figure S12B). F-actin deposits were also observed in EO-MCF7 cells; however, these were small in number and comparable to that found on “uneducated” osteoblasts (Additional file 12: Figure S12A, green arrows; Additional file 12: Figure S12B). We observed a small number of F-actin deposits on EO-HMEC cells (control); however, these were comparable to numbers seen on “uneducated” osteoblasts (Additional file 12: Figure S12A-B).

We considered that the F-actin deposits we observed in EO-231 and EO-BRMS cells might be a result of cellular autophagy. Under conditions of stress, autophagy by cells is an adaptive response in order to promote cell survival. Upon internalization of the material targeted for degradation, autophagosomes within cells travel to the cytoplasm to fuse with a lysosome, forming an autolysosome that degrades its contents via lysosomal hydrolases [79]. To explore whether the F-actin deposits were in fact autophagosomes, we stained EO-231 and EO-BRMS cells (Additional file 13: Figure S13) with an antibody to light chain 3 (LC3), which examines for the expression of LC3, a marker of autophagosomes and a central protein involved in the autophagy pathway [80]. LC3 is a soluble protein that is ubiquitously expressed in tissue and is used as a marker of autophagy [81–83]. During autophagy, the cytosolic form of LC3 (LC3-I) is engulfed, cleaved, and conjugated with phosphatidylethanolamine [81, 82, 84]. LC3-phosphatidylethanolamine conjugate (LC3-II) is recruited to the autophagosomal membrane, serving as an indicator of autophagy [83, 85]. Immunofluorescent staining revealed that EO-231 and EO-BRMS elicited no evidence of the LC3 stain which was comparable to that of “uneducated” MC3T3-E1 osteoblasts, suggesting that the structures we observed were not that of autophagosomes (Additional file 13: Figure S13).

### EO cells decrease breast cancer cell proliferation in vitro

**Fig. 6:**
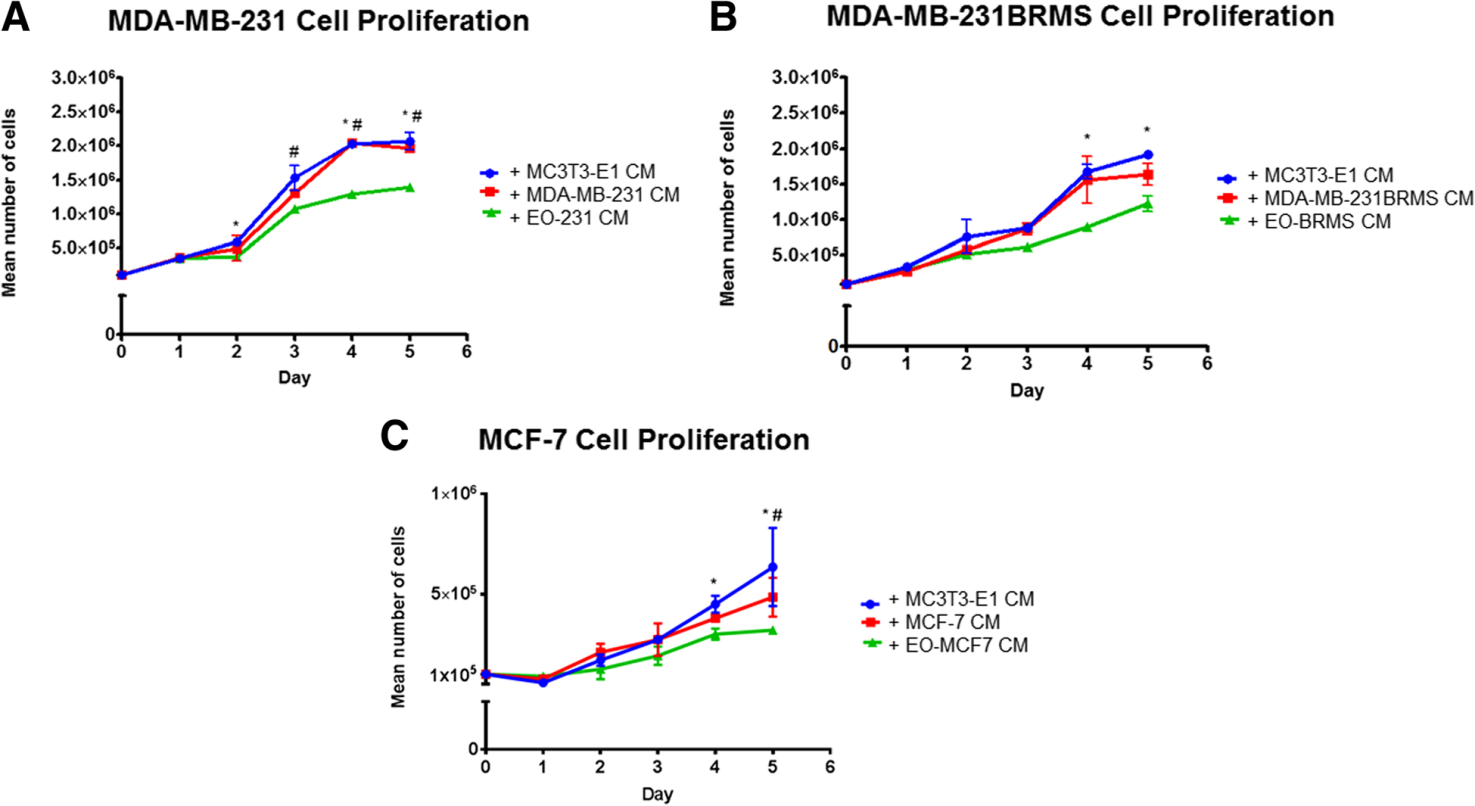
EO cells decrease breast cancer proliferation in vitro. MDA-MB-231, MDA-MB-231BRMS, or MCF-7 cells were plated at 1 × 10^5^ cells/cm^2^ in 35 × 10 mm dishes, then treated with either MC3T3-E1 CM, CM from cancer cells of the same type (e.g., MDA-MB-231 cells treated with MDA-MB-231 cell CM), or corresponding EO cell variant (e.g., MDA-MB-231 cells treated with EO-231 CM). Three individual replicates per time point per condition were plated. On days 1, 2, 3, 4, and 5, cancer cells were detached and counted using a hemocytometer. **a** MDA-MB-231 cells treated with either MC3T3-E1, MDA-MB-231, or EO-231 CM. **b** MDA-MB-231BRMS cells treated with either MC3T3-E1, MDA-MB-231BRMS, or EO-BRMS CM. **c** MCF-7 cells treated with either MC3T3-E1, MCF-7, or EO-MCF7 CM. *P < 0.05 MC3T3-E1 CM treatment vs. EO CM treatment; ^#^*P* < 0.05 cancer cell CM treatment vs. EO CM treatment. *N* = 3 per condition, per time point

Because EO cells exhibit characteristics different from “uneducated” osteoblasts, and since we found alterations in protein expression in the bone tumor microenvironment when EO cells were present, we next sought to determine the effect of EO cells on breast cancer cells. First, we exposed MDA-MB-231, MDA-MB-231BRMS, or MCF-7 human breast cancer cells to the respective EO cell conditioned media (i.e., EO-231, EO-BRMS, or EO-MCF7). As controls, cancer cells were exposed to OBCM, as well as the respective like-BCCM (i.e., MDA-MB-231 cells exposed to MDA-MB-231 cell conditioned media). Then, the breast cancer cells were counted on days 1, 2, 3, 4, and 5 to examine for alterations in proliferation. Interestingly, there was a statistically significant decrease in breast cancer proliferation upon exposure to EO-CM when compared to exposure to OBCM or like-BCCM in all cell lines examined (Fig. 6, green line compared to red and blue lines). The reduction in breast cancer cell proliferation was the most prominent at days 3–5 of cancer cell growth (Fig. 6a–c).

**Fig. 7:**
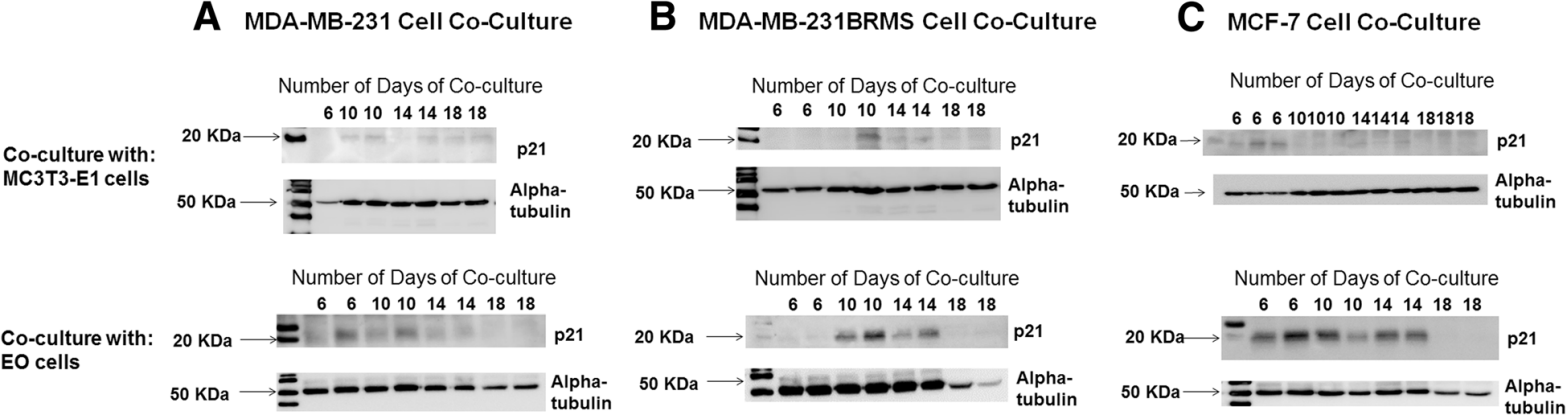
Human breast cancer cell p21 expression is increased over time in co-cultures with EO cells. MDA-MB-231, MDA-MB-231BRMS, or MCF-7 cells were co-cultured with either MC3T3-E1 osteoblasts or EO cells in 35 × 10 mm dishes. 6, 10, 14, or 18 days later, lysates were obtained and examined for human breast cancer cell expression of human-specific p21 by western blot. *N* = 3 dishes per condition, per time point. **a** MDA-MB-231 cells co-cultured with either MC3T3-E1 or EO-231 cells, **b** MDA-MB-231BRMS cells co-cultured with either MC3T3-E1 or EO-BRMS cells, **c** MCF-7 cells co-cultured with either MC3T3-E1 or EO-MCF7 cells

We next sought to determine if direct interaction with EO cells also affected breast cancer cell proliferation. We co-cultured human breast cancer cells with their respective EO cell (i.e., MDA-MB-231 cells plus EO-231 cells) for 6, 10, 14, or 18 days. Breast cancer cells co-cultured with vehicle-treated osteoblasts served as controls. Cell lysates were harvested and assayed for the presence of human-specific p21 (Additional file 14: Figure S14, species-specific, mouse osteoblast vs. human breast cancer cell), a cyclin-dependent kinase inhibitor that regulates cell cycle progression at the G1 checkpoint [86]. When compared to co-culture with vehicle-treated osteoblasts, co-culture with EO cells increased breast cancer cell-specific expression of p21 on days 10 and 14 (MDA-MB-231 and MDA-MB-231BRMS, as well as days 6, 10, and 14 (MCF-7 cells) (Fig. 7). No to negligible p21 expression was observed in all of the breast cancer cells by day 18 of co-culture (Fig. 7). These results suggest that EO cells regulate breast cancer proliferation in part via p21.

### EO cell cytokine expression

**Fig. 8:**
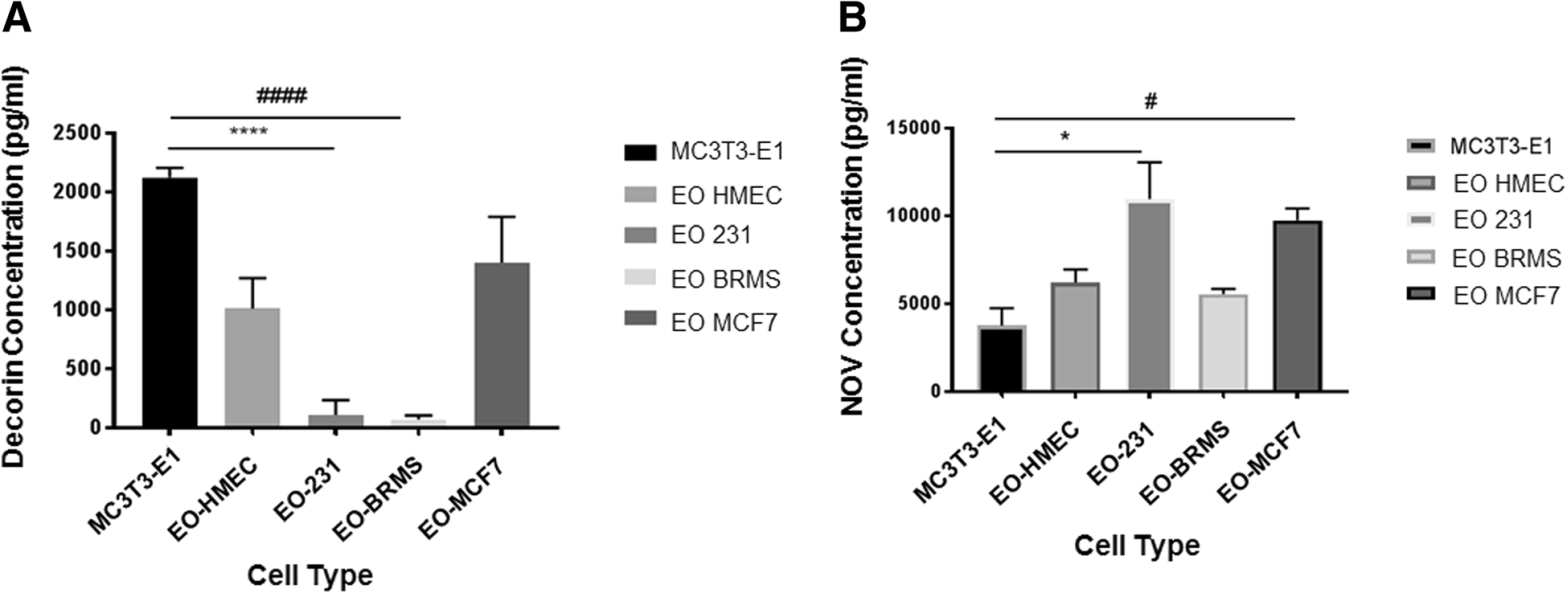
EO cells produce altered amounts of cytokines compared with normal osteoblasts. Differences in the levels of soluble factors between EOs and “uneducated” OBs were analyzed using a Raybiotech Quantibody Array Q4000, which surveys the protein expression of 400 cytokines. Conditioned medium was prepared from vehicle-treated MC3T3-E1, EO-HMEC, EO-231, EO-BRMS, or EO-MCF7 cells grown to confluence and analyzed for cytokine content via a RayBiotech Mouse Quantibody Array. Three individual conditioned media batches per analyte, per condition, were analyzed. A literature search of the biological properties of the top 10 proteins with altered levels in EOs compared to “uneducated” OBs was then carried out. Among the top 10 proteins with upregulated cytokine levels in EO cells, we focused on NOV. Among the top 10 proteins with downregulated levels in EO cells, we focused on decorin. **a** Decorin and **b** NOV cytokine expression. *P < 0.05; ^#^*P* < 0.05; ****P < 0.0001, ^####^*P* < 0.0001

Since we saw a decrease in breast cancer cell proliferation upon treatment with EO CM (Fig. 6) and direct co-culture with EO cells (Fig. 7), we next sought to determine the factors that may be responsible. To determine the differences in the levels of soluble factors between EOs and “uneducated” OBs, we analyzed the CM of EOs and naïve MC3T3-E1 osteoblasts (“uneducated” osteoblasts) using a Raybiotech Quantibody Array Q4000™, which surveys the protein expression of 400 cytokines. CM from EO-HMECs was used as an additional control. We carried out a literature search of the biological properties of the top 10 proteins with altered levels in EO cells compared to “uneducated” osteoblasts. Among the top 10 proteins with upregulated levels in EO cells, we decided to focus on NOV. Among the top 10 proteins with downregulated levels in EO cells, we focused on decorin. Decorin is a protein with anti-tumor properties and was explored as an anti-tumor agent in breast cancer [87–90]. Decorin is produced in high amounts by several key cells in the normal extracellular bone matrix, including osteoblasts, and is upregulated in normal stromal tissue [91]. NOV (CCN3) is a secreted, abundantly expressed extracellular matrix-associated signaling protein capable of regulating cellular activities including proliferation, migration, and cell adhesion and reduces the proliferation of glioblastoma cells and Ewing’s sarcoma [92–94]. NOV is an inhibitor of breast cancer invasion and is negatively correlated with late-stage bone metastatic breast cancer disease progression [94, 95]. Compared to vehicle-treated osteoblasts, we observed a statistically significant decrease in the expression of decorin in the CM of EO-231 and EO-BRMS cells (Fig. 8a). While the expression of decorin in EO-MCF7 cells was not statistically significant from vehicle-treated osteoblast CM, there was a trend toward a reduction in EO cell decorin expression (Fig. 8a). On the other hand, we observed a statistically significant increase in the amount of NOV expressed in the CM of EO-231 and EO-MCF7 cells when compared to vehicle-treated osteoblast CM (Fig. 8b). Interestingly, levels of NOV in the CM of EO-BRMS were comparable to that of vehicle-treated osteoblast CM (Fig. 8b).

**Fig. 9:**
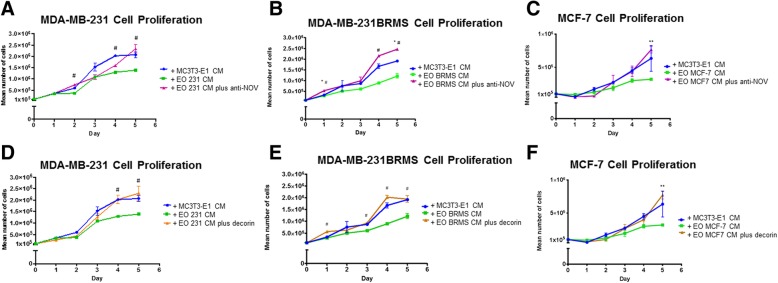
EO-altered breast cancer cell proliferation can be modulated by the cytokines NOV and decorin. MDA-MB-231, MDA-MB-231BRMS, or MCF-7 cells were plated at 1 × 10^5^ cells/cm^2^ in 35 × 10 mm dishes, then treated with either MC3T3-E1 CM, corresponding EO variant CM (e.g., MDA-MB-231 cells treated with EO-231 CM) or corresponding EO variant CM in the presence of either anti-NOV or decorin protein. Three individual replicates per time point per condition were plated. On days 1, 2, 3, 4, and 5, cancer cells were detached and counted using a hemocytometer. **a** MDA-MB-231 cells treated with either MC3T3-E1, EO-231 CM, or EO-231 CM plus anti-NOV. **b** MDA-MB-231BRMS cells treated with either MC3T3-E1, EO-BRMS CM, or EO-BRMS CM plus anti-NOV. **c** MCF-7 cells treated with either MC3T3-E1, EO-MCF7 CM, or EO-MCF7 CM plus anti-NOV. **d** MDA-MB-231 cells treated with either MC3T3-E1, EO-231 CM, or EO-231 CM plus decorin protein. **e** MDA-MB-231BRMS cells treated with either MC3T3-E1, EO-231 CM, or EO-231 CM plus decorin protein. **f** MCF-7 cells treated with either MC3T3-E1, EO-MCF7 CM, or EO-MCF7 CM plus decorin protein. ^#^*P* < 0.05, **P < 0.005 EO CM treatment vs. anti-NOV or decorin treatment. *N* = 3 per condition, per time point

We next wanted to determine if the rescue of decorin or NOV expression to that of expression levels seen in vehicle-treated osteoblasts would increase breast cancer cell proliferation (Fig. 9). Since NOV was increased in the CM of EO cells compared to the CM of vehicle-treated osteoblasts (Fig. 8b), we treated breast cancer cells with EO CM plus an antibody to NOV (Fig. 9a–c). Inhibition of NOV protein expression in the CM of EO cells caused an increase in breast cancer cell proliferation compared to breast cancer cell exposure to vehicle-treated osteoblast CM (purple line, Fig. 9a–c).

In a similar fashion, since the expression of decorin was reduced in the CM of EO cells compared to the CM of vehicle-treated osteoblasts (Fig. 8a), we treated breast cancer cells with EO CM plus recombinant decorin protein (Fig. 9d–f). Restoration of decorin protein in the CM of EO cells increased breast cancer cell proliferation to levels comparable of exposure to the vehicle-treated osteoblast CM (orange line, Fig. 9d–f).

**Fig. 10:**
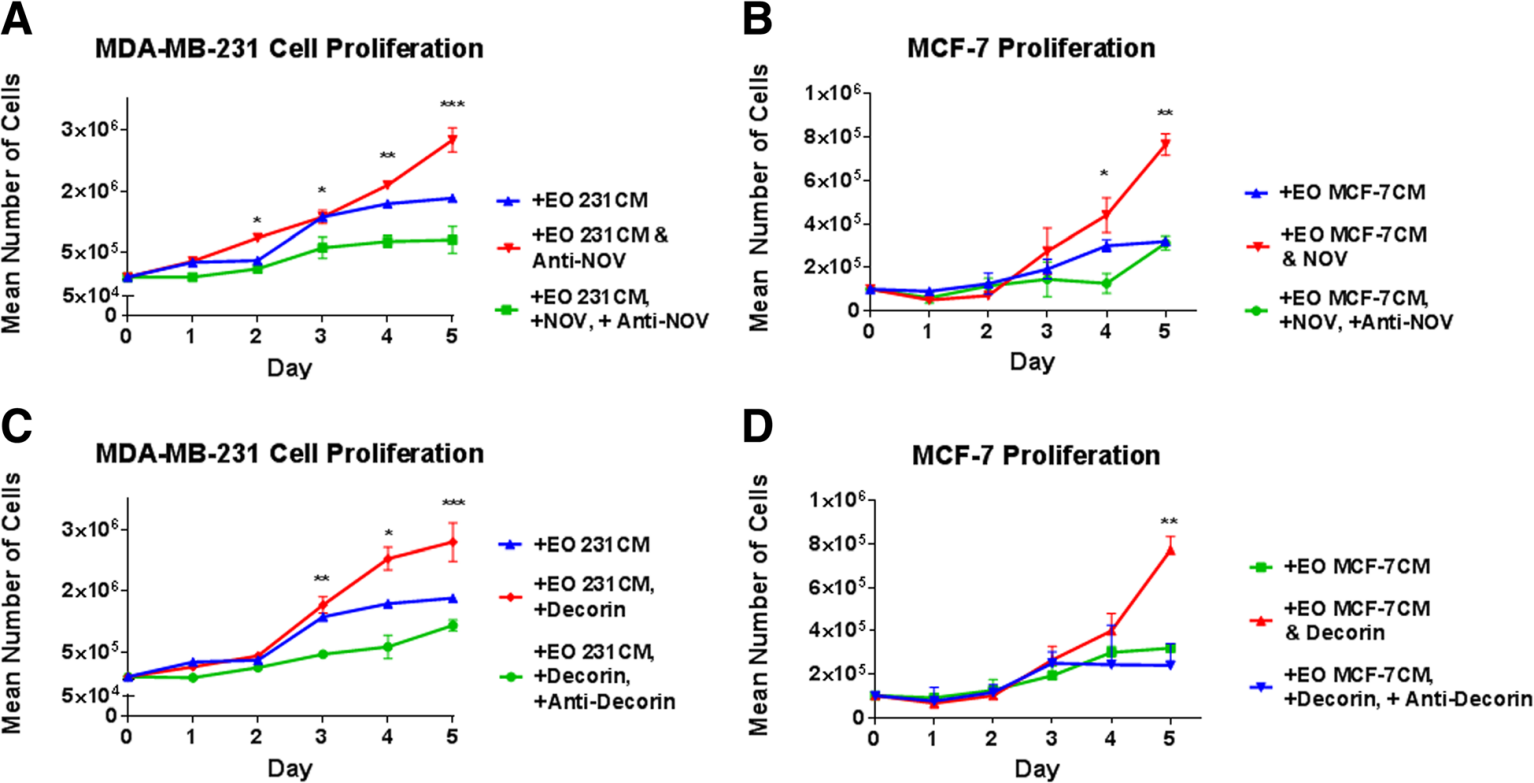
Rescue of decorin and NOV restores breast cancer cell proliferation to levels seen with the addition of EO-conditioned media. MDA-MB-231 or MCF-7 cells were plated at 1 × 10^5^ cells/cm^2^ in 35 × 10 mm dishes, then treated with either corresponding EO variant CM (e.g., MDA-MB-231 cells treated with EO-231 CM), corresponding EO variant CM in the presence of either anti-NOV or decorin protein or corresponding EO variant CM in the presence of either either anti-NOV PLUS recombinant NOV protein or decorin recombinant protein PLUS decorin antibody. Three individual replicates per time point per condition were plated. On days 1, 2, 3, 4, and 5, cancer cells were detached and counted using a hemocytometer. **a** MDA-MB-231 cells treated with either EO-231 CM, EO-231 CM plus anti-NOV, or EO-231 CM plus anti-NOV plus NOV protein. **b** MCF-7 cells treated with either EO-MCF7 CM, EO-MCF7 CM plus anti-NOV, or EO-MCF7 CM plus anti-NOV plus NOV protein. **c** MDA-MB-231 cells treated with either EO-231 CM, EO-231 CM plus decorin protein, or EO-231 CM plus decorin protein plus anti-decorin. **d** MCF-7 cells treated with either EO-MCF7 CM, EO-MCF7 CM plus decorin protein, or EO-MCF7 CM plus decorin protein plus anti-decorin. *P < 0.05, **P < 0.005, ***P < 0.001 EO CM plus anti-NOV or decorin vs. EO CM plus either anti-NOV plus NOV protein or decorin protein plus anti-decorin treatment. *N* = 3 biological replicates per condition, per time point

Finally, we assessed if the rescue of decorin or NOV expression in the presence of EO CM would return breast cancer proliferation to that seen upon exposure to EO CM alone (Fig. 10). We specifically focused these efforts on breast cancer cell subtypes that most directly reflect human disease, i.e., triple-negative (MDA-MB-231) and ER+ (MCF-7). The addition of recombinant NOV protein, antibody to NOV, plus EO CM (green line) restored both MDA-MB-231 (Fig. 10a) and MCF-7 (Fig. 10b) breast cancer cell proliferation to levels at or below that seen upon exposure to EO CM alone (blue line). Moreover, when an antibody to decorin was added to EO CM plus recombinant decorin protein, both MDA-MB-231 (Fig. 10c) and MCF-7 (Fig. 10d) (green line) breast cancer cell proliferation were restored to levels at or below that of the addition of EO CM alone (blue line). These results suggest that alterations in breast cancer cell proliferation by EO cells are mediated, in part, by NOV and/or decorin.

### NOV and decorin expression in the tumors of human patients with bone metastatic breast cancer

Since we identified EO cells in the tumor-bearing bones of human patients with metastatic breast cancer, and since EO cells express altered the amounts of NOV and decorin, which, in part, mediate breast cancer cell proliferation, we sought to assess the expression of NOV and decorin in human patient bone samples from individuals with bone metastatic breast cancer. Osteopontin was used as an indicator for osteoblasts (Figs. 2 and 5). Serial sections were utilized. In two of our patient samples, we observed the expression of decorin (Additional file 15: Figure S15A, B, arrows). When osteoblasts were located near the tumor tissue as in Additional file 15: Figure S15A, we observed decorin expression in 15% of the total cells. When osteoblasts were located away from the tumor cells (Additional file 15: Figure S15B, arrows), decorin was expressed in 43% of cells. Our findings correlate with previous reports in the literature suggesting that decorin expression is reduced in sites of bone metastases [89, 91]. When we examined for NOV, we observed NOV expression in 43% of cells (Additional file 15: Figure S15C, arrows). Since Additional file 15: Figure S15A and C are serial sections from the same patient sample, our results suggest that when osteoblasts are in the presence of tumors cells, decorin expression is low; however, NOV expression is moderate in comparison.

### Altered cell growth of breast cancer cells as induced by EO CM is due to altered breast cancer cell cycle

**Fig. 11:**
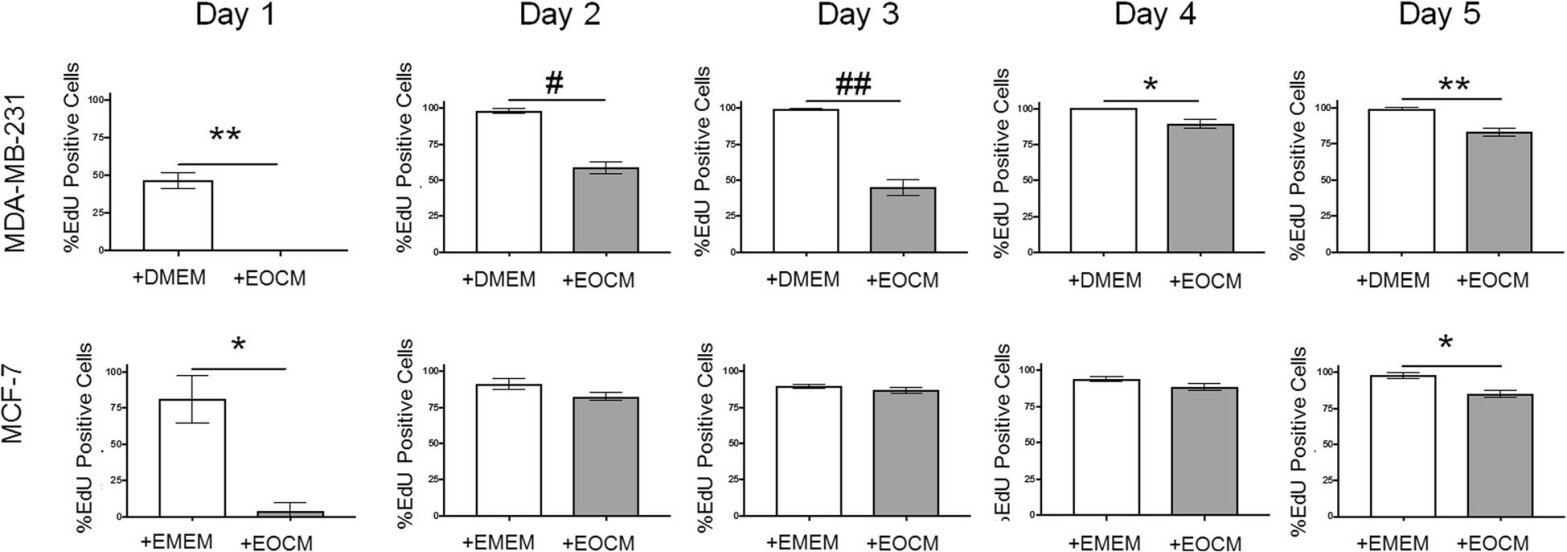
Breast cancer cells exposed to EO CM exhibit a reduced number of cells in S phase of cell cycle. MDA-MB-231 or MCF-7 cells were plated at 5 × 10^3^ cells in 4-well chamber slides in 50% growth media plus either 50% vehicle media (control) or 50% EO CM. Culture media was also supplemented with 0.5 μM 5-ethynyl-2-doxyuridine (EdU) for imaging using the Click-iT EdU Imaging Kit. Cells were maintained in media plus EdU for the entire length of the time course. On days 1, 2, 3, 4, and 5 post-treatment, cells were fixed with 4% paraformaldehyde and washed with PBS. For EdU imaging, cells were permeabilized, washed, then incubated with the Click-iT reaction cocktail. Cells were subsequently stained with DAPI, then visualized using fluorescence microscopy. *P < 0.01, **P < 0.005, ^#^*P* < 0.0005, ^##^*P* < 0.0001 +EO CM vs. +DMEM or +EMEM treatment. *N* = 3 biological replicates per condition, per time point

To determine if the alterations in breast cancer cell proliferation as induced by EO CM is due to the altered cell cycle, we carried out both propidium iodide staining (Additional file 16: Figure S16 and Additional file 17: Figure S17) to assess cell cycle state as well as incorporation of 5-ethynyl-2′-deoxyuridine (EdU) (Fig. 11) to detect cells undergoing S phase (DNA synthesis). First, we exposed MDA-MB-231 or MCF-7 human breast cancer cells to either vehicle media (+DMEM or +EMEM, control) or corresponding EO CM, then assessed alterations in the cell cycle at 1–5 days post-exposure. For MDA-MB-231 breast cancer cells, when normalized to a total number of cells analyzed per condition, on days 4 and 5 post-EO CM exposure, there were more cancer cells in G0/G1 populations compared to those cells exposed to vehicle control (Additional file 16: Figure S16). Furthermore, the number of cells found in the S phase of cell cycle was comparatively reduced in cancer cells exposed to EO CM vs. cancer cells exposed to vehicle medium. Moreover, the number of cells entering the G2/M phase was reduced in MDA-MB-231 exposed to EO CM on days 4 and 5 when compared to cells treated with vehicle media (Additional file 16: Figure S16).

We also assessed for EdU incorporation into MDA-MB-231 cells as measured by Click-IT technology. There were statistically significant decreases in the number of MDA-MB-231 breast cancer cells in the S phase of the cell cycle from days 1–5 upon treatment with EO CM when compared to treatment with vehicle media (Fig. 11).

We also assessed for alterations in the cell cycle of MCF-7 and ER+ cells upon exposure to EO CM. Distinct from MDA-MB-231 cells, the number of MCF-7 cells in G0/G1 was consistently higher upon exposure to EO CM, except for day 3 post-treatment (Additional file 17: Figure S17). When MCF-7 cells were examined for the S phase of cell cycle as measured by EdU incorporation, the number of cells in the S phase of the cell cycle was decreased upon treatment with EO CM on days 1–5 when compared to treatment with vehicle media, although statistical significance was only evident on days 1 and 5 (Fig. 11). Thus, alterations in breast cancer cell cycle, especially entrance into the S phase, are responsible, in part, for the decreases in breast cancer proliferation observed when triple-negative or ER+ breast cancer cells are exposed to EO CM.

## Discussion

The focus of this investigation was to understand the crosstalk that occurs in the bone microenvironment between osteoblasts and metastatic breast cancer cells during disease progression. We used an intratibial model of metastasis to the bone with admixes of osteoblasts and breast cancer cells to study the direct interactions between the two cell types (Figs. 1, 3, and 4). We identified two distinct populations of osteoblasts in vivo based on their marker expression of alpha-SMA and osteopontin (Fig. 1). We were able to replicate our in vivo results in vitro, whereby chronic osteoblast treatment with the conditioned media of triple-negative breast cancer cells resulted in osteoblasts that express osteopontin, but have reduced IL-6 and alpha-SMA protein expression (Fig. 2). We termed this population of osteoblasts “educated osteoblasts” due to their contact with bone metastatic breast cancer cells by direct or indirect means.

We also used de-identified human patient tissue with breast cancer metastases to the bone to identify osteoblasts in the bone tumor niche and describe the alterations to osteoblasts that occurred during interaction with metastatic breast cancer cells in the bone tumor niche in vivo. We interrogated the cancer-bearing bones of patients presenting with ER+ breast cancer. We specifically utilized bone tissue from patients with ER+ breast cancer because bone-disseminated breast cancer cells can remain in a growth suppressive-like state for up to decades in the bones of individuals with this disease subtype [96, 97]. Our data suggest that there are at least two distinct populations of osteoblasts in the tumor niche in vivo as defined by protein markers compared to non-cancer-bearing bone control (Figs. 1 and 5). In human tissue from patients with bone metastatic breast cancer, we found areas of RUNX2-positive, osteocalcin-positive expressing osteoblasts in the tumor microenvironment (Additional file 8: Figure S8). These same osteoblasts were assessed for their expression of IL-6, an inflammatory cytokine, and alpha-SMA, a marker of cells of the osteogenic lineage. Immunofluorescent staining of the RUNX2+/OCN+ cells revealed the presence of four different IL-6/alpha-SMA subpopulations: (1) IL-6+/alpha-SMA+, (2) IL-6+/alpha-SMA−, (3) IL-6−/alpha-SMA+, and (4) IL-6−/alpha-SMA−, the most abundant being IL-6+/alpha-SMA+ (~ 40%). Our in vitro data (Fig. 2) suggest that the IL-6+/alpha-SMA+ cells are “uneducated” osteoblasts. “Educated” osteoblasts (IL-6−/alpha-SMA−; Fig. 2), on the other hand, composed ~ 20% of the osteoblast population (Fig. 5). How is it possible that advanced bone lesions occurred in the presence of EO cells given that our data suggest EO cells to be cancer growth inhibitory? There are several possible answers to this question. First, it is possible that EO-derived mechanisms that suppress breast cancer proliferation in early disease are inhibited in late stage disease. Second, it is possible that the number of EO cells in the bone is greater in earlier stages of the disease, but become reduced in number during advanced disease, thus permitting the outgrowth of macrometastatic lesions. We have an ongoing work in our laboratory aimed at answering these questions. Of note, our prior data using advanced bone metastatic breast cancer mouse models suggest that increased osteoblast-derived expression of IL-6, among other proteins, drives metastatic progression in the bone [33]. EO cells described here express no to negligible amounts of IL-6 when compared to “uneducated osteoblasts” (Figs. 2 and 5). These results suggest that alterations in the expression of osteoblast-derived IL-6 may also influence the metastatic progression in the bone tumor microenvironment. Combined, these results suggest that osteoblasts may be altered and respond differently to breast cancer cells depending on the stage of the disease.

Based on our in vivo data, we hypothesized that EOs expressed a unique set of proteins as compared to “uneducated” osteoblasts. In fact, we determined that EOs express reduced amounts of alpha-SMA, inflammatory cytokines IL-6 and MCP-1, and decorin but express increased amounts of the extracellular matrix remodeling proteins collagen type I and MMP3, as well as NOV protein expression when compared to “uneducated” osteoblasts (Figs. 2 and 8). Both EOs and “uneducated” osteoblasts express alkaline phosphatase, osteopontin, and VEGF in similar amounts (Fig. 2). These results were unique to interaction with breast cancer cells or breast cancer cell CM; osteoblast expression of proteins upon treatment with the conditioned media from hTERT-HME1, a non-malignant mammary epithelial breast cancer line, was similar to osteoblast treatment with vehicle media (Fig. 2). These findings suggest that osteoblast expression of proteins associated with inflammation and matrix remodeling are altered when osteoblasts are in contact with bone metastatic breast cancer cells.

Bone-derived protein production in the presence of EOs was also examined ex vivo. Interestingly, the analysis of the endosteal niche of the tibia of mice inoculated via intratibial injection with either MC3T3-E1 “uneducated” osteoblasts plus MDA-MB-231GFP/Luc2 cells, EO-231 “educated” osteoblasts plus MDA-MB-231GFP/Luc2 cells, or MDA-MB-231GFP/Luc2 cells alone revealed that alkaline phosphatase, a marker of osteoblast differentiation, was only expressed in the tibia injected with an admix of EO-231 “educated” osteoblasts plus MDA-MB-231GFP/Luc2 cells, when compared to the tibia injected with either MC3T3-E1 “uneducated” osteoblasts plus MDA-MB-231GFP/Luc2 cells or MDA-MB-231GFP/Luc2 cells alone (Fig. 3, Table 1). In addition, minimal amounts of the inflammatory cytokine IL-6 was found in the trabecular bone of the tibia injected with EO-231 “educated” osteoblasts plus MDA-MB-231GFP/Luc2 cells, when compared to large amounts of IL-6 present in the trabecular bone of the tibia injected with MDA-MB-231GFP/Luc2 cells alone (Fig. 3, Table 1). These data suggest that both osteoblast differentiation was increased, and inflammatory cytokine expression was decreased in the tibia with EO cells present when compared to the tibia containing either “uneducated” osteoblasts plus breast cancer cells or breast cancer cells injected alone.

Of note, in the hematopoietic (bone marrow) niche, there were increased expression of the extracellular matrix remodeling proteins collagen type I and MMP3 in the tibia of mice injected with MDA-MB-231GFP/Luc2 cells alone, as compared to no to negligible expression of either protein in the bone marrow of the tibia of mice injected with either MC3T3-E1 “uneducated” osteoblasts or EO-231 “educated” osteoblasts (Fig. 4, Table 1). Importantly, tumors that formed in the tibia of mice injected with EO-231 cells plus MDA-MB-231GFP/Luc2 cells had low to no expression of any of the proteins examined (Additional file 6: Figure S6, Table 1), whereas tumors that formed in the tibia of mice injected with either MDA-MB-231GFP/Luc2 cells alone or MC3T3-E1 osteoblasts plus MDA-MB-231GFP/Luc2 cells contained moderate to high levels of vascularization and extracellular matrix remodeling markers (Additional file 6: Figure S6, Table 1). These results suggest that the presence of EO cells in tumors suppresses the expression of extracellular matrix markers, inflammatory cytokines, and neovascularization markers.

We also found that the CM from EOs reduced the proliferation of both triple-negative and ER+ breast cancer cells (Fig. 6) and that this effect could be mediated by either NOV or decorin (Figs. 9 and 10). These data suggest that factors involved in NOV or decorin signaling may, in part, help to facilitate suppressed breast cancer growth by EOs. NOV (neuroblastoma overexpressed; CCN3) is a matricular protein widely expressed in high levels by germ cells [98]. NOV supports cell adhesion and survival, as well as induces directed cell chemotaxis [98]. Interestingly, NOV is a negative regulator of bone deposition via inhibition of BMP2 signaling pathways [99]. Decorin, on the other hand, is a small leucine-rich proteoglycan produced by osteogenic cells. Importantly, decorin has emerged as a promising anti-cancer agent produced by normal cells [89]. Decorin suppresses cancer cell growth by blockade of cancer cell TGF-beta signaling [89]. In addition, data have shown that osteoblasts naturally express decorin [100], implying that osteoblasts may naturally possess inhibitory effects toward metastatic cancer cells.

Interestingly, it is reported that decorin inhibits the growth of colon carcinoma cells by the upregulation of p21 [101]. Similarly, we observed an increase in the expression of human-specific p21 expression in breast cancer cells when directly co-cultured with EOs (Fig. 7). These effects were evident with both triple-negative and ER+ human breast cancer cells and were most apparent upon 6–14 days of co-culture with EOs (Fig. 7). Breast cancer cell co-culture with “uneducated” osteoblasts did not elicit increased human-specific breast cancer cell p21 expression suggesting that these effects occur as a result of a unique osteoblast subpopulation with inhibitory or anti-tumor effects toward breast cancer cells.

To determine if alterations in breast cancer cell proliferation as generated by EO CM were a result of altered breast cancer cell cycle, we carried out both propidium iodide staining and EdU staining of breast cancer cells (Fig. 11, Additional file 16: Figure S16, and Additional file 17: Figure S17). We observed a statistically significant reduction in the number of breast cancer cells entering the S phase of the cell cycle as determined by EdU incorporation (Fig. 11). This finding was recapitulated by propidium iodide staining in both triple-negative and ER+ breast cancer cells, especially on days 4 and 5 post-exposure to EO CM (Additional file 16: Figure S16 and Additional file 17: Figure S17). It should be noted that propidium iodide staining cannot distinguish resting/quiescent cells (G0) from cells in the G1 phase [102]. Thus, the determination of cells entering quiescence, that is dormancy, is unable to be detected by this method [102]. Regardless, our in vitro data suggest that breast cancer cell cycle is affected by EO cells at the S phase, as evidenced by both a reduction in the number of breast cancer cells incorporating EdU (Fig. 11), and by an increase in breast cancer cell expression of p21 upon direct co-culture with EO cells (Fig. 7). p21 is a cell cycle inhibitor that acts during entry into the S phase, among other functions [103].

**Fig. 12:**
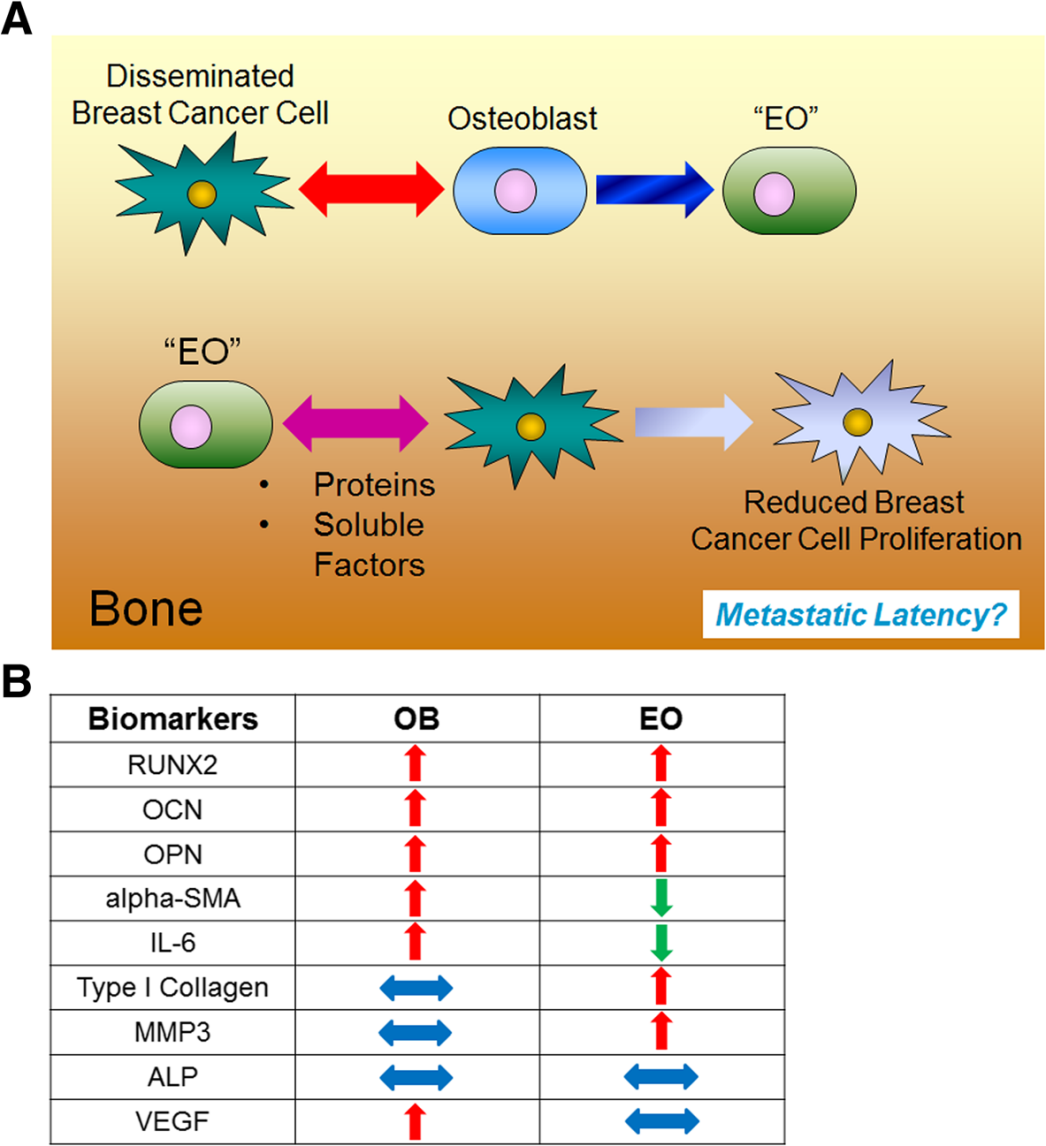
EO cells are distinct from “uneducated osteoblasts” and suppress breast cancer cell proliferation in the bone. **a** When disseminated breast cancer cells first enter the bone microenvironment, they engage in crosstalk with osteoblasts, leading to the generation of a subpopulation of osteoblasts defined as “educated osteoblasts.” “Educated osteoblasts,” in turn, engage in crosstalk with bone-disseminated breast cancers via proteins and soluble factors, among others, leading to a reduction in breast cancer cell proliferation in the bone microenvironment that may play a role in metastatic latency. **b** EO marker panel key: protein alterations in RUNX2, OCN, OPN, aSMA, IL-6, type I collagen, MMP3, ALP, and VEGF distinguish EOs from OBs. Red arrow indicates high protein expression, green arrow indicates low protein expression, and blue sideways arrow indicates average protein expression as determined by western blot and/or immunofluorescence

It is becoming increasingly evident that osteoblasts in the bone microenvironment play vital roles in cancer cell progression in bone [19, 32–36]. According to one study that used dynamic longitudinal imaging and intravital microscopy, osteoblast cells in the endosteal niche suppressed multiple myeloma cancer cell proliferation and maintained bone-disseminated cancer cells in a dormant state during early disease [104]. This was in contrast to the effects seen when multiple myeloma cells were treated with the CM of osteoclasts, which promoted myeloma cell proliferation [104]. Another study demonstrated that co-culture of osteoblasts plus prostate cancer cells resulted in a reduction of prostate cancer cell proliferation [105]. And another group demonstrated that proteins produced by osteoblasts induced prostate cancer cell dormancy both in vitro and in vivo [106]. Our data provided in this publication provide further support that crosstalk with osteoblasts reduces cancer cell proliferation. To our knowledge, we are the first laboratory to extend these findings to bone metastatic breast cancer, whereby we discovered a subpopulation of osteoblasts called “educated osteoblasts” (Figs. 1, 2, and 5) that reduce breast cancer proliferation (Fig. 6) and entry into the S phase of cell cycle (Fig. 7), in part via the proteins NOV and decorin (Figs. 9 and 10). These data suggest that this mechanism may be one way in which bone-disseminated breast cancer cells enter metastatic latency (dormancy) in the bone microenvironment (Fig. 12a). These data also suggest that osteoblasts possess growth inhibitory properties, a trait capable of exploitation, that may be used to both promote metastatic latency and prevent metastatic progression in the bone. Metastatic latency (dormancy) is a period of cancer progression whereby tumor cells that have disseminated from a primary tumor to secondary sites are able to persist in a growth suppressive-like state for up to decades [96]. Dormant cancer cells are often characterized by their reduced cell growth and G_0_-G_1_ arrest [96].

Mounting evidence suggests bone metastatic breast cancer cells act on osteoblasts in the tumor niche to alter osteoblast production of proteins [32, 33, 57, 58]. Our new in vivo evidence suggests bone metastatic breast cancer cells induce a non-normal osteoblast state of osteopontin (OPN) high, alpha-smooth muscle actin (aSMA) low, and IL-6 low (Figs. 1, 2, 5, and 12b). We define this subpopulation of osteoblasts as “educated osteoblasts.” Educated osteoblasts are distinct in their expression of biomarkers compared to naïve, “uneducated osteoblasts” (Figs. 1, 2, 5, and 12b). Our evidence suggests that educated osteoblasts, in turn, engage in crosstalk with bone-disseminated breast cancer cells via proteins (i.e., decorin and NOV) and soluble factors, among others, which lead to a reduction in breast cancer cell proliferation via an increase in the cell cycle inhibitor p21 and reduction in the number of breast cancer cells entering the S phase of cell cycle. Our data suggest that this mechanism may be one way in which the bone microenvironment both suppresses breast cancer cell proliferation and engages disseminated breast cancer cells into metastatic latency (dormancy) in early disease (Fig. 12a).

## Conclusions

Much less attention is given to osteoblast interactions with tumor cells at sites of bone metastases. This is partly due to the observations that osteoblast populations are reduced at sites of advanced osteolysis. However, we propose that osteoblasts may indeed be valuable endogenous targets to aid in the restriction of cancer cell growth in the niche in concert with therapeutic drugs to kill the cancer cells. Our data here suggest there is a subpopulation of osteoblasts that demonstrate a functional role in suppressing breast cancer cell growth; a property capable of exploitation (Fig. 12a). For these reasons, osteoblasts and EOs are intuitively suitable candidates for therapeutic targeting.

## Additional files

Additional file 1: **Figure S1.** Murine MC3T3-E1 cells express FOXN1 and are distinguishable from native endogenous osteoblasts in vivo. MC3T3-E1 cells and NIH-3T3 fibroblasts (control) were plated then maintained in growth media in 35 × 10 mm dishes. A) For western blot, cells were grown to ~ 80% confluence, then growth media were removed, cells washed, and lysates prepared. Lysates were examined for the expression of murine FOXN1 protein by western blot. *N* = 3 dishes per condition. Shown is a representative example. B-C) For immunochemistry, cells were grown to ~ 80% confluence and stained for the expression of B) FOXN1 (green, 488), or C) FOXN1 (yellow, arrows) and RUNX2 (red, 594) by immunofluorescence. TB = trabecular bone. *N* = 3 dishes per condition. Shown are representative examples. Scale bar = 50 μm. (TIF 5602 kb)

Additional file 2: **Figure S2.** Antibody validation using MC3T3-E1 mouse osteoblasts. MC3T3-E1 osteoblast cells were grown to ~ 90% confluence then fixed and stained for alpha-SMA, MCP-1, MMP-3, collagen type I, IL-6, and VEGF. *10 day-differentiated MC3T3-E1 cells were used to stain for ALP and OPN. Scale bar = 50 μm. (TIF 5116 kb)

Additional file 3: **Figure S3.** Antibody validation using MDA-MB-231 human breast cancer cells. MDA-MB-231 cells were grown to ~ 90% confluence then fixed and stained for VEGF, MCP-1, MMP-3, collagen type I, IL-6, alpha-SMA, ALP, OPN, and Ki67. Scale bar = 50 μm. (TIF 5674 kb)

Additional file 4: **Figure S4.** Secondary antibodies were not reactive against mouse or human cells. A) MC3T3-E1 murine osteoblasts and B) MDA-MB-231 human breast cancer cells were grown to 75–80% confluence then fixed and stained. Primary antibody was replaced with antibody diluent to generate delete. Secondary antibodies were donkey anti-goat 488, goat anti-rabbit 488, and donkey anti-mouse 594. Scale bar = 50 μm. (TIF 4438 kb)

Additional file 5: **Figure S5.** Intratibial injection tumor formation with MDA-MB-231, MC3T3-E1, and EO Cells. Athymic nude mice were injected via intratibial injection with an admix of MDA-MB-231GFP/Luc2 human breast cancer cells plus either EO-231 cells or MC3T3-E1 osteoblasts, or MDA-MB-231GFP/Luc2 cells alone. Eight weeks later, mice were euthanized and their tibia harvested. Sections were stained for green fluorescent protein via immunofluorescence. Scale bar = 50 μm. (TIF 1109 kb)

Additional file 6: **Figure S6.** Unique protein expression occurs with EO cell presence in tumor-bearing bones. Athymic nude mice were injected via intratibial injection with an admix of MDA-MB-231GFP/Luc2 human breast cancer cells plus either EO-231 cells or MC3T3-E1 osteoblasts, or MDA-MB-231GFP/Luc2 cells alone. Eight weeks later, mice were euthanized and their tibia harvested. Tibia sections from athymic mice were prepared as described in the “Materials and methods” section. Sections were stained for osteopontin, alkaline phosphatase, VEGF, alpha-smooth muscle actin, MMP3, collagen type I, MCP-1, IL-6, and green fluorescent protein via immunofluorescence. As shown on the tibia at left, the black box represents the positioning of the tumor in the examples shown and illustrates locations in the bone where the images were taken. At least three independent, serial sections were stained per bone, and three bones examined per condition. Shown are representative images. The tumor microenvironment was examined via fluorescent microscopy. Scale bar = 50 μm. (TIF 15079 kb)

Additional file 7: **Figure S7.** OCN and RUNX2 antibody optimization using NHOst human osteoblasts. NHOst cells were grown to confluence then fixed and stained for expression of osteocalcin (red, 594) and RUNX2 (green, 488) by immunofluorescence. Scale bar = 50 μm. (TIF 1206 kb)

Additional file 8: **Figure S8.** Osteoblasts are present in patient samples of bone metastatic breast cancer. Human patient samples of bone metastatic breast cancer were stained using immunofluorescence for RUNX2 (green, 488) and osteocalcin (OCN, red, 594). Osteoblasts were identified A) adjacent to tumor cells and B) away from tumor cells. T = tumor; O, arrows =osteoblast. DAPI, nuclear stain. Scale bar = 50 μm. (TIF 1441 kb)

Additional file 9: **Figure S9.** Antibody optimization using NHOst human osteoblasts. NHOst cells were grown to confluence then fixed and stained for expression of osteopontin (green, 488), IL-6 (green, 488), alpha-SMA (red, 594), and alkaline phosphatase (green, 488) by immunofluorescence. Scale bar = 50 μm. (TIF 2887 kb)

Additional file 10: **Figure S10.** EO cells differentiate and mineralize. EO cells were plated at 1 × 10^5^ cells/cm^2^ in 35 × 10 mm dishes, then grown to confluence. MC3T3-E1 cells were plated in growth media at 1 × 10^5^ cells/cm^2^ in 35 × 10 mm dishes. Twenty-four hours later, growth media were removed from MC3T3-E1 cells, and replaced with differentiation media. MC3T3-E1 cells were differentiated for 20 days. For both cell groups, media were exchanged every third day. Once cells reached confluence (EO cells) or were differentiated to 20 days (MC3T3-E1 cells), media were removed, cells fixed, then stained for either alkaline phosphatase expression using Napthol AS-BI phosphate, Tris, and Fast Blue RR salt; or Von Kossa using silver nitrate. Cells were photographed using a light microscope. Three biological replicates were completed per condition. Shown are representative images. (TIF 3499 kb)

Additional file 11: **Figure S11.** EO cells have altered rates of proliferation compared to normal osteoblasts. Vehicle-treated MC3T3-E1, EO-HMEC, EO-231, EO-BRMS, or EO-MCF7 cells were plated at 1 × 10^5^ cells/cm^2^ in 35 × 10 mm dishes. Three individual replicates per condition were plated. On days 2, 4, 6, 8, and 10, cells were detached and counted using a hemocytometer. A) Statistical significance was calculated at each time point (i.e., days 2, 4, 6, 8, and 10) and represented by *P < 0.05 EO-HMEC vs. vehicle-treated MC3T3-E1, **P < 0.05 EO-231 vs. vehicle-treated MC3T3-E1, ^#^*P* < 0.05 EO-BRMS vs. vehicle-treated MC3T3-E1, ^*P* < 0.05 EO-MCF7 vs. vehicle-treated MC3T3-E1. B) MC3T3-E1 vs. EO-HMEC, *P < 0.05, ^^*P* < 0.01, **P < 0.005; C) MC3T3-E1 vs. EO-231, *P < 0.05, ^*P* < 0.01, **P < 0.005; D) MC3T3-E1 vs. EO-BRMS, *P < 0.05, ^#^*P* < 0.0005; E) MC3T3-E1 vs. EO-MCF7, *P < 0.05, **P < 0.005, ***P < 0.0001. (TIF 3171 kb)

Additional file 12: **Figure S12.** EO cells have altered F-actin organization compared to normal osteoblasts. A) MC3T3-E1 cells were plated in 35 × 10 mm^2^ dishes at 1 × 10^5^ cells/cm^2^ in growth media. Cells were grown to ~ 70% confluence. Growth medium was exchanged every third day. EO cells were plated in 35 × 10 mm dishes at 1 × 10^5^ cells/cm^2^ and grown in three parts 1.5× differentiation medium plus 1 part either MDA-MB-231, MDA-MB-231BRMS, or MCF-7 breast cancer-conditioned medium or hTERT-HME1 mammary epithelial cell-conditioned medium. Cells were grown to ~ 70% confluence. Media were changed every second day. For F-actin staining, media were removed, cells washed with PBS, then cells stained for F-actin expression using a phalloidin stain. Three biological replicates were carried out per condition. Shown are representative images. Arrows point to F-actin deposits. B) Quantification of F-actin deposits. ***P = 0.0004, ****P < 0.0001. (TIF 3065 kb)

Additional file 13: **Figure S13.** EO cells do not exhibit autophagic lysosomes. MC3T3-E1, EO-231, and EO-BRMS cells were plated in 35 × 10 mm dishes, then stained for LC3 expression via immunofluorescence. MC3T3-E1 cells treated with serum-free media for 48 h served as a positive control. *N* = 3 dishes per condition. Shown are representative images. (TIF 1721 kb)

Additional file 14: **Figure S14.** Human-specific p21 antibody does not cross-react with murine cells. Murine MC3T3-E1 osteoblasts differentiated to 20 days and human MCF-7 lysates were collected and analyzed for human-specific p21 by western blot. *N* = 2 biological replicates per condition. (TIF 268 kb)

Additional file 15: **Figure S15.** NOV and decorin are expressed in the tumor-bearing bones of human patients with bone metastatic breast cancer. Serial sections of human patient samples of bone metastatic breast cancer were stained using immunofluorescence for decorin (yellow), NOV (yellow), and osteopontin (OPN, red). A) Patient 1 staining for decorin; B) patient 2 staining for decorin; C) patient 1 staining for NOV. *N* = 2 slides stained per protein using serial sections. At least three patient samples were stained per protein. Shown are representative examples. OB = osteoblast; T = tumor; DAPI, nuclear stain. Scale bar = 50 μm. (TIF 2012 kb)

Additional file 16: **Figure S16.** Altered cell growth of MDA-MB-231 cells treated with EO CM is due to altered cell cycle. MDA-MB-231 cells were plated at 1 × 10^5^ cells/cm^2^ in 35 × 10 mm dishes. Twenty-four hours later, growth media were removed and cancer cells treated with 1 ml breast cancer growth media plus either a) vehicle media (DMEM, control) or b) EO-conditioned media. Breast cancer cells were fixed for at least 2 h with 95% cold ethanol beginning on day 1 after plating, and continuing every day for 5 days. For propidium iodide staining, ethanol was decanted and fixed cells washed once with PBS. Cells were resuspended in a solution of 50 ng/ml propidium iodide, 100 ng/ml RNAse A, and PBS per 1 × 10^6^ cells and incubated for 30 min in the dark at room temperature. Stained cells were analyzed for propidium iodide staining using a BD LSRII flow cytometer at excitation 535 nm and emission at 617 nm. A minimum of 10,000 events were counted per sample. Cell cycle phase was analyzed using BDFACS Diva software and FlowJo software. Three biological replicates per time point were counted per condition. Shown are representative images. (TIF 2281 kb)

Additional file 17: **Figure S17.** Altered cell growth of MCF-7 cells treated with EO CM is due to altered cell cycle. MCF-7 cells were plated at 1 × 10^5^ cells/cm^2^ in 35 × 10 mm dishes. Twenty-four hours later, growth media were removed and cancer cells treated with 1 ml breast cancer growth media plus either a) vehicle media (EMEM, control) or b) EO-conditioned media. Breast cancer cells were fixed for at least 2 h with 95% cold ethanol beginning on day 1 after plating, and continuing every day for 5 days. For propidium iodide staining, ethanol was decanted and fixed cells washed once with PBS. Cells were resuspended in a solution of 50 ng/ml propidium iodide, 100 ng/ml RNAse A, and PBS per 1 × 10^6^ cells and incubated for 30 min in the dark at room temperature. Stained cells were analyzed for propidium iodide staining using a BD LSRII flow cytometer at excitation 535 nm and emission at 617 nm. A minimum of 10,000 events were counted per sample. Cell cycle phase was analyzed using BDFACS Diva software and FlowJo software. Three biological replicates per time point were counted per condition. Shown are representative images. (TIF 2263 kb)

## Abbreviations

231: MDA-MB-231 cells
ALP: Alkaline phosphatase
alpha-MEM: Alpha Minimal Essential Media
alpha-SMA: Alpha-smooth muscle actin
ANOVA: Analysis of variance
BCCM: Breast cancer-conditioned medium
BRMS: Breast cancer metastasis suppressor
CM: Conditioned medium
CXCL12: C-X-C motif chemokine 12
CXCR4: C-X-C chemokine receptor type 4
DAPI: 4′,6-Diamidino-2-phenylindole
DMEM: Dulbecco’s minimal essential media
EdU: 5-Ethynyl-2-doxyuridine
EO: Educated osteoblast
ER+: Estrogen receptor-positive
FBS: Fetal bovine serum
FSP: Fibroblast-specific protein
GFP: Green fluorescent protein
GRO-alpha: Growth-related oncogene-alpha
HRP: Horseradish peroxidase
IL-6: Interleukin-6
IL-8: Interleukin-8
IVIS: Intravital imaging system
LC3: Light chain 3
MCP-1: Monocyte chemoattractant protein-1
MEBM: Mammary epithelial basal medium
MMP3: Matrix metalloproteinase 3
NOV/CCN3: Neuroblastoma overexpressed
OB: Osteoblast
OBCM: Osteoblast-conditioned medium
OCN: Osteocalcin
OPN: Osteopontin
PBS: Phosphate-buffered saline
PFA: Paraformaldehyde
RIPA: Radioimmunoprecipitation assay buffer
S: Synthesis phase of cell cycle
TBS: Tris-buffered saline
TGF-beta: Tumor growth factor-beta
VEGF: Vascular endothelial growth factor
VM: Vehicle media
