# Supplementary figures and images for: Osteoblasts are “educated” by crosstalk with metastatic breast cancer cells in the bone tumor microenvironment
Source: Breast Cancer Res. 2019 Feb 27;21:31. doi: 10.1186/s13058-019-1117-0 (PMC6391840; doi:10.1186/s13058-019-1117-0)

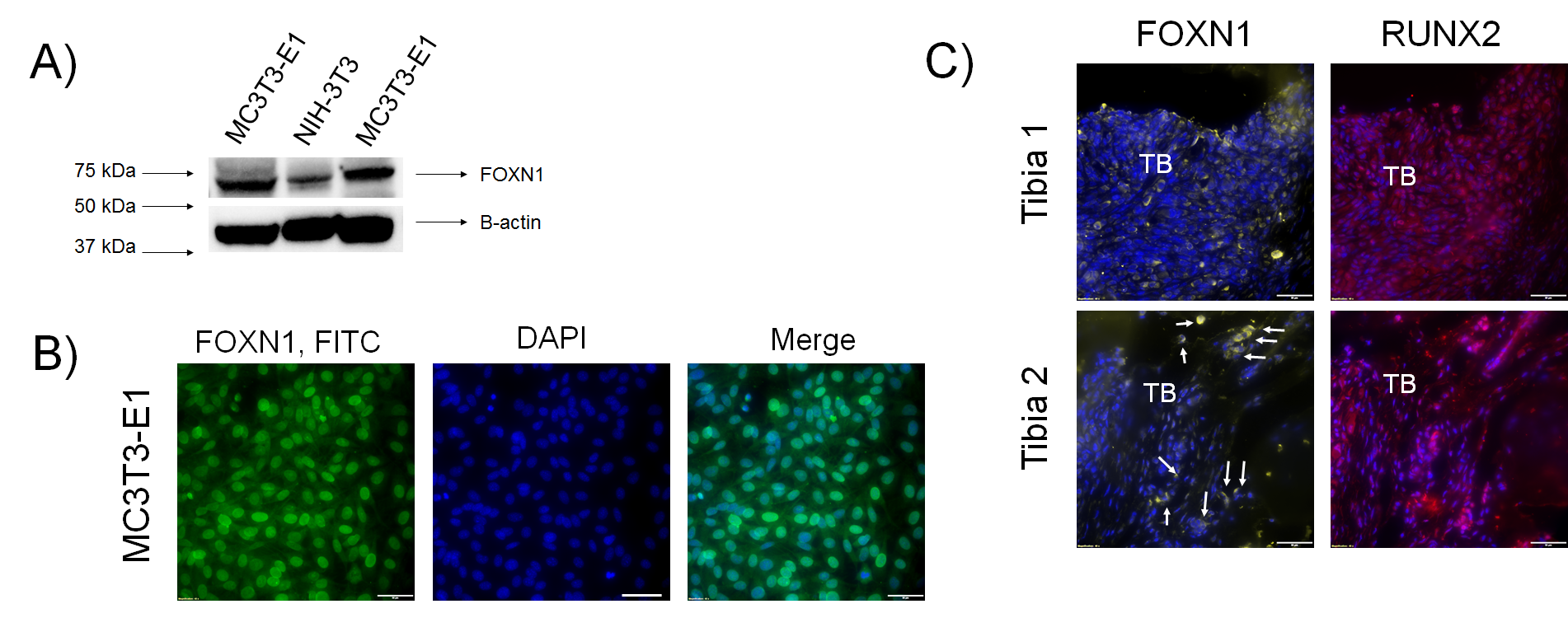

Supplement: Supplementary file 1 — Figure S1. Murine MC3T3-E1 cells express FOXN1 and are distinguishable from native endogenous osteoblasts in vivo. MC3T3-E1 cells and NIH-3T3 fibroblasts (control) were plated then maintained in growth media in 35 × 10 mm dishes. A) For western blot, cells were grown to ~ 80% confluence, then growth media were removed, cells washed, and lysates prepared. Lysates were examined for the expression of murine FOXN1 protein by western blot. N = 3 dishes per condition. Shown is a representative example. B-C) For immunochemistry, cells were grown to ~ 80% confluence and stained for the expression of B) FOXN1 (green, 488), or C) FOXN1 (yellow, arrows) and RUNX2 (red, 594) by immunofluorescence. TB = trabecular bone. N = 3 dishes per condition. Shown are representative examples. Scale bar = 50 μm. (TIF 5602 kb) [file 13058_2019_1117_MOESM1_ESM.tif]

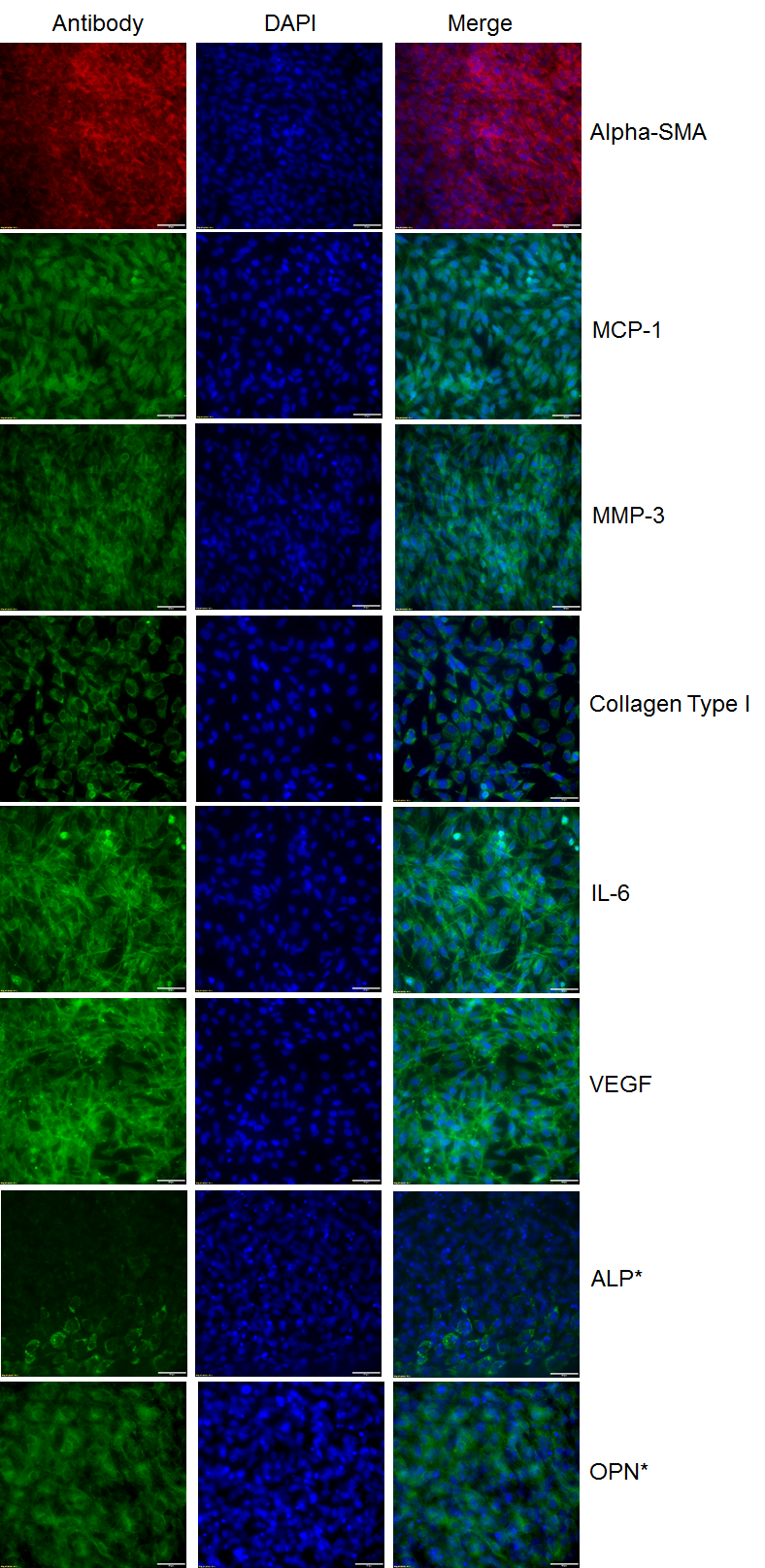

Supplement: Supplementary file 2 — Figure S2. Antibody validation using MC3T3-E1 mouse osteoblasts. MC3T3-E1 osteoblast cells were grown to ~ 90% confluence then fixed and stained for alpha-SMA, MCP-1, MMP-3, collagen type I, IL-6, and VEGF. *10 day-differentiated MC3T3-E1 cells were used to stain for ALP and OPN. Scale bar = 50 μm. (TIF 5116 kb) [file 13058_2019_1117_MOESM2_ESM.tif]

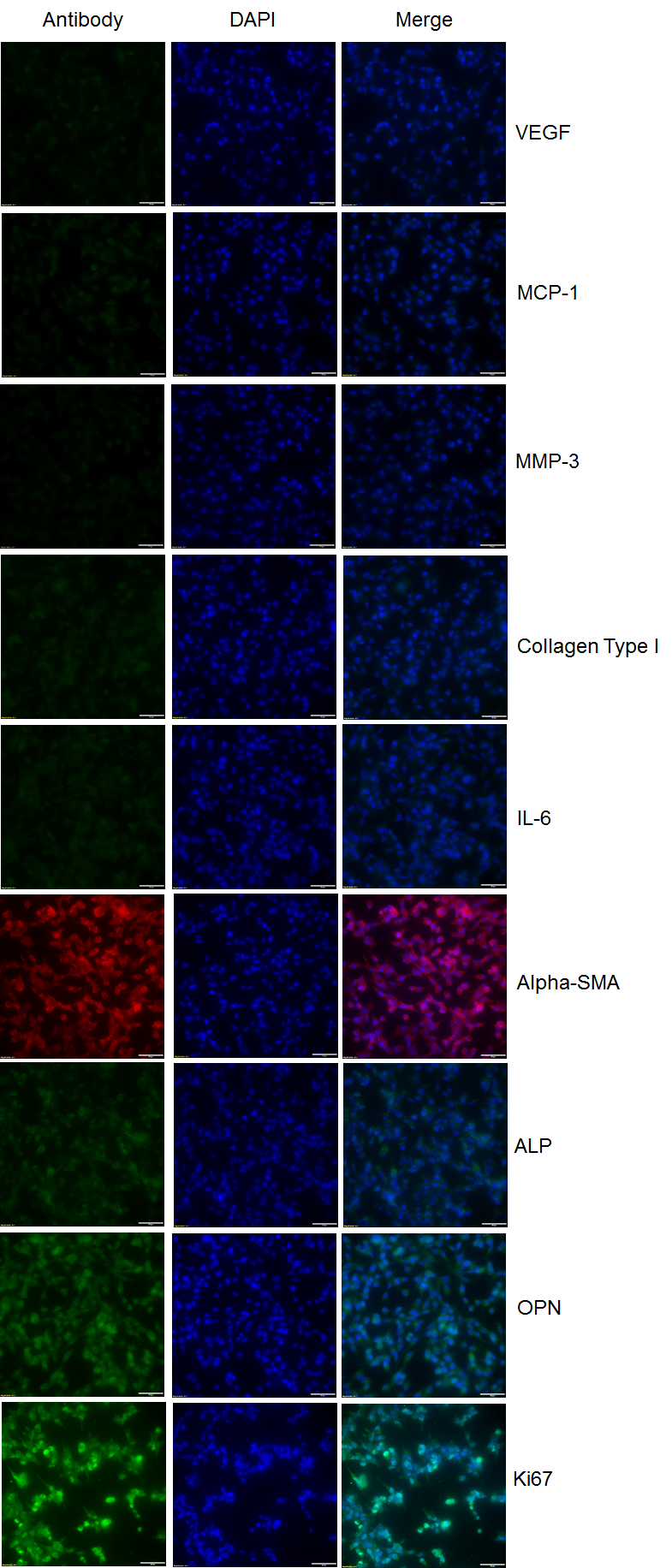

Supplement: Supplementary file 3 — Figure S3. Antibody validation using MDA-MB-231 human breast cancer cells. MDA-MB-231 cells were grown to ~ 90% confluence then fixed and stained for VEGF, MCP-1, MMP-3, collagen type I, IL-6, alpha-SMA, ALP, OPN, and Ki67. Scale bar = 50 μm. (TIF 5674 kb) [file 13058_2019_1117_MOESM3_ESM.tif]

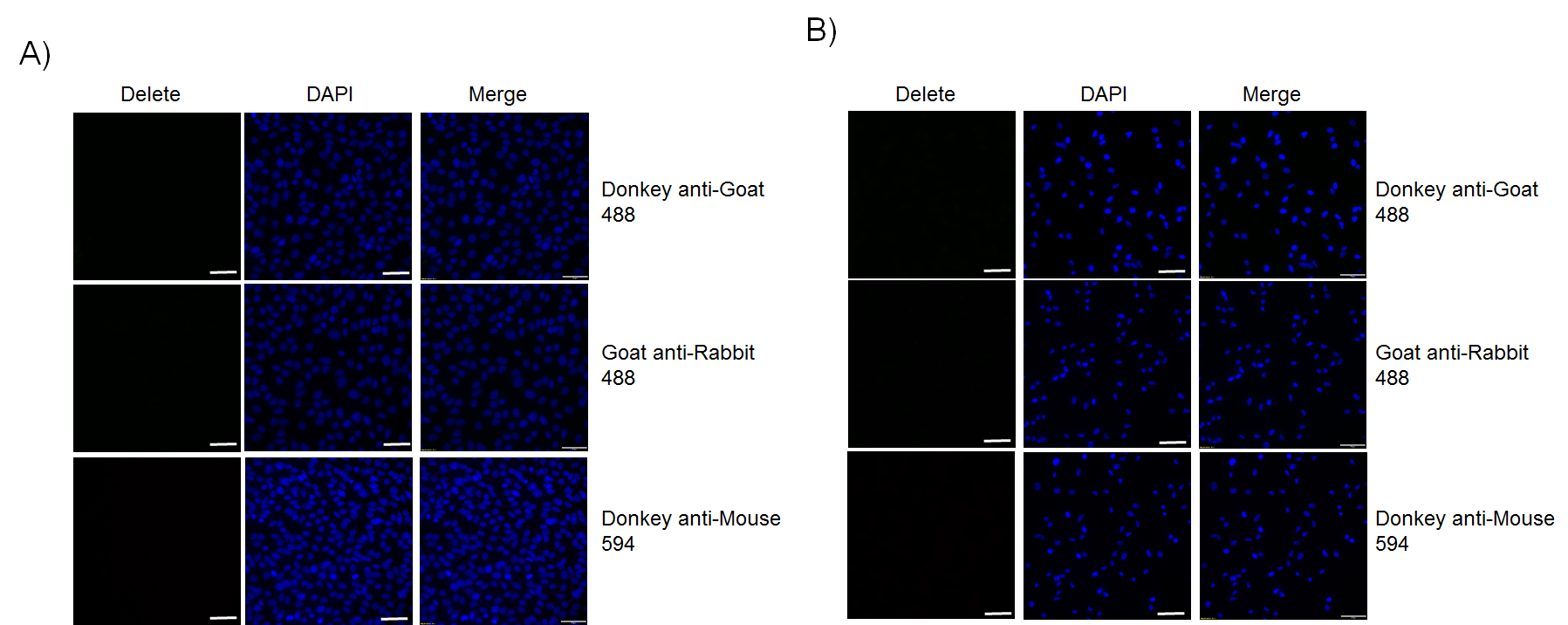

Supplement: Supplementary file 4 — Figure S4. Secondary antibodies were not reactive against mouse or human cells. A) MC3T3-E1 murine osteoblasts and B) MDA-MB-231 human breast cancer cells were grown to 75–80% confluence then fixed and stained. Primary antibody was replaced with antibody diluent to generate delete. Secondary antibodies were donkey anti-goat 488, goat anti-rabbit 488, and donkey anti-mouse 594. Scale bar = 50 μm. (TIF 4438 kb) [file 13058_2019_1117_MOESM4_ESM.tif]

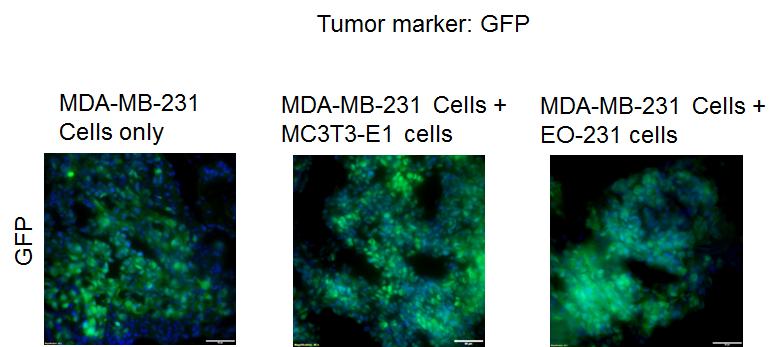

Supplement: Supplementary file 5 — Figure S5. Intratibial injection tumor formation with MDA-MB-231, MC3T3-E1, and EO Cells. Athymic nude mice were injected via intratibial injection with an admix of MDA-MB-231GFP/Luc2 human breast cancer cells plus either EO-231 cells or MC3T3-E1 osteoblasts, or MDA-MB-231GFP/Luc2 cells alone. Eight weeks later, mice were euthanized and their tibia harvested. Sections were stained for green fluorescent protein via immunofluorescence. Scale bar = 50 μm. (TIF 1109 kb) [file 13058_2019_1117_MOESM5_ESM.tif]

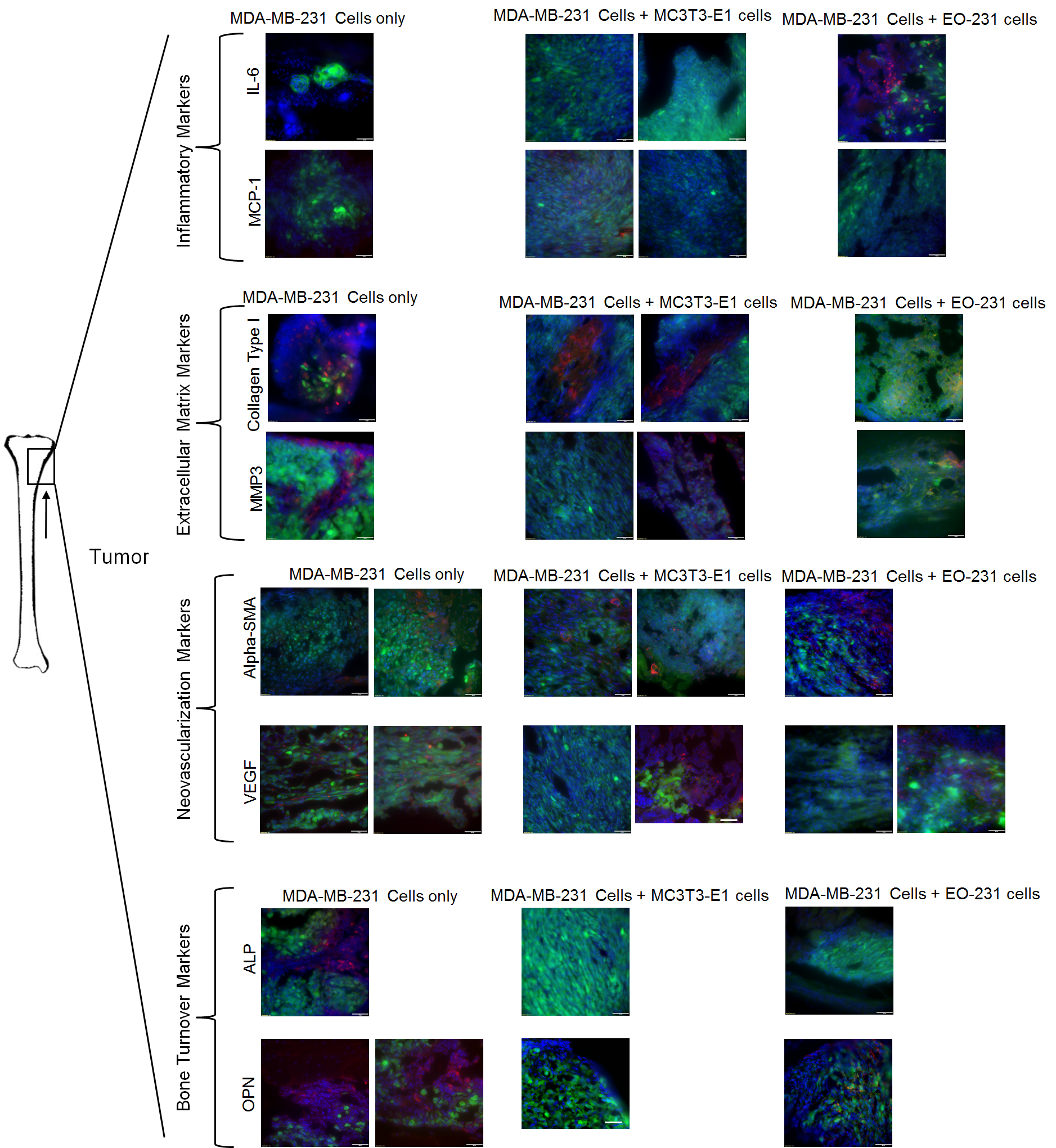

Supplement: Supplementary file 6 — Figure S6. Unique protein expression occurs with EO cell presence in tumor-bearing bones. Athymic nude mice were injected via intratibial injection with an admix of MDA-MB-231GFP/Luc2 human breast cancer cells plus either EO-231 cells or MC3T3-E1 osteoblasts, or MDA-MB-231GFP/Luc2 cells alone. Eight weeks later, mice were euthanized and their tibia harvested. Tibia sections from athymic mice were prepared as described in the “Materials and methods” section. Sections were stained for osteopontin, alkaline phosphatase, VEGF, alpha-smooth muscle actin, MMP3, collagen type I, MCP-1, IL-6, and green fluorescent protein via immunofluorescence. As shown on the tibia at left, the black box represents the positioning of the tumor in the examples shown and illustrates locations in the bone where the images were taken. At least three independent, serial sections were stained per bone, and three bones examined per condition. Shown are representative images. The tumor microenvironment was examined via fluorescent microscopy. Scale bar = 50 μm. (TIF 15079 kb) [file 13058_2019_1117_MOESM6_ESM.tif]

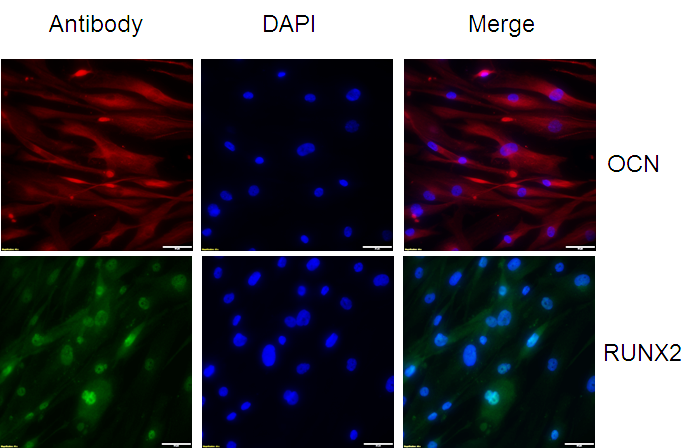

Supplement: Supplementary file 7 — Figure S7. OCN and RUNX2 antibody optimization using NHOst human osteoblasts. NHOst cells were grown to confluence then fixed and stained for expression of osteocalcin (red, 594) and RUNX2 (green, 488) by immunofluorescence. Scale bar = 50 μm. (TIF 1206 kb) [file 13058_2019_1117_MOESM7_ESM.tif]

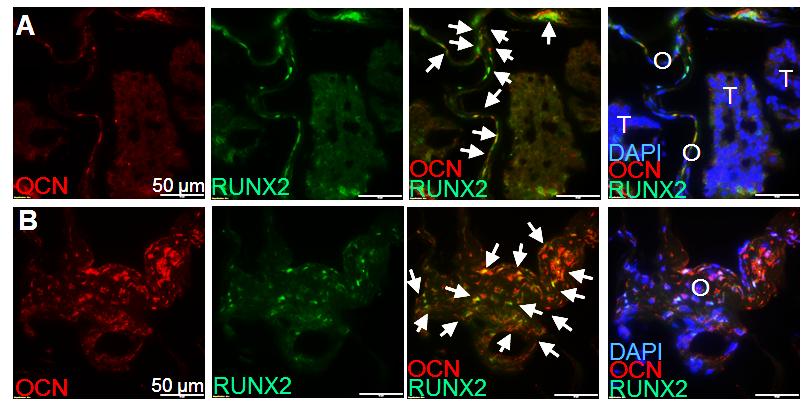

Supplement: Supplementary file 8 — Figure S8. Osteoblasts are present in patient samples of bone metastatic breast cancer. Human patient samples of bone metastatic breast cancer were stained using immunofluorescence for RUNX2 (green, 488) and osteocalcin (OCN, red, 594). Osteoblasts were identified A) adjacent to tumor cells and B) away from tumor cells. T = tumor; O, arrows =osteoblast. DAPI, nuclear stain. Scale bar = 50 μm. (TIF 1441 kb) [file 13058_2019_1117_MOESM8_ESM.tif]

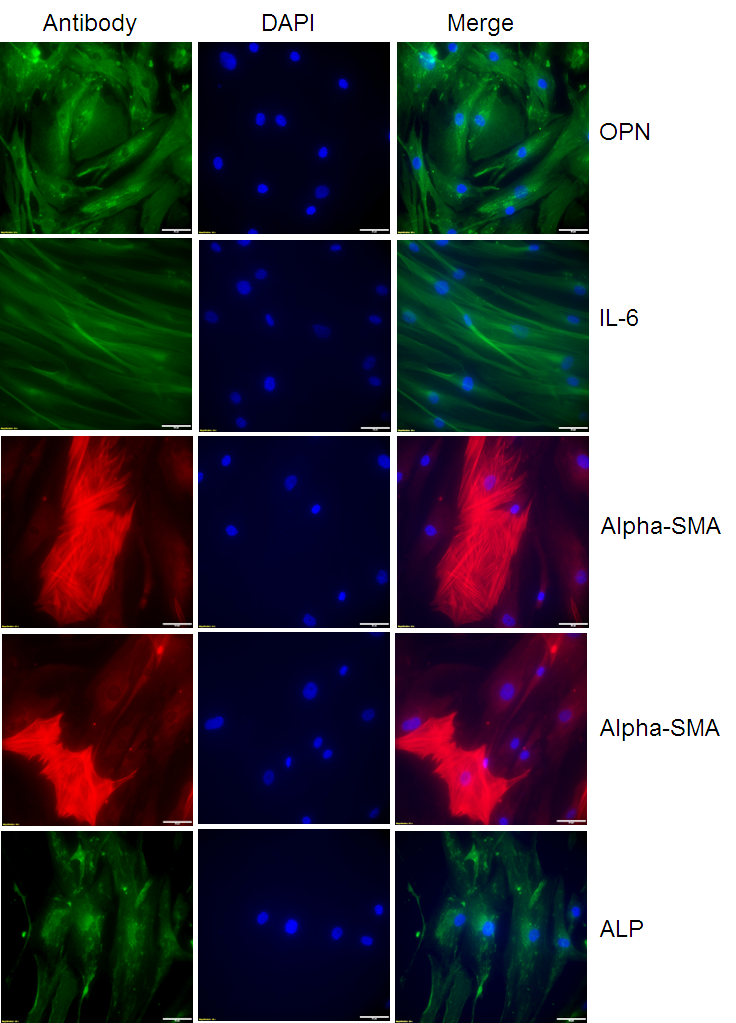

Supplement: Supplementary file 9 — Figure S9. Antibody optimization using NHOst human osteoblasts. NHOst cells were grown to confluence then fixed and stained for expression of osteopontin (green, 488), IL-6 (green, 488), alpha-SMA (red, 594), and alkaline phosphatase (green, 488) by immunofluorescence. Scale bar = 50 μm. (TIF 2887 kb) [file 13058_2019_1117_MOESM9_ESM.tif]

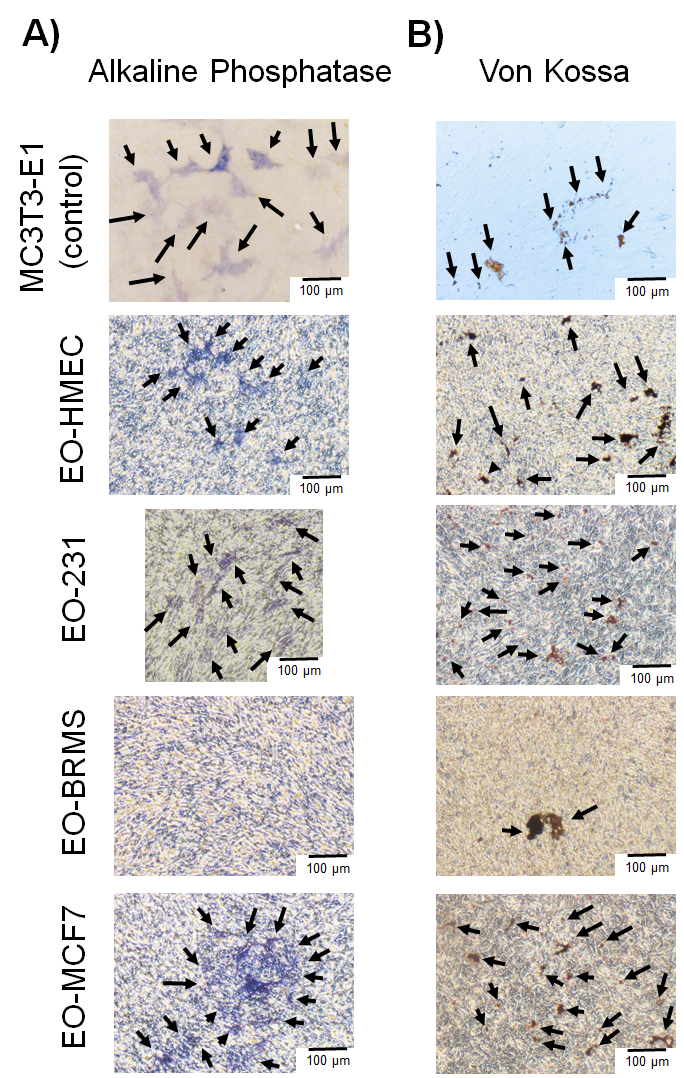

Supplement: Supplementary file 10 — Figure S10. EO cells differentiate and mineralize. EO cells were plated at 1 × 10^5^ cells/cm2 in 35 × 10 mm dishes, then grown to confluence. MC3T3-E1 cells were plated in growth media at 1 × 10^5^ cells/cm2 in 35 × 10 mm dishes. Twenty-four hours later, growth media were removed from MC3T3-E1 cells, and replaced with differentiation media. MC3T3-E1 cells were differentiated for 20 days. For both cell groups, media were exchanged every third day. Once cells reached confluence (EO cells) or were differentiated to 20 days (MC3T3-E1 cells), media were removed, cells fixed, then stained for either alkaline phosphatase expression using Napthol AS-BI phosphate, Tris, and Fast Blue RR salt; or Von Kossa using silver nitrate. Cells were photographed using a light microscope. Three biological replicates were completed per condition. Shown are representative images. (TIF 3499 kb) [file 13058_2019_1117_MOESM10_ESM.tif]

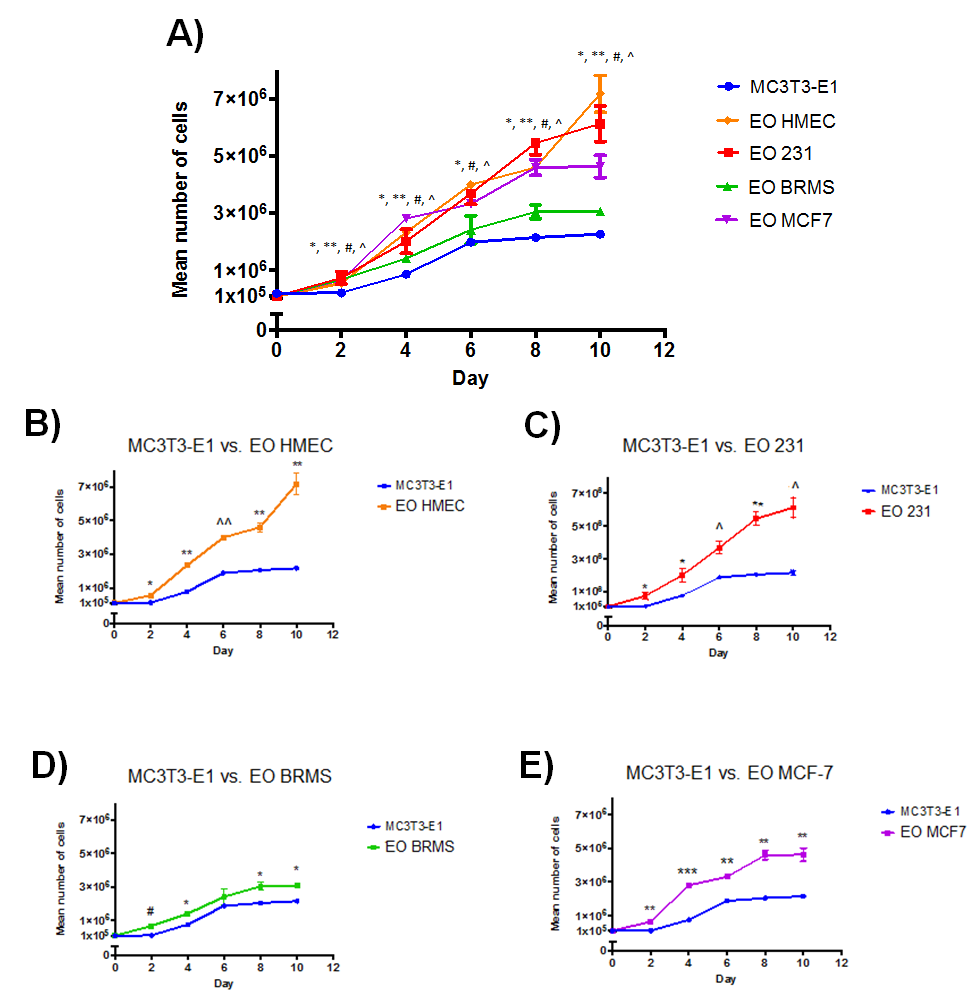

Supplement: Supplementary file 11 — Figure S11. EO cells have altered rates of proliferation compared to normal osteoblasts. Vehicle-treated MC3T3-E1, EO-HMEC, EO-231, EO-BRMS, or EO-MCF7 cells were plated at 1 × 10^5^ cells/cm2 in 35 × 10 mm dishes. Three individual replicates per condition were plated. On days 2, 4, 6, 8, and 10, cells were detached and counted using a hemocytometer. A) Statistical significance was calculated at each time point (i.e., days 2, 4, 6, 8, and 10) and represented by *P < 0.05 EO-HMEC vs. vehicle-treated MC3T3-E1, **P < 0.05 EO-231 vs. vehicle-treated MC3T3-E1, #P < 0.05 EO-BRMS vs. vehicle-treated MC3T3-E1, ^P < 0.05 EO-MCF7 vs. vehicle-treated MC3T3-E1. B) MC3T3-E1 vs. EO-HMEC, *P < 0.05, ^^P < 0.01, **P < 0.005; C) MC3T3-E1 vs. EO-231, *P < 0.05, ^P < 0.01, **P < 0.005; D) MC3T3-E1 vs. EO-BRMS, *P < 0.05, #P < 0.0005; E) MC3T3-E1 vs. EO-MCF7, *P < 0.05, **P < 0.005, ***P < 0.0001. (TIF 3171 kb) [file 13058_2019_1117_MOESM11_ESM.tif]

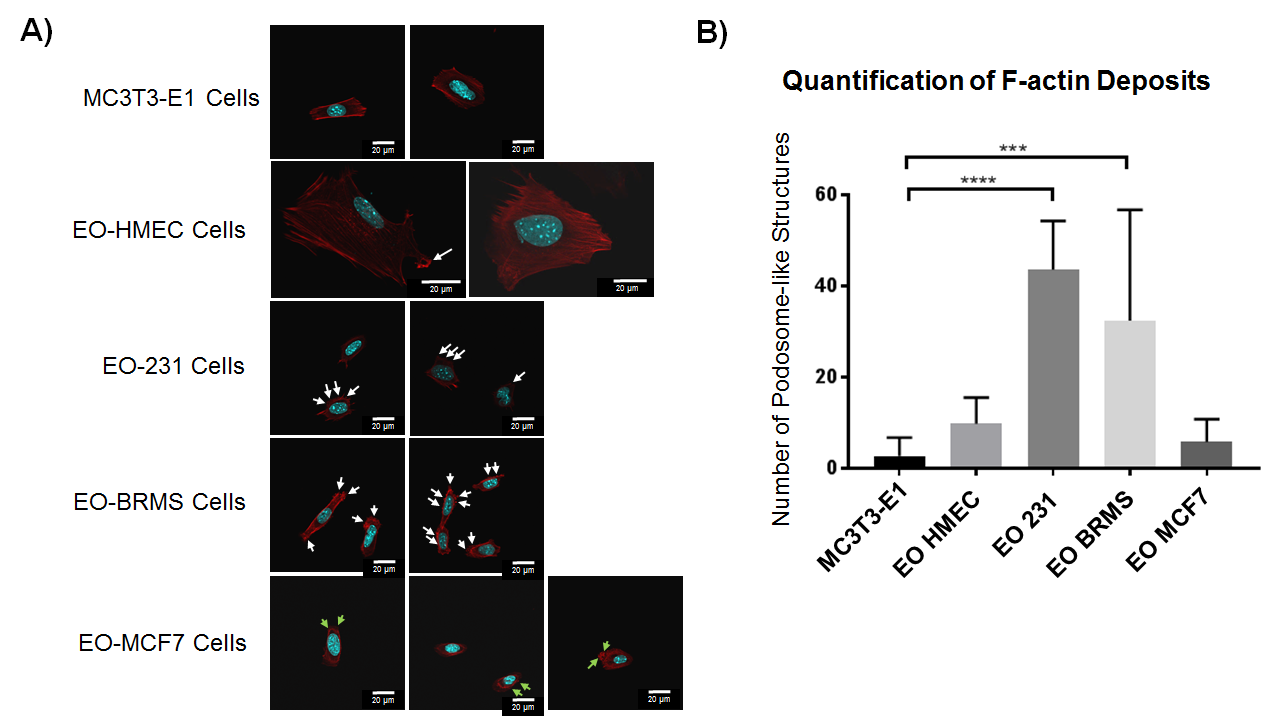

Supplement: Supplementary file 12 — Figure S12. EO cells have altered F-actin organization compared to normal osteoblasts. A) MC3T3-E1 cells were plated in 35 × 10 mm2 dishes at 1 × 10^5^ cells/cm2 in growth media. Cells were grown to ~ 70% confluence. Growth medium was exchanged every third day. EO cells were plated in 35 × 10 mm dishes at 1 × 10^5^ cells/cm2 and grown in three parts 1.5× differentiation medium plus 1 part either MDA-MB-231, MDA-MB-231BRMS, or MCF-7 breast cancer-conditioned medium or hTERT-HME1 mammary epithelial cell-conditioned medium. Cells were grown to ~ 70% confluence. Media were changed every second day. For F-actin staining, media were removed, cells washed with PBS, then cells stained for F-actin expression using a phalloidin stain. Three biological replicates were carried out per condition. Shown are representative images. Arrows point to F-actin deposits. B) Quantification of F-actin deposits. ***P = 0.0004, ****P < 0.0001. (TIF 3065 kb) [file 13058_2019_1117_MOESM12_ESM.tif]

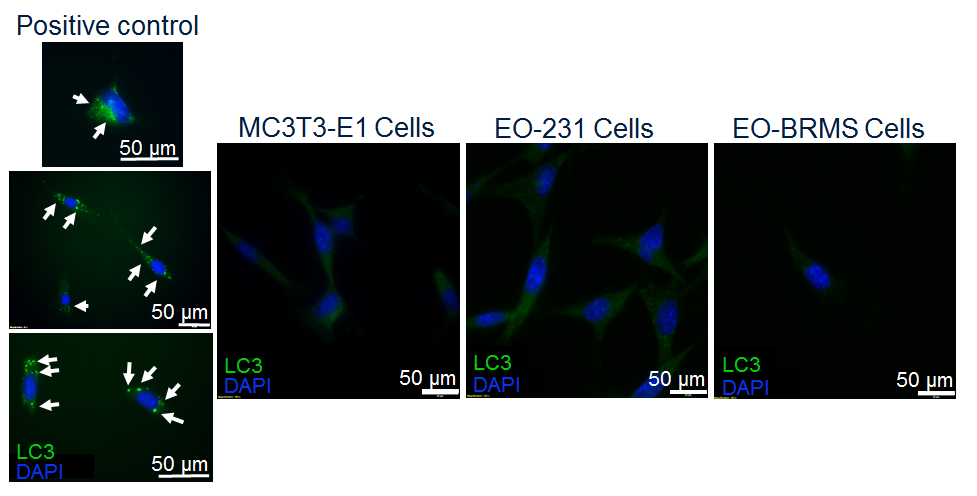

Supplement: Supplementary file 13 — Figure S13. EO cells do not exhibit autophagic lysosomes. MC3T3-E1, EO-231, and EO-BRMS cells were plated in 35 × 10 mm dishes, then stained for LC3 expression via immunofluorescence. MC3T3-E1 cells treated with serum-free media for 48 h served as a positive control. N = 3 dishes per condition. Shown are representative images. (TIF 1721 kb) [file 13058_2019_1117_MOESM13_ESM.tif]

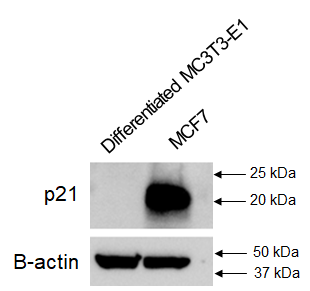

Supplement: Supplementary file 14 — Figure S14. Human-specific p21 antibody does not cross-react with murine cells. Murine MC3T3-E1 osteoblasts differentiated to 20 days and human MCF-7 lysates were collected and analyzed for human-specific p21 by western blot. N = 2 biological replicates per condition. (TIF 268 kb) [file 13058_2019_1117_MOESM14_ESM.tif]

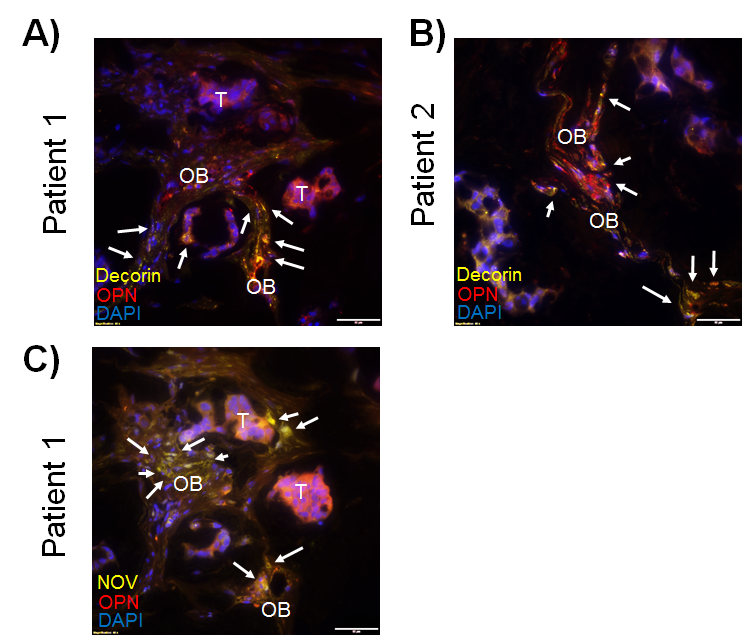

Supplement: Supplementary file 15 — Figure S15. NOV and decorin are expressed in the tumor-bearing bones of human patients with bone metastatic breast cancer. Serial sections of human patient samples of bone metastatic breast cancer were stained using immunofluorescence for decorin (yellow), NOV (yellow), and osteopontin (OPN, red). A) Patient 1 staining for decorin; B) patient 2 staining for decorin; C) patient 1 staining for NOV. N = 2 slides stained per protein using serial sections. At least three patient samples were stained per protein. Shown are representative examples. OB = osteoblast; T = tumor; DAPI, nuclear stain. Scale bar = 50 μm. (TIF 2012 kb) [file 13058_2019_1117_MOESM15_ESM.tif]

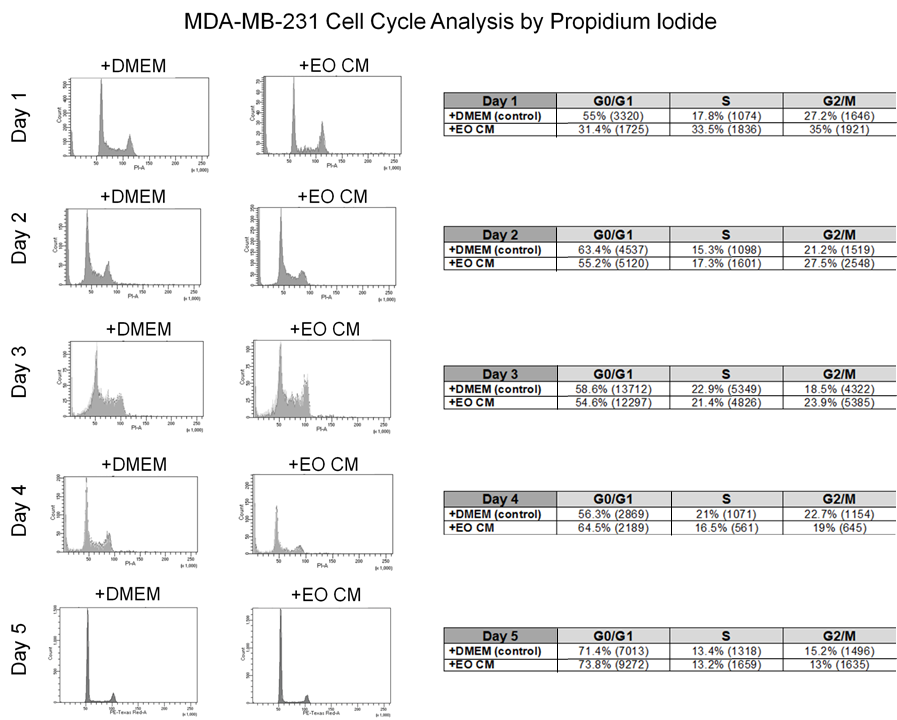

Supplement: Supplementary file 16 — Figure S16. Altered cell growth of MDA-MB-231 cells treated with EO CM is due to altered cell cycle. MDA-MB-231 cells were plated at 1 × 10^5^ cells/cm2 in 35 × 10 mm dishes. Twenty-four hours later, growth media were removed and cancer cells treated with 1 ml breast cancer growth media plus either a) vehicle media (DMEM, control) or b) EO-conditioned media. Breast cancer cells were fixed for at least 2 h with 95% cold ethanol beginning on day 1 after plating, and continuing every day for 5 days. For propidium iodide staining, ethanol was decanted and fixed cells washed once with PBS. Cells were resuspended in a solution of 50 ng/ml propidium iodide, 100 ng/ml RNAse A, and PBS per 1 × 10^6^ cells and incubated for 30 min in the dark at room temperature. Stained cells were analyzed for propidium iodide staining using a BD LSRII flow cytometer at excitation 535 nm and emission at 617 nm. A minimum of 10,000 events were counted per sample. Cell cycle phase was analyzed using BDFACS Diva software and FlowJo software. Three biological replicates per time point were counted per condition. Shown are representative images. (TIF 2281 kb) [file 13058_2019_1117_MOESM16_ESM.tif]

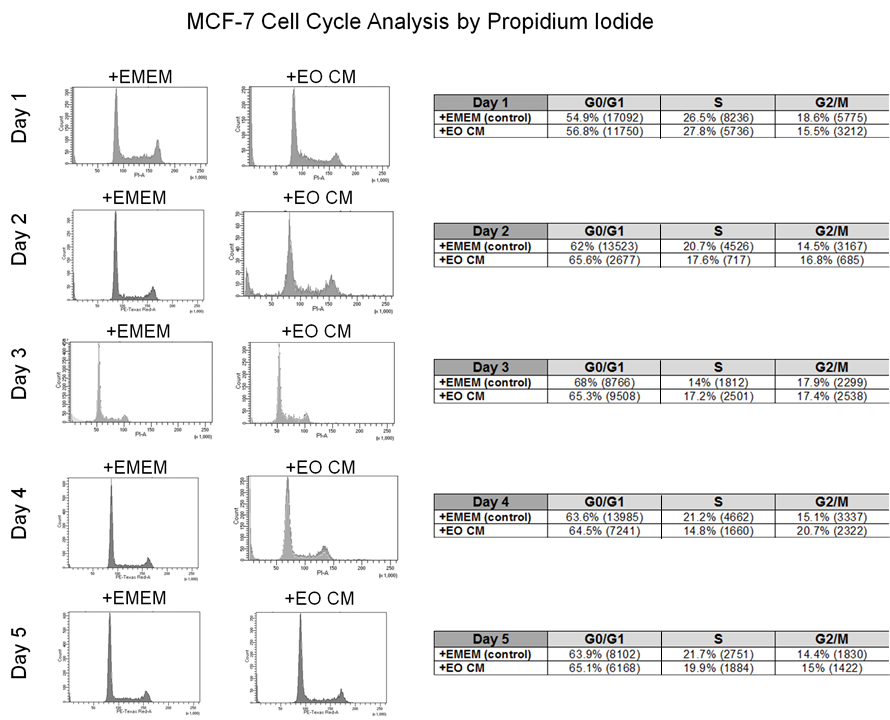

Supplement: Supplementary file 17 — Figure S17. Altered cell growth of MCF-7 cells treated with EO CM is due to altered cell cycle. MCF-7 cells were plated at 1 × 10^5^ cells/cm2 in 35 × 10 mm dishes. Twenty-four hours later, growth media were removed and cancer cells treated with 1 ml breast cancer growth media plus either a) vehicle media (EMEM, control) or b) EO-conditioned media. Breast cancer cells were fixed for at least 2 h with 95% cold ethanol beginning on day 1 after plating, and continuing every day for 5 days. For propidium iodide staining, ethanol was decanted and fixed cells washed once with PBS. Cells were resuspended in a solution of 50 ng/ml propidium iodide, 100 ng/ml RNAse A, and PBS per 1 × 10^6^ cells and incubated for 30 min in the dark at room temperature. Stained cells were analyzed for propidium iodide staining using a BD LSRII flow cytometer at excitation 535 nm and emission at 617 nm. A minimum of 10,000 events were counted per sample. Cell cycle phase was analyzed using BDFACS Diva software and FlowJo software. Three biological replicates per time point were counted per condition. Shown are representative images. (TIF 2263 kb) [file 13058_2019_1117_MOESM17_ESM.tif]
